# Factors determining species richness patterns of breeding birds along an elevational gradient in the Horn of Africa region

**DOI:** 10.1002/ece3.5491

**Published:** 2019-08-05

**Authors:** Ahunim Fenitie Abebe, Tianlong Cai, Melaku Wale, Gang Song, Jon Fjeldså, Fumin Lei

**Affiliations:** ^1^ Key Laboratory of the Zoological Systematics and Evolution, Institute of Zoology Chinese Academy of Sciences Beijing China; ^2^ Department of Biology, Faculty of Natural and Computational Sciences Assosa University Assosa Ethiopia; ^3^ University of Chinese Academy of Sciences Beijing China; ^4^ College of Sciences Bahir Dar University Ethiopia; ^5^ Center for Macroecology, Evolution and Climate, Natural History Museum of Denmark University of Copenhagen Copenhagen Denmark; ^6^ Center for Excellence in Animal Evolution and Genetics Chinese Academy of Sciences Kunming China

**Keywords:** breeding birds, climate, elevational gradients, horn of Africa, mountain, productivity, species richness

## Abstract

**Aim:**

To document the species richness patterns of breeding birds along elevational gradients and explore its drivers in the Horn of Africa region.

**Location:**

Horn of Africa region.

**Taxon:**

Breeding birds.

**Methods:**

Distributional data for breeding birds were collected. Elevational distribution data were extracted, interpolated, and assembled for all birds, passerines, and nonpasserines. In order to tease apart how different environmental factors contributed to the variation in species richness, we found it is necessary to divide the area into four subregions with different climatic regimes and topographic structure, namely western slope, eastern slope, wet side, and dry side. Then, the species richness in each 100‐m elevational band was counted along the elevational gradients of each subregion. Pearson's correlation analyses and ordinary least squares (OLS) regressions were used to examine the relationships between species richness and factors.

**Results:**

The variation in species richness followed hump‐shaped patterns for all subregions, although with peak values at different elevations. The bird species groups on the western and eastern slopes showed low and high plateaus with mid‐elevation peaks, respectively, but very low species diversities at the highest elevations. Species richness was significantly correlated with temperature range and productivity in each subregion. The temperature range, area, and productivity explained 82% of the species richness variations for all birds on the western slope.

**Main conclusions:**

The separate analyses of four area subdivisions provide strong indications of how various factors interact. Productivity and temperature range were the major driving factors for shaping the richness patterns, but deviations from expected patterns suggest modifying roles of mist formation zones in the valleys that deeply intersect the large highlands in the west and rich riparian vegetation where water from cool and humid environments at high elevation reaches lower elevations in the arid east. Conservation is recommended in each subregion based on the elevational richness scenarios.

## 1 INTRODUCTION

Understanding the variation in species richness in mountain regions, which occupy 50% of all global biodiversity hotspots, has been the main challenge for conservationists, biogeographers, and ecologists for the last two decades (Marchese, 2015). Mountain regions are important biodiversity areas where new species are incubated and accumulated in stabile climate and an absence of anthropogenic habitat conversion (Fjeldså, 2018; Hawkins et al.., 2003; Quintero & Jetz, 2018). Over the last few decades, variation of species diversity with elevation has become an attractive research area (McCain, 2009; Rahbek, 2005).

Species richness is well known to decline as we move away from the equator along the latitudinal gradient (Field, 2002). The elevational gradient was assumed to mirror this pattern, with higher diversity in the lowland and lower diversity in the cold highlands, until Rahbek (1995) demonstrated that the classical study by Terborgh (1977) actually contained evidence for a mid‐elevation peak in species richness. Recently, many researchers have organized their work and documented species richness along elevational gradients around the world for many groups of plants and animals and discovered a variety of patterns in variations of species richness (Lee, Ding, Hsu, & Geng, 2004; McCain, 2005; McCain & Grytnes, 2010), including lack of elevational gradient patterns (Fierer et al., 2011). According to Rahbek (2005), the most common type of species richness pattern is hump‐shaped (50%), and other researchers also confirmed this pattern for birds (Lee et al., 2004; Wu et al., 2013), amphibians (Hu, Xie, Li, & Jiang, 2011; Hutter, Guayasamin, & Wiens, 2013), mammals (McCain, 2005), and ants (Bishop, Robertson, Rensburg, & Parr, 2014). However, some studies found a species richness pattern that monotonically decreases for birds (McCain, 2007; Quintero & Jetz, 2018).

After discovering these different species richness patterns along elevational gradients, researchers have tried to identify factors that shape these patterns across different mountains and taxa with different climates and evolutionary histories. Although there are some main testable hypotheses, the common cause that accounts for species richness variation along elevational gradients for different geographical areas and taxa is still not detected.

Temperature limits the number of species that can survive at different locations, and elevations act on the physiology of species or may limit the number of individuals indirectly by restricting productivity, which in turn limits the population sizes and total number of individuals (Brown, 2001; Hawkins et al., 2003). A positive relationship has been established between temperature and species richness for many large‐scale diversity patterns (Hawkins et al., 2003) but not always true along elevational gradients, except in reptiles, whose elevational distribution is strongly constrained by temperature (McCain, 2010). A positive relationship between precipitation and species richness has been observed in local and regional diversity patterns (Hawkins et al., 2003). The precipitation hypothesis predicts that species diversity increases with increasing precipitation (Rosenzweig, 1992), though the relationship between species diversity and precipitation varies from region to region. Indirectly, precipitation affects productivity trends that in turn affect species diversity (Gentry, 1988).

The productivity hypothesis states that species diversity increases with productivity (Bailey et al., 2004), but there is some contradiction to this as other research suggests that after a certain point, increasing productivity actually correlates with a decrease in diversity (Grytnes, 2003). Traditionally, productivity has been assumed to decline with elevation (Rosenzweig, 1992), but many studies have demonstrated a peak in productivity at mid‐elevation, corresponding to a local peak in water availability. Another hypothesis predicts that the positive relationship between diversity and productivity is due to the ability of highly productive areas to support more individuals within a community, and thus, more species (Srivastava & Lawton, 1998). Alternatively, high productivity may result in increased availability of critical resources and therefore support more species.

The area hypothesis states that larger areas can support more species. In mountains, the species–area relationship predicts that sections of the montane gradient covering more area, for example, the mountain base, should harbor more species than regions with less area near the mountain tops (Ferenc et al., 2015; McCain, 2007; Rahbek, 1997).

However, the above climatic hypotheses may work best for conically shaped mountains with a uniform climate. The Horn of Africa, described as one of the global biodiversity hotspots of the world (Marchese, 2015), or as the “roof” of Africa (Behrens, 2010), receives humid air from the Atlantic Ocean and dry air from the Indian Ocean, resulting in great climatic differences between the west and the east. The western slope, affected by humid air and high evaporation from the swampy plains of the Sudd in South Sudan, is therefore humid, while the eastern slope, in contrast, is affected by dry air, although with some condensation of atmospheric humidity at the highest elevations (Friis, Demissew, & Breugel, 2010). Other differences are due to the topography, which affects the position of mist zones and flow of water from humidity‐capturing ridges into the valleys, or adjacent lowlands, and it is therefore virtually impossible to identify the key factors explaining the mid‐elevation diversity peak in a single analysis. However, the study region offers extraordinary opportunities for testing different hypotheses by comparing subregions with different topographies and climates. The western slope part includes a large highland and high plateau intersected by deep valleys with distinct local climate and zones of montane cloud forest, while the eastern slope is smooth in topography. However, dividing the area into a western and an eastern slope is complicated by the fact that the Bale Mountains and Harenna Forest in the east‐draining part receive precipitation from the west. Thus, wet and dry side divisions are important to find the effect of climatic factors.

There is no published analysis of variation in species richness across the Horn of Africa region, including of factors driving the variation along elevational gradients, other than a botanical study (Friis & Lawesson, 1993) and a global study of variation of avian species diversity on elevational gradients (Quintero & Jetz, 2018), which includes data from our study region. Taxon diversity is the number of all taxonomical groups of birds at different geographical and environmental variations. These variations, including the proportion and distribution of different avian orders, may contribute to differences in richness patterns among subregions, for instance, because passerine birds are generally small and therefore more able to maintain local populations on the more restricted area that is available near the mountain summits, and we could therefore assume that passerines show higher degrees of local speciation with the complex and structured montane landscapes like western slope than the more wide‐ranging nonpasserine birds. Therefore, we had documented species richness patterns and assessed the roles of area, climate, and productivity in explaining the elevational patterns of species richness among different species groups by dividing the Horn of Africa into four subregions mainly based on topography and precipitation.

## 2 METHODS

### 2.1 The study area

**Figure 1:**
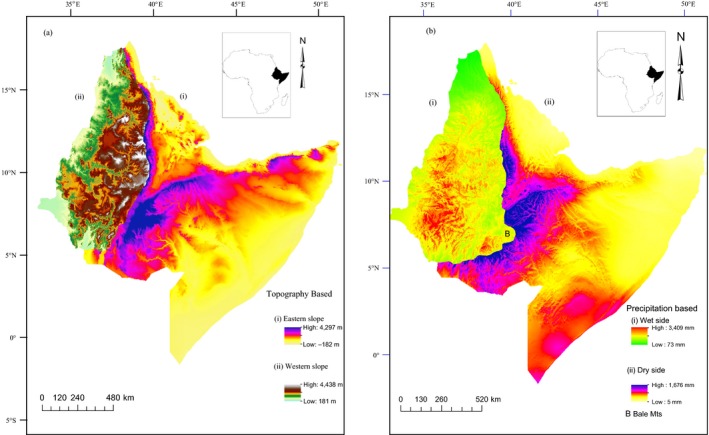
Map of the Horn of Africa region, to the left the topography‐based subdivision of eastern and western slopes and to the right the precipitation‐based subdivision of the wet and dry sides

The Horn of Africa is located in the eastern part of the African continent, jutting out into the Arabian Sea (Indian Ocean) to the south of the Arabian Peninsula. The region lies immediately to the north of Kenya and east of South Sudan and the Republic of Sudan. In the north, the Red Sea and the Gulf of Aden form the northeastern boundary, and in the east, it is bordered by the Indian Ocean. The Horn of Africa, also sometimes known as northeast Africa, lies between the equator and the Tropic of Cancer, approximately within the latitudes of 2°S and 18°N, and extends west to the east between 34°E and 54°E. The region comprises four countries (Ethiopia, Eritrea, Djibouti, and Somalia) covering almost 1.9 million km^2^ (Redman, StevTenson, & Fanshawe, 2011). The mountainous part of the region has been classified as western and eastern highlands, separated by the Rift Valley. These highlands have different slopes in the west and east, as the eastern slope is generally steeper than the western (Friis et al., 2010; Figure 1). The eastern side slopes gently toward the southern and southeastern lowlands of Ethiopia and Somalia, toward the Indian Ocean; these areas range from altitudes of approximately 350 to 1,500 m. In the west, there is a fairly extensive plateau, deeply intersected by valleys that give rise to special cloud forest conditions, and with only a more narrow strip of lowland habitats at approximately 350–500 m altitude, on the border toward the plains of southern Sudan. The highlands of Ethiopia, on both sides of the Rift Valley, have a general elevation ranging from altitudes of approximately 1,500 to 3,000 m, but most areas are below 2,500 m. Scattered over the highlands are higher mountain massifs and volcanic cones that often reach altitudes over 4,000 m. The southwestern part of the Western Highlands is not as high as the northern section and is cut by many wide valleys which give complex topography.

This region has high endemism with more than 70 bird species being endemic (or near‐endemic) to this part of the continent (Linder et al., 2012), and it is a major wintering ground for Palearctic species in Africa during migration. Most of the endemics and near endemics can be encountered in Ethiopia, which has remained the most accessible country within the Horn of Africa (Birdlife International, 1998). The composition of the resident avifauna combines elements of several biomes, including the Sudan–Guinea Savanna biome, the Sahel biome, the Sahara–Sindian biome, the Afrotropical highlands biome, the Somali–Masai biome, and the East African Coast biome (Redman et al., 2011).

#### 2.1.1 Division of the study area

The Horn of Africa is dominated by the isolated mountain massif of central Ethiopia and Eritrea (the most extensive highland plateau in Africa), which is bisected by the Rift Valley. The highland blocks to either side of the Rift are frequently referred to as the Western Highlands (west and north of the Rift Valley) and the Southeastern Highlands (to the east). This highland is deeply intersected by valleys and has many peaks above 4,000 m, including the Simien Mountains. To the west, there are large swampy plains (the Sudd) in South Sudan and a seasonal climate that provides precipitation and high condensation and mist zones in the upper valleys, with cloud forest up to approximately 2,500 m (locally 3,000 m). Much of the lowlands in the east are arid or semiarid and very hot throughout the year, but with some condensation on the upper slopes, which means that the highlands are both cooler and lusher (Redman et al., 2011).

However, dividing the highland is complicated because the Bale Mountains (with Harenna Forest and other large forest tracts of foothill forest) in the southern part of the east‐draining slope are influenced by the wet air from the west (Uhlig, 1988). Since montane species' distributions are often structured by climatic gradients (McCain & Grytnes, 2010), and to properly analyse the role of different environmental factors, we had to make two subdivisions: one strictly based on topography and patterns of drainage, following the continental divide (Figure 1a; western slope and eastern slope), and one based on humidity (Figure 1b; dry side and wet side) to compare dry and wet mountains within the same biogeographical province (Figure 1). The western slope comprises the most humid part of the region while eastern slope comprises the most arid parts, but with high humidity in the Bale Mountain and the surrounding region, which receives precipitation from the west. On the other hand, the wet side comprises the southwestern Rift Valley that intersects the Highlands where the stratum of warm air is formed above cold air either because warm air rises up above cold air or because cold air from a highland sinks down into deep valleys (usually during the night, and then, the morning sun warms up the upper slopes and the cold air is therefore trapped in the valley), and then, the horizontal mist layer is formed in the valleys, on the transition between the cold and the warm air. This mist zone helps to maintain humidity in the valleys, even at times of the year when there is little rainfall. The dry side comprises the arid and smooth slope toward the eastern part of the region. These divisions of the region into four subregions are important for exploring how different environmental variables contribute to the species richness patterns. Most of the study area is found in the ecosystems of Ethiopia and neighboring countries, which are similar to the nearest Ethiopian ecosystem, and the diversity of vegetation types in Ethiopia is considered to be an ecosystem. According to Friis et al. (2010) and Getahun (2018), the Ethiopian ecosystem is classified into eight ecosystems.

### 2.2 Data collection

For bird taxonomy, we followed the IOC World Bird List v8.1 (Gill & Donsker, 2018). Checklists of breeding bird species in the Horn of Africa are based on Redman et al. (2011) (Table S1). We collected the available occurrence and elevational range data for breeding birds integrating museum records for Ethiopia; electronic databases such as GBIF (https://www.gbif.org), eBird (https://ebird.org), and Xeno‐Canto (https://www.xeno-canto.org); published articles (Appendix 1); and field observations and private collections (Table S1). Most of the collected data were in the form of decimal degrees (DD; 97.6%), while some of the data were elevation ranges, degrees/minutes/seconds (DMS), and location names. DMS and location names were converted into DD. After removing multiple entries referring to the same species record, all geo‐referenced species records were mapped to visualize geographical locations for all breeding birds in the Horn of Africa at an appropriate resolution (1 km × 1 km), using ArcGIS 10.2 (ESRI, USA) to overlay the distributional data, and separated as east and west datasets based on topography and precipitation.

#### 2.2.1 Elevational species richness

Using the geo‐referenced species distribution data, the elevational data of each species were extracted from a digital elevation model (DEM) using a geospatial modeling environment (GME; Beyer, 2012). We constructed four subregional datasets, namely the western slope, eastern slope, wet side, and dry side, with three taxonomic groupings (all birds, passerines, and nonpasserines).

Since regional data often need standardizing or sampling evaluations, we interpolated the species range between its highest and lowest reported elevations (Grytnes & Romdal, 2008) because interpolation is important to overcome some limitations of undersampling and is regarded as valid for vagile species and allows methodological consistency because most published accounts have assumed range continuity (McCain, 2009; Wu et al., 2013). Each species is assumed to be present or potentially present between its highest and lowest reported elevations (range interpolation). We also used a rarefaction curve method to check sampling effort in each subregion (Gotelli & Colwell, 2001; Sanders, 1968) including our personal bird‐watching experience and by using the local bird‐watching guides in the Horn of Africa by considering low data sampling from below 1,000 m in the western foothills and high density of records from around the wetlands in the Rift Valley and in the eastern foothill zone. Adjusting records at only a single elevation (thus having a recorded elevational range value = 0) by adding 100 m to each side of the recorded elevation is important, following the strategy of previous studies (Brehm, Colwell, & Kluge, 2007; Cardelús, Colwell, & Watkins, 2006), so that each of these species was assumed to have an elevational range of 200 m. Then, species richness was calculated based on the number of birds occurring in each 100‐m elevational band (e.g., 100–199.9 m) for each dataset in each subregion.

#### 2.2.2 Predictors

We calculated the annual mean temperature (AMT), hereafter “temperature”; annual precipitation (AP), hereafter “precipitation”; and mean temperature annual range (TAR), hereafter “temperature range,” in each elevational band based on monthly records from Climatologies at High Resolution for the Earth's Land Surface Areas (CHELSA)—free climate data at high resolution (30 arc seconds; http://chelsa-climate.org/) for the years 1979–2013 (Karger et al., 2017). The mean temperature annual range is the difference between the warmest and coldest months, that is, June and December, respectively.

We used a 30‐arc second (approximately 1 km) digital elevation model (DEM) from GTOP30 downloaded from USGS to calculate the land surface area in our study area. Then, we projected the surface area layer onto the elevation layer and calculated the total area of the land surface in each elevational band in intervals using R version 3.4.3 (R Development Core Team, 2017).

We used the normalized difference vegetation index (NDVI) and enhanced vegetation index (EVI) to represent primary productivity in our study area. For estimates of productivity, MODIS‐driven NDVI and EVI images, composited at 16‐day intervals, were downloaded from USGS for 11 years (2000–2010) of data from the Horn of Africa, which had a spatial resolution of 1 km (Huete et al., 2002). We excluded 2011 and 2012 productivity data because of severe droughts in these years and occasionally after those years. Therefore, an 11‐year average NDVI and EVI in each elevational band interval were calculated using R. To resolve cloud cover problems, we took the maximum value for each elevational band.

### 2.3 Data analysis

Before any statistical analysis, we performed a normality test using Shapiro–Wilk and Anderson–Darling tests (Razali & Wah, 2011; Shapiro & Wilk, 1965). All response variables and all explanatory variables were normally distributed except AMT and TAR. Thus, AMT and TAR data were square‐transformed to meet assumptions of normality. The homoscedasticity test was also checked using residuals versus predicted values of variables using scatter plots. The relationship between species richness and independent variables was also checked (Table S2). The presence of spatial autocorrelation (as revealed by Moran's I) had been tested along elevational gradient. Multicollinearity of explanatory variables can cause failures in regression analyses (MacNally, 2002). We performed variance inflation factors (VIF) for all data and used variables with VIF (VIF < 10; Neter, Wasserman, & Kutner, 1984). Then, we exclude the variables that showed multicollinearity during multiple regression model selection. We used Neyman–Pearson correlation to examine the relationships among predictor variables (area, AMT, AP, TAR, NDVI, and EVI).

To evaluate the potential of the explanatory power of each factor on interpolated species richness, we performed parametric single linear regressions (Table S3), which is important for identifying multicollinear variables used in multiple regression. To evaluate the potential of the combined explanatory power of factors on interpolated species richness, we used multiple ordinary least squares (OLS) regression (Table 3). We took either NDVI or EVI during multiple regressions depending on the relationship with species richness and individual magnitude variation from single linear regression results because they have high correlation in each dataset. The lowest Akaike information criterion (AIC) value was used to select the best model at a significant level (*α* = 0.05) from all 58 models (Tables S5–S16). We also ran the interaction effect of variables on the species richness patterns (Table 2). We performed polynomial regressions between species richness and elevation to assess the form of elevational distribution patterns of species richness for each species group (Table S4).

We used SPSS version 20 for the VIF and Pearson's correlation; R software version 3.4.3 (R Development Core Team, 2017) for OLS regression; and PAST 3.14 (Hammer, Harper, & Ryan, 2001) for the normality test, linearity test, polynomial regression, and graph representation.

## 3 RESULTS

From the whole region, we found 626 breeding bird species belonging to 26 orders, 90 families, and 279 genera. Among these, 533 breeding birds had data available, representing 26 orders, 87 families, and 254 genera. A total of 520 bird species were found on the eastern slope against 493 birds on the western slope (480 species were shared between the two slopes), while 503 bird species were found on the dry side and 507 on the wet side of the region (477 species were shared between the two sides). Bird records are available from 400 to 4,300 m on both the western slope and wet side and from sea level to 3,700 m on both the eastern slope and dry side.

### 3.1 Elevational patterns of species richness

**Figure 2:**
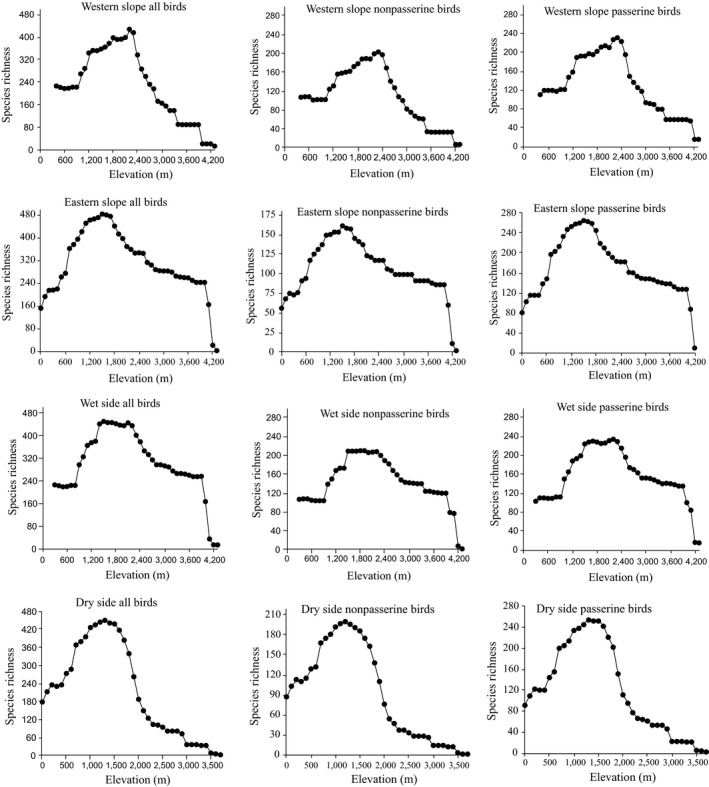
Different species richness patterns for breeding bird groups in the Horn of Africa region

On the eastern slope, the species richness follows a hump‐shaped pattern with a rapidly increasing diversity up to a peak at c. 1,200–1,700 m and a moderate decrease up to 4,000 m (Figure 2). The eastern slope diversity is high in the Rift Valley toward the Bale Mountain slopes, including Ahmar Mountain and the Negele and Yabelo hotspots, and it is the species richness in these areas that causes the plateau of species richness up to 4,000 m shown in Figure 2. The species richness on the western slope also follows a hump‐shaped pattern with a constant level of 220 species up to 1,000 m, then a marked increase up to a peak at 2,200 m followed by a rapid decrease above that level (Figure 2). This means that the species richness increases from 1,000 m and the diversity is very high in the cloud forest zone in the valleys and on the escarpments toward the large plateau, with a sharp drop in diversity as soon as the plateau is reached. With respect to the precipitation‐based subregions, the dry side and the wet side both showed hump‐shaped species richness patterns (Figure 2), with peaks ranging from 1,200 to 1,500 m and 1,400 to 2,300 m, respectively. However, the diversity curve indicates that the Bale Mountain areas are now included in the western (humid) zone (see Figure 1). All datasets showed hump‐shaped species richness patterns, although with small differences in the location of the peak and a more prominent variation in the pattern of decline toward the highest elevations. The polynomial regression analyses between elevation and species richness patterns along the elevational gradients clearly showed a hump‐shaped pattern in most of the species groups—mostly better fit by quadratic and above function of elevation than a simple linear regression (Table S3).

Passerines and nonpasserines show virtually identical patterns, with the most notable difference being the relatively low diversity of nonpasserines above 3,500 m on the western slope, against relatively higher levels at these elevations on the eastern slope (Figure 2). On the western slope, eastern slope, and dry side, both passerines and nonpasserines contributed almost equally to the shape of species richness. However, on the wet side, the passerines contributed more strongly to the overall species richness pattern.

**Figure 3:**
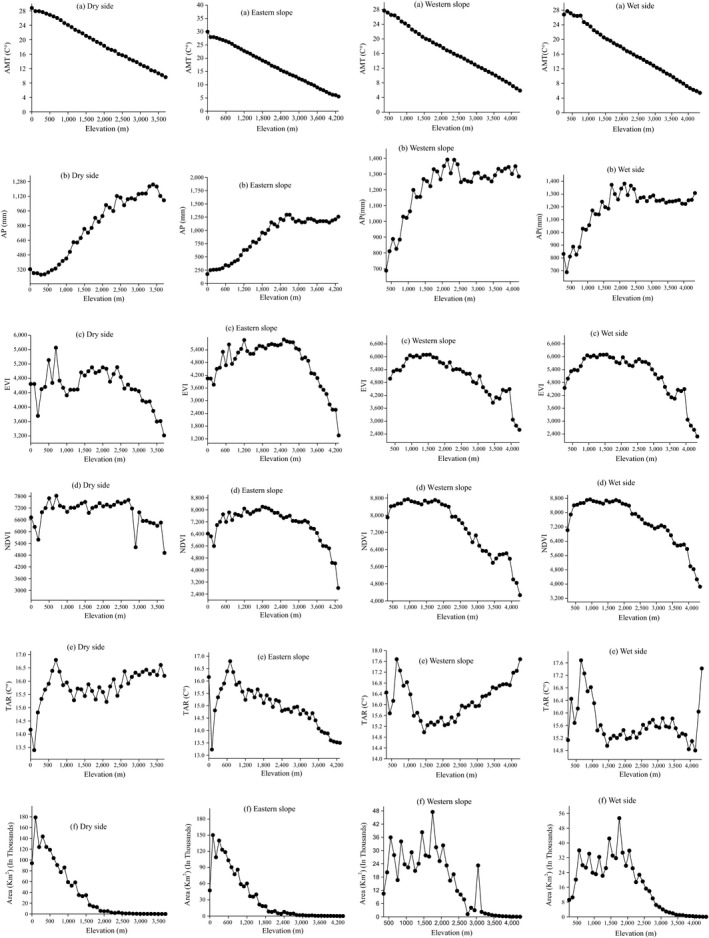
The relationship between elevation and predictors at all study regions in the Horn of African region. AMT, annual mean temperature; AP, annual precipitation; EVI, enhanced vegetation index; NDVI, normalized difference vegetation index; TAR, mean temperature annual range

Based on single linear regression, temperature decreases by 0.6°C/100 m in the Horn of Africa region in all subregions (*r* ^2^ = .999, *p* < .000; Figure 3). Productivity (EVI and NDVI) showed a hump shape with a decreasing pattern as elevation increased, but it is important to note that high EVI and NDVI values in the dry lowlands represent only very localized patches of riparian habitat (Google Earth image, 2018). Precipitation increased almost linearly with elevation and remained constant at the middle and then decreased slightly at the top elevations in all subregions. However, TAR varied among subregions; for example, on the western slope, it showed almost a parabola opening upward; that is, the variation is at both ends of the elevational gradient (Figure 3).

### 3.2 The relationship between species richness and explanatory variables

Bivariate analyses were done between species richness and factors (without considering autocorrelation; Table S2) and between factors (Table 1). Based on these bivariate analyses, we found species richness was positively related to temperature range, area, and productivity (EVI) in western slope birds; temperature range and productivity (NDVI) in eastern slope birds; temperature range and productivity (EVI) in wet side birds; and area and productivity (EVI) in dry side birds, again, with minor differences between passerines and nonpasserines in all subregions (Table S2). Multiple regressions were run to predict species richness from the above variables for all subregions and significantly predicted species richness at *p* < .05. Based on a coefficient of determination (*R* ^2^), the temperature range, productivity (EVI), and area determined the species richness patterns stronger than other variables for all species groups on the western slope subregion. Temperature range and productivity (NDVI) determined the species richness patterns on eastern slope and wet side subregions, while area and productivity determined the species richness patterns on the dry side subregion (Table 3). In addition to the main effect of variables on species richness patterns, interaction effect also has significant effects on species richness patterns in each subregion and species groups (Table 2). So, the effect of combined variables was different from the effect of interacted variables in most subregions along the elevational gradient.

**Table 1:** Pearson correlation coefficients for environmental variables used in models to analyze the species richness pattern of breeding birds in the Horn of African region

Western slope							Eastern slope					
	AMT	AP	EVI	NDVI	TAR	Area	AMT	AP	EVI	NDVI	TAR	Area
AMT												
AP	−.855**						−.966**					
EVI	.578**	−.306					.269	−.096				
NDVI	.801**	−.411**	.741**				.358*	−.217	.957**			
TAR	−.144	−.275	−.450**	−.594**			.690**	−.622**	.633**	.703**		
Area	.678**	−.267	.553**	.843**	−.536**		.893**	−.920**	.049	.137	.437**	

Wet side							Dry side					
	AMT	AP	EVI	NDVI	TAR	Area	AMT	AP	EVI	NDVI	TAR	Area
AMT												
AP	−.967**						−.800**					
EVI	−.144	.039					.566**	−.083				
NDVI	−.290	.164	.876**				.729**	−.262	.950**			
TAR	.236	−.108	−.622**	−.616**			−.124	−.094	−.368*	−.318*		
Area	.675**	−.783**	.206	.129	−.399*		.647**	−.126	.731**	.819**	−.165	

Abbreviations: AMT, annual mean temperature; AP, annual precipitation; EVI, enhanced vegetation index; NDVI, normalized difference vegetation index; TAR, mean temperature annual range.

** *p* < .01.

* *p* < .05.

**Table 2:** Interaction effects of environmental variables on the species richness patterns of breeding birds in the Horn of Africa region

Species groups	Interacted variables	*R* ^2^	C	*F*	*df*		*p*
Western slope all birds	TAR × EVI	.8688	−3.810 × 10^–02^	79.45	3	36	6.084 × 10^–16^
Western slope passerine birds	Area × EVI	.6874	−1.533 × 10^–06^	26.39	3	36	3.328 × 10^–09^
Western slope nonpasserine birds	TAR × Area	.819	1.365 × 10^–03^	54.31	3	36	1.927 × 10^–13^
Eastern slope all birds	TAR × NDVI	.773	0.03662	45.41	3	40	5.995 × 10^–13^
Eastern slope passerine birds	TAR × NDVI	.7134	2.351 × 10^–02^	32.36	3	39	1.132 × 10^–10^
Eastern slope nonpasserine birds	TAR × NDVI	.7725	0.010227	45.29	3	40	6.252 × 10^–13^
Wet side all birds	TAR × Area × AP	.7214	4.508 × 10^–5^	12.21	7	33	1.387 × 10^–07^
Wet side passerine birds	NDVI × AMT × Area	.9435	−1.755 × 10^–08^	76.3	7	32	<2.2 × 10^–16^
Wet side nonpasserine birds	NDVI × AMT × Area	.9603	−1.573 × 10^–08^	114	7	33	<2.2 × 10^–16^
Dry side all birds	TAR × AP × AMT	.9245	−1.734 × 10^–03^	52.45	7	30	4.265 × 10^–15^
Dry side passerine birds	TAR × AP × AMT	.9221	−9.288 × 10^–04^	50.75	7	30	6.7 × 10^–15^
Dry side nonpasserine birds	TAR × AP × AMT	.9264	−8.111 × 10^–04^	53.97	7	30	2.883 × 10^–15^

Abbreviations: AMT, annual mean temperature; AP, annual precipitation; EVI, enhanced vegetation index; NDVI, normalized difference vegetation index; TAR, mean temperature annual range.

## 4 DISCUSSIONS

### 4.1 Elevational patterns of species richness

The Horn of Africa mountain region is one of the global biodiversity hotspots (Linder et al., 2012; Marchese, 2015). Previous studies of variation with elevation and among ecoregions have mainly focused on the drivers of buildup plants species richness, including environmental and anthropogenic factors (van Breugel, Friis, Demissew, Lillesø, & Kindt, 2016; Friis & Lawesson, 1993). Recently, Quintero and Jetz (2018) assessed elevational species richness in a global scale, suggesting that different regions have different driving factors. Comparison of the ecoregional diversity differences within the hotspot has rarely been reported.

The magnitude of the relationship (*r*) between species richness and predictors was not found to be the same, and lack relationships with some predictors (*p* > .05; Table S2). The lack of linearity in some predictors reflects the number of local anomalies in deep valleys that intersect the highlands; here, the local topography will create zones of anomalous temperature and humidity (from semi‐stable local mist zones) that are not reflected in the precipitation model and not at all in the theoretical model for linear lapse‐based drops in temperature with elevation. High species richness in some valleys can reflect benign temperatures and water supply from rivers (even in foothill zones with low precipitation). Temperature and precipitation showed a positive relationship with species richness, but the effect was not as close (Table S3). This might be driven by geographical complexity, especially on the wet side of the region. On the western slope and wet side subregions, species richness peaks are congruent with a low temperature range and high precipitation that may correspond to a zone of semipermanent mist effect that allows for permanent humidity just below the escarpment toward the high plateau.

Species richness patterns showed hump‐shaped patterns with peaks at mid‐elevation, although slightly different elevations were observed in different subregions. Western slope birds peaked at 2,100–2,300 m with a small range, while wet side birds peaked at 1,400–2,300 m with a large range because these elevation ranges (especially 1,500–3,400 m) have a large number of Afromontane plant species at these elevations and a distinction between a lower and a higher altitude Afromontane forest tree flora (Friis & Lawesson, 1993). Species richness patterns are known to differ between montane regions, with a low‐elevation plateau being most common (Quintero & Jetz, 2018), as found here for the eastern slope subregion, probably reflecting the high diversity of birds associated with riparian vegetation in the foothills and Rift Valley lakes. Even though a high amount of precipitation is also recorded from 2,100 to 2,500 m and is considered a good opportunity for species richness, in the Bale Mountains, there is high precipitation (humidity from the west) at high elevation, which maintains fairly high species richness up to 4,000 m, but the peak (1,600 m) of species diversity is found below the zone of high precipitation, probably because species diversity is highly concentrated in local places with mist effects or where rivers from the rainy highlands provide a stable water supply locally in the warm lower montane zone. Presumably, species diversity is highly concentrated in lush local environments and is not particularly strongly correlated with the recorded precipitation (which does not include mist effects) or the modeled temperature curve.

In other subregions, dry side birds peaked at 1,000–1,500 m because of the large number of lowland forest species in the mountain foothills south of the Bale Mountains to Harenna Forest, which reflects a distinction between lowland and transitional flora (Friis & Lawesson, 1993). Eastern slope birds peaked at 1,300–1,700 m with a plateau of fairly high species diversity all the way up to 4,000 m due to the humid zone of the Bale Mountains.

The mid‐elevation peak in bird richness is driven by wide‐ranging species and high rates of past temperature change, indicating the importance of climatic fluctuations in driving the evolutionary dynamics of mountain biodiversity. However, when subsampled, species richness patterns decrease as elevation increases (Quintero & Jetz, 2018). In contrast, the accumulation of many species (with different histories) in montane regions is first driven by stability in temperature and water availability in certain elevational zones. Such climatic stability may also allow bird populations to adapt and specialize in response to the environmental pressure generated by local conditions within the montane habitat mosaics. This is why mountains can only accumulate high numbers of endemic species at low latitudes, where the seasonality is slight (Fjeldså, 2018).

On the western and eastern slopes, both passerines and nonpasserines show virtually identical hump‐shaped patterns, suggesting that this pattern holds for different taxonomic groups and generally for small and large birds. Pomeroy and Ssekabiira (1990) reported that nonpasserines are more broadly distributed than passerines. In our study, dry and wet sides had almost equal numbers of nonpasserines. Pomeroy (1993) and de Klerk, Crowe, Fjeldså, and Burgess (2002) found similar species richness patterns for both passerines and nonpasserines in sub‐Saharan Africa region.

However, the fact that the wet side has more passerines than the dry side might reflect the presence of complex topography that creates local benign zones for many cloud forest passerines and also the presence of forest in the humid highlands. A predominance of open habitat in the dry regions is suitable for nonperching and widespread nonpasserine birds. This is different from western slope and eastern slope species richness patterns may be due to the effect of including and excluding the Bale Mountains and Harenna Forest.

### 4.2 The role of climatic factors

Climatic variables (temperature and precipitation) influence patterns of bird species richness directly or indirectly (H‐Acevedo & Currie, 2003). As the elevation increases, temperature decreases linearly (see Figure 3; Barry, 2008; McCain & Grytnes, 2010). However, this model assumes a uniform mountain slope. The western Ethiopian Highland has complex mountainous landscapes with deep valleys and gorges intersecting large highlands, which gives rise to complex patterns with atmospheric inversions and accumulation of cold air in some valleys, with a thermal belt on the upper slope, and such anomalies will cause local mist zones, which add humidity to the recorded precipitation and support the development of a distinct zone of cloud forest on the upper escarpments toward the high plateau. This explains the distinct peak of bird diversity at approximately 2,200 m elevation on the western slope. Because of this, the correlation with temperature is very low on the western slope and absent on the eastern slope and on the wet side (Table S3).

The temperature range varied along elevational gradients in different subregions (Figure 3). On the western slope, the temperature range was high at both ends of the gradient (low and high elevation), possibly reflecting seasonal variation in the western lowlands near Sudan and the Western Highlands, including mountains such as Simien, Choke, and Abune Yoseph, and this correlates with reduced diversity of all birds (Table 3). The eastern slope temperature range decreases with elevation (Figure 3), reflecting a strong mist effect and stable humidity in the Bale Mountains, which explains why species richness can remain high even up to 4,000 m (Figure 2). On the wet side, the temperature range is almost similar to the western slope. On the dry side, there is a high seasonality at all elevations, which may explain why diversity peaks at fairly low elevations where it is warm but lush vegetation and permanent water supply along rivers still exist. The bird diversity can be high in cold and misty places, as long as permanently humid conditions can be maintained. Therefore, climatic stability may be the main factor explaining the accumulation of high species diversity over a long time period in certain places in tropical mountains (Fjeldså, 2018).

**Table 3:** Best multiple OLS regression model (*p < *.05) for all breeding bird groups in the Horn of African region selected from all combinations using the smallest AIC value (Tables S5–S16) and linear correlation of variables with species richness (Table S2)

Species groups	Model	Variables	*R* ^2^	$R_{\text{adj}}^{2}$	*F*	*df*		*p*
Western slope all birds	46	TAR + Area + EVI	.8236	.8089	56.04	3	36	1.22 × 10^–13^
Western slope passerine birds	46	TAR + Area + EVI	.7977	.7808	47.32	3	36	1.42 × 10^–12^
Western slope nonpasserine birds	46	TAR + Area + EVI	.6864	.661	27	3	37	9.98 × 10^–10^
Eastern slope all birds	6	TAR + NDVI	.7558	.7442	65.01	2	42	1.39 × 10^–13^
Eastern slope passerine birds	6	TAR + NDVI	.7026	.6881	48.44	2	41	1.59 × 10^–11^
Eastern slope nonpasserine birds	6	TAR + NDVI	.768	.7569	69.5	2	42	4.75 × 10^–14^
Wet side all birds	29	TAR + EVI	.7373	.7238	54.73	2	39	4.78 × 10^–12^
Wet side passerine birds	29	TAR + EVI	.6493	.6308	35.18	2	38	2.26 × 10^–09^
Wet side nonpasserine birds	29	TAR + EVI	.7089	.6939	47.48	2	39	3.55 × 10^–11^
Dry side all birds	26	Area + EVI	.4111	.3793	12.92	2	37	5.57 × 10^–05^
Dry side passerine birds	26	Area + EVI	.3766	.342	10.87	2	36	0.0002022
Dry side nonpasserine birds	26	Area + EVI	.4369	.4064	14.35	2	37	2.43 × 10^–05^

Abbreviations: EVI, enhanced vegetation index; NDVI, normalized difference vegetation index; TAR, mean temperature annual range.

Precipitation varies greatly along elevational gradients (McCain & Grytnes, 2010), while in the present study, precipitation increased with elevation on the eastern slope and dry side, while it exhibited a hump‐shaped pattern on the western slope and wet side (Figure 3). Precipitation was significantly correlated with all species groups on wet and dry sides because this division totally separates the dry from the wet, not such as the western and eastern slope division, which allocated the wet part (Bale Mountains) to the eastern slope. The relationship is positive in the wet side and negative in the dry side. Thus, precipitation plays a great role in shaping the species richness patterns on wet and dry sides. Cueto and de Casenave (2014) indicated that bird richness distribution was determined by climatic conditions such as gradients of precipitation. However, precipitation, as measured for the different elevational zones, is not a crucial predictor for elevational diversity (Pan et al., 2016). The present study also supports this, since precipitation does not show any relation in western and eastern slope subregions for all species groups, probably because the water flows down along the valleys to lower and warmer zones, providing good local conditions in zones of rich riparian vegetation, and because the humidity caused by condensation from fog in certain places in the montane valleys will not be manifested in the precipitation record. Overall, arid mountain slopes have a high diversity in the lower montane zone, despite low precipitation, most likely because the diversity is highly associated with riparian vegetation in the valleys.

### 4.3 The role of available space

Arrhenius (1921) published a paper that forms the starting point for understanding patterns of species richness in nature; it recognizes that as area increases, species richness also increases. This species–area relationship holds over the full range of scales in nature, from small experimental microcosms in the laboratory up to whole regions of the Earth (Smith et al., 2005). However, other studies provided slight or contradictory evidence (Benayas, José, & Manuel, 1999). In our study, the effect of area was variable (Table 3). This is the case with our results in the eastern slope subregion, which lack significant relationship, McCain (2007) and Pan et al. (2016) found that the area is not identified as a main driving force and is not a crucial factor in determining species richness. In our case, the main factor is that the diversity in a certain elevational zone is strongly influenced by very local “hotspots,” with many species restricted to places with riparian forest or cloud forest. However, presumably the very small areas above 2,500 m on the eastern slope and above 2,000 m on the dry side may restrict the species diversity that can be found. In general, even though there is a fairly large area of foothill habitat in the west, the few sampling sites in the western foothills and more sampling sites from wetlands in the Rift Valley and in the eastern foothill zone may have a potential effect on the relationships.

### 4.4 Productivity factor

Productivity is considered to represent the productivity of an ecosystem with some measurement differences and is a good determinant of species richness patterns at different scales (Garroutte, Hansen, & Lawrence, 2016; Lee et al., 2004). In our study area, productivity showed a hump‐shaped pattern with elevation and positively related to species richness. Thus, productivity determined the species richness patterns of all species groups along elevational gradients in all subregions (Tables 2 and 3). The species richness peaks were also congruent with bands that are rich in plants that show productivity plays a great role in shaping elevational species richness in all subregions (Figure 3). However, we have to consider the limitation that EVI/NDVI only reflects the greenness of vegetation but cannot discriminate between short and tall/multistratum vegetation. Therefore, there may be bias, especially on the dry side. In fact, the recorded maximum values for NDVI are very uniform from at least 500 m to 2,500 m, but species diversity has a marked peak at approximately 1,500 m, which may reflect the limited vegetation patches in the desert lowland.

Overall, the species richness patterns have a significant negative relationship with elevation except for the wet side subregion which lacks significance relationship. Generally, the environmental variables play a great role in the shape of bird species richness patterns in the Horn of Africa region.

### 4.5 Conservation in the subregions

Bird conservation is a global mission, but a critical situation exists in the tropics where many species have small geographical distributions associated with specific habitats. A primary goal of large‐scale conservation planning is to conserve as much biodiversity as possible with minimum investment (Myers, Mittermeier, Mittermeier, Fonseca, & Kent, 2000). This requires comparable and reliable estimates of species richness across large geographical scales (Ibáñez et al., 2006). Biodiversity hotspots are found in mountain ranges, and montane species are shifting their ranges in elevation in response to climate change. Protecting elevational gradients can helpfully capture montane biodiversity patterns and facilitate species range shifts (Elsen, Monahan, & Merenlender, 2018). Tropical ecosystems that support 87% of the world's bird species are generally under greater pressure from human population growth, agricultural expansion, and a host of related factors, and many of them are highly susceptible to habitat loss or climate change (Tobias, Sekercioglu, & Vargas, 2013). The Horn of Africa region is currently affected by both climate change and anthropogenic activities (Haileab, Demel, & Ensermu, 2014; Tobias et al., 2013). Among anthropogenic activities, land‐use legacies have more serious effects than climate change (Ameztegui, Coll, Brotons, & Ninot, 2016). In Ethiopia, land use by local people, such as inappropriate plowing, extensive grazing, inappropriate use of fire, and deforestation for agriculture, is a common problem (Anteneh, Temesgen, & Adefires, 2013; Haileab et al., 2014; van Breugel et al., 2016). Thus, beyond the statistics, conservation should be implemented on the ground by governmental policies and community participation at the local level using cultural norms and religion‐based concepts.

Since mountain ranges in Africa and Asia have the lowest elevational protection (Elsen et al., 2018), efforts to protect the 70 endemic birds of the Horn of Africa region (the roof of Africa) are an essential area for biodiversity conservation in the region (Redman et al., 2011). Establishing protected areas may also play crucial roles in securing species populations (Rayner, Lindenmayer, Wood, Gibbons, & Manning, 2014). Improving protection along elevational gradients may be particularly important when climate change occurs that alter elevational distributions of agriculture (Hannaha et al., 2013), human populations (Seto, Güneralp, & Hutyra, 2012), and natural resources (Vörösmarty, Green, Salisbury, & Lammers, 2000). The two most challenging tasks for conservation biologists are identifying the species‐rich areas that require protection and the factors that predict species richness patterns along elevational gradients. In biogeographical studies, patterns and peaks of species richness highlight areas that support the most species. Many of the most fundamental questions in conservation require knowledge of the geographical distributions (and ecological niche requirements) of individual species (Riddle, Ladle, Lourie, & Whittaker, 2011). In our case, conserving specific areas is particularly much needed in elevational gradients from 1,400 to 2,300 in the west and from lowlands to 1,700 m in the east. In general, we recommend establishing protected areas, using the distribution of birds, in each subregion that supports higher species numbers and the nearest peaks that showed diversity; in this case, east and west of the valleys and hotspots such as Bahir Dar, Yabelo, and Negele are important areas. International Union for Conservation of Nature (IUCN) II may be applied to conserve these areas (Dudley, 2008). On the other hand, land conversion by man is massive in the most ornithologically rich zones, with cloud forest remaining only in steep ravines and escarpments that need concentrated efforts for better land management. According to Sekercioglu, Schneider, Fay, and Loarie (2008), adaptation to higher elevation refuges is risky, but higher elevations play an essential role as a refuge for lowland biodiversity and diversification and endemism because of their isolation, so that tropical mountains offer hope for conservation because mountain habitats often remain relatively intact, at least at higher elevations. Therefore, mountains such as Simien, Choke, Bale, and Ahmar should be focal areas for conservation. In addition, in the Western Highlands, the Ethiopian Orthodox Tewahedo Church forests are very important refuges for populated agricultural areas using IUCN III (Dudley, 2008). For precise and appropriate conservation, further local research is needed, especially in complex montane environments, to identify the causal relationships between biodiversity and environmental factors that require new kinds of empirical data.

## 5 CONCLUSIONS

We tested factors that shape the species richness distribution patterns along elevational gradients in the Horn of Africa region by dividing the region based on topography and precipitation. The species richness patterns in each subregion showed hump‐shaped patterns with a slight difference in shape and peak positions. These differences come from the combined and interacted effect of environmental factors, especially productivity and variation of temperature. Conservation is highly recommended at species‐rich elevational gradients using different methods such as preparing protected area based on species peak area scenario, especially cloud forest zones which are under high deforestation using community participation.

## CONFLICT OF INTERESTS

We have no conflict of interests.

## AUTHOR CONTRIBUTIONS

F.L. and J.F. managed the study program; A.F., F.L., J.F., and T.C. conceived the idea and research arrangement; A.F., M.W., J.F., T.C., S.G., and F.L. analyzed the data, developed the presentation, and wrote the paper.

## Data Availability

Elevation range data used in this analysis are available at Dryad Digital Repository: https://doi.org/10.5061/dryad.h3tj5mc.

## Appendix 1

### Data sources

Agarnesh, D. & Subramanian, C. (2015). Studies on avian diversity in Angereb Forest and adjacent farm land with reference to rainy and post rainy seasons, Northwestern Ethiopia. *International Journal of Pure and Applied Zoology*, **3**, 2320–9577.

Elizabeth, Y., Afework, B. & Zerihun, W. (2000). Bird strike incidence at Addis Ababa Bole International Airport. *SINET: Ethiopian Journal of Science*, **2**, 215–229.

Nega, T. & Afework, B. (2008). Diversity and habitat association of birds of Dembia plain wetlands, Lake Tana, Ethiopia. *SINET: Ethiopian Journal of Science*, **31**, 1–10.

Esayas, K. & Bekele, A. (2011). Species composition, relative abundance and distribution of the Avian Fauna of Entoto Natural Park and Escarpment, Addis Ababa. *SINET: Ethiopian Journal of Science*, **34**, 113–122.

Gosaye, T. & Afework, B. (2011). *Avian diversity based on habitat difference at Geto and Gembejo areas, southwest Shoa, Ethiopia*. Thesis dissertation, 26–31.

Gebrecherkos, W. & Tilaye, W. (2012). A survey on birds of the Yayu Forest in Southwest Ethiopia. *SINET: Ethiopian Journal of Science*, **35**, 117–128.

Gove, A. D., Hylander, K., Nemomissa, S., Shimelis, A. & Enkossa, W. (2013). Structurally complex farms support high avian functional diversity in tropical montane Ethiopia. *Journal of Tropical Ecology*, **29**, 87–97.
