# Supplementary material for: Factors determining species richness patterns of breeding birds along an elevational gradient in the Horn of Africa region
Source: Ecol Evol. 2019 Aug 5;9(17):9609–23. doi: 10.1002/ece3.5491 (PMC6745872; doi:10.1002/ece3.5491)
Supplement: Supplementary file 1 [file ECE3-9-9609-s001.docx]

**Factors Determining Species Richness Patterns of Breeding Birds along an Elevational Gradient in the Horn of Africa Region**

Ahunim Fenitie Abebe^1, 2, 3^, Tianlong Cai ^1, 3^, Melaku Wale^4^, Gang Song^1^, Jon Fjeldså^5*^ and Fumin Lei^1, 3, 6 *^

^1^Key Laboratory of the Zoological Systematics and Evolution, Institute of Zoology, Chinese Academy of Sciences, Beijing, 100101, China,

^2^Department of Biology, Faculty of Natural and Computational Sciences, Assosa University, Assosa, Ethiopia

^3^University of Chinese Academy of Sciences, Beijing, 100049, China

^4^College of Sciences, Bahir Dar University, Ethiopia

^5^Center for Macroecology, Evolution and Climate, Natural History Museum of Denmark, University of Copenhagen, DK-2100, Copenhagen, Denmark

^6^Center for Excellence in Animal Evolution and Genetics, Chinese Academy of Sciences, Kunming, 650223, China

*Correspondence: Fumin Lei, Institute of Zoology, Chinese Academy of Sciences, Beijing 100101, China.E-mail: leifm@ioz.ac.cn

**Table S1** Species List and Data Sources

| SN | Order | Family | Common Name | *Scientific Name* | **References** | **Endemic** |
| --- | --- | --- | --- | --- | --- | --- |
| 1 | ACCIPITRIFORMES | Accipitridae | Shikra | *Accipiter badius* | eB*,G*,P* |  |
| 2 | ACCIPITRIFORMES | Accipitridae | Great Sparowhawk | *Accipiter melanoleucus* | F*,eB*,G*,X*,P* |  |
| 3 | ACCIPITRIFORMES | Accipitridae | Little Sparrowhawk | *Accipiter minullus* | F*,eB*,G*,M*,P* |  |
| 4 | ACCIPITRIFORMES | Accipitridae | Rufous-chested Sparrowhawk | *Accipiter rufiventris* | F*,eB*,G*,M*,P* |  |
| 5 | ACCIPITRIFORMES | Accipitridae | African Goshawk | *Accipiter tachiro* | F*,eB*,G*,X*,M*,P* |  |
| 6 | PASSERIFORMES | Acrocephalidae | African Reed Warbler | *Acrocephalus baeticatus* | eB*,G*,X* |  |
| 7 | PASSERIFORMES | Acrocephalidae | Lesser Swamp-Warbler | *Acrocephalus gracilirostris* | F*,eB*,G*,X* |  |
| 8 | GALLIFORMES | Numididae | Vulturine Guineafowl | *Acryllium vulturinum* | F*,eB*,G*,X* |  |
| 9 | CHARADRIIFORMES | Jacanidae | African Jacana | *Actophilornis africanus* | F*,eB*,G*,X*,P* |  |
| 10 | PSITTACIFORMES | Psittaculidae | Red-headed LoveB* | *Agapornis pullarius* | eB*,G*,P* |  |
| 11 | PSITTACIFORMES | Psittaculidae | Black-winged LoveB* | *Agapornis taranta* | F*,eB*,G*,X*,P* | E^†^ |
| 12 | PASSERIFORMES | Alaudidae | Greater Hoopoe-Lark | *Alaemon alaudipes* | eB*,G* |  |
| 13 | PASSERIFORMES | Alaudidae | Somali Short-toed Lark | *Alaudala somalica* | F*,eB*,G*,X* |  |
| 14 | CORACIIFORMES | Alcedinidae | Half-collared Kingfisher | *Alcedo semitorquata* | F*,eB*,G*,X* |  |
| 15 | ANSERIFORMES | Anatidae | Egyptian Goose | *Alopochen aegyptiaca* | F*,eB*,G*,X*,P* |  |
| 16 | PASSERIFORMES | Estrildidae | Cut-throat Finch | *Amadina fasciata* | F*,eB*,G*,X*,M* |  |
| 17 | GRUIFORMES | Rallidae | Black Crake | *Amaurornis flavirostra* | F*,eB*,G*,X*,P* |  |
| 18 | PASSERIFORMES | Ploceidae | Thick-billed/Grosbeak Weaver | *Amblyospiza albifrons* | F*,eB*,G*,M* |  |
| 19 | PASSERIFORMES | Alaudidae | Desert Lark | *Ammomanes deserti* | eB*,G*,X* |  |
| 20 | PASSERIFORMES | Ploceidae | Red-headed Weaver | *Anaplectes rubriceps* | F*,eB*,G*,X*,M* |  |
| 21 | ANSERIFORMES | Anatidae | Cape Teal | *Anas capensis* | eB*,G*,M* |  |
| 22 | ANSERIFORMES | Anatidae | African Black Duck | *Anas sparsa* | F*,eB*,G*,X*,M*,P* |  |
| 23 | ANSERIFORMES | Anatidae | Yellow-billed Duck | *Anas undulata* | F*,eB*,G*,X*,M*,P* |  |
| 24 | SULIFORMES | Anhingidae | African Darter | *Anhinga rufa* | F*,eB*,G*,P*, |  |
| 25 | PASSERIFORMES | Viduidae | Cuckoo-finch | *Anomalospiza imberbis* | eB*,G*,P* |  |
| 26 | PASSERIFORMES | Remizidae | Mouse-colored Penduline-Tit | *Anthoscopus musculus* | F*,eB*,G*,X* |  |
| 27 | PASSERIFORMES | Nectariniidae | Kenya Violet-backed Sunbird | *Anthreptes orientalis* | F*,eB*,G*,X* |  |
| 28 | PASSERIFORMES | Motacillidae | African Pipit | *Anthus cinnamomeus* | F*,eB*,G*,X*,P* |  |
| 29 | PASSERIFORMES | Motacillidae | Plain-backed Pipit | *Anthus leucophrys* | F*,eB*,G*,X*,P* |  |
| 30 | PASSERIFORMES | Motacillidae | Long-billed Pipit | *Anthus similis* | F*,eB*,G*,X*,M* |  |
| 31 | ACCIPITRIFORMES | Accipitridae | White-headed Vulture | *Trigonoceps* *occipitalis* | eB*,P* |  |
| 32 | PASSERIFORMES | Cisticolidae | Yellow-breasted Apalis | *Apalis flavida* | eB*,G*,X* |  |
| 33 | TROGONIFORMES | Trogonidae | Narina Trogon | *Apaloderma narina* | F*,eB*,G*,P* |  |
| 34 | APODIFORMES | Apodidae | Little Swift | *Apus affinis* | F*,eB*,G*,X*,P* |  |
| 35 | APODIFORMES | Apodidae | Horus Swift | *Apus horus* | eB*,G* |  |
| 36 | APODIFORMES | Apodidae | Nyanza Swift | *Apus niansae* | F*,eB*,G*,X*,M*,P* |  |
| 37 | ACCIPITRIFORMES | Accipitridae | Golden Eagle | *Aquila chrysaetos* | eB*,G* |  |
| 38 | ACCIPITRIFORMES | Accipitridae | Tawny Eagle | *Aquila rapax* | F*,eB*,G*,M*,P* |  |
| 39 | ACCIPITRIFORMES | Accipitridae | Verreaux's Eagle | *Aquila verreauxii* | F*,eB*,G*,P* |  |
| 40 | PELECANIFORMES | Ardeidae | Great Egret | *Ardea alba* | F*,eB*,G*,X* |  |
| 41 | PELECANIFORMES | Ardeidae | Goliath Heron | *Ardea goliath* | F*,eB*,G*,P* |  |
| 42 | PELECANIFORMES | Ardeidae | Black-headed Heron | *Ardea melanocephala* | F*,eB*,G*,X*,P* |  |
| 43 | PELECANIFORMES | Ardeidae | Purple Heron | *Ardea purpurea* | F*,eB*,G*,P* |  |
| 44 | PELECANIFORMES | Ardeidae | Squacco Heron | *Ardeola ralloides* | eB*,G*,X*,P* |  |
| 45 | OTIDIFORMES | Otididae | Arabian Bustard | *Ardeotis arabs* | F*,eB*,G*,X* |  |
| 46 | OTIDIFORMES | Otididae | Kori Bustard | *Ardeotis kori* | F*,eB*,G*,X* |  |
| 47 | STRIGIFORMES | Strigidae | Abyssinian Owl | *Asio abyssinicus* | F*,eB*,G* |  |
| 48 | STRIGIFORMES | Strigidae | Little Owl | *Athene noctua* | eB*,G* |  |
| 49 | ACCIPITRIFORMES | Accipitridae | African Cuckoo-Hawk | *Aviceda cuculoides* | eB*,G* |  |
| 50 | GRUIFORMES | Gruidae | Black Crowned-Crane | *Balearica pavonina* | F*,eB*,G*,P* |  |
| 51 | PASSERIFORMES | Platysteiridae | Eastern Black-headed Batis | *Batis minor* | F*,eB*,X*,M*,P* |  |
| 52 | PASSERIFORMES | Platysteiridae | Gray-headed Batis | *Batis orientalis* | F*,eB*,G*,X*,P* |  |
| 53 | PASSERIFORMES | Platysteiridae | Pygmy Batis | *Batis perkeo* | F*,eB*,G*,X* |  |
| 54 | PELECANIFORMES | Threskiornithidae | Wattled Ibis | *Bostrychia carunculata* | eB*,G*,X*,P*, | E^†^ |
| 55 | PELECANIFORMES | Threskiornithidae | Hadada Ibis | *Bostrychia hagedash* | F*,eB*,G*,X*,P* |  |
| 56 | PASSERIFORMES | Muscicapidae | Grayish Flycatcher | *Bradornis microrhynchus* | eB*,G*,M* |  |
| 57 | PASSERIFORMES | Muscicapidae | Pale Flycatcher | *Bradornis pallidus* | F*,eB*,G*,M* |  |
| 58 | PASSERIFORMES | Locustellidae | Little Rush-Warbler | *Bradypterus baboecala* | eB*G* |  |
| 59 | PASSERIFORMES | Locustellidae | Cinnamon Bracken-Warbler | *Bradypterus cinnamomeus* | F*,eB*,G*,X*,M*,P* |  |
| 60 | PASSERIFORMES | Ploceidae | White-billed Buffalo-Weaver | *Bubalornis albirostris* | eB*,G*,X*,M* |  |
| 61 | PASSERIFORMES | Ploceidae | Red-billed Buffalo-Weaver | *Bubalornis niger* | F*,eB*,G*,X*,M* |  |
| 62 | STRIGIFORMES | Strigidae | Cape Eagle-Owl | *Bubo capensis* | F*,eB*,G*,X*,P* |  |
| 63 | STRIGIFORMES | Strigidae | Grayish Eagle-Owl | *Bubo cinerascens* | F*,eB*,G*,X*,P* |  |
| 64 | STRIGIFORMES | Strigidae | Verreaux's Eagle-Owl | *Bubo lacteus* | F*,eB*,G*,X*,M*,P* |  |
| 65 | PELECANIFORMES | Ardeidae | Cattle Egret | *Bubulcus ibis* | eB*,G*,X*,P* |  |
| 66 | BUCEROTIFORMES | Bucorvidae | Abyssinian Ground-Hornbill | *Bucorvus abyssinicus* | F*,eB*,G*,P* |  |
| 67 | PASSERIFORMES | Buphagidae | Red-billed Oxpecker | *Buphagus erythrorynchus* | F*,eB*,G*,M*,P* |  |
| 68 | CHARADRIIFORMES | Burhinidae | Spotted Thick-knee | *Burhinus capensis* | F*,eB*,G* |  |
| 69 | CHARADRIIFORMES | Burhinidae | Senegal Thick-knee | *Burhinus senegalensis* | F*,eB*,G*,X* |  |
| 70 | CHARADRIIFORMES | Burhinidae | Water Thick-knee | *Burhinus vermiculatus* | eB*,G*,X* |  |
| 71 | ACCIPITRIFORMES | Accipitridae | Archer's Buzzard | *Buteo archeri* | eB* | E^†^ |
| 72 | ACCIPITRIFORMES | Accipitridae | Augur Buzzard | *Buteo auguri* | F*,eB*,G*,X*,M*,P* |  |
| 73 | ACCIPITRIFORMES | Accipitridae | Mountain Buzzard | *Buteo oreophilus* | F*,eB*,G*,M* |  |
| 74 | PELECANIFORMES | Ardeidae | Striated Heron | *Butorides striata* | eB*,G*,X* |  |
| 75 | BUCEROTIFORMES | Bucerotidae | Silvery-cheeked Hornbill | *Bycanistes brevis* | F*,eB*,G*,X*,M*,P* |  |
| 76 | PASSERIFORMES | Cisticolidae | Gray Wren-Warbler | *Calamonastes simplex* | F*,eB*,G*,X* |  |
| 77 | PASSERIFORMES | Alaudidae | Blanford's Lark | *Calandrella blanfordi* | eB*,G*,X* |  |
| 78 | PASSERIFORMES | Alaudidae | Erlanger's Lark | *Calandrella erlangeri* | F*,eB*,G*,X*,P* | E^†^ |
| 79 | PASSERIFORMES | Alaudidae | Foxy Lark | *Calendulauda alopex* | F*,eB*,G*,X* |  |
| 80 | CHARADRIIFORMES | Scolopacidae | Temminck's Stint | *Calidris temminckii* | F*,eB*,G*,X*,M* |  |
| 81 | PASSERIFORMES | Cisticolidae | Green-backed Camaroptera | *Camaroptera brachyura* | F*,eB*,G*,M*,P* |  |
| 82 | PASSERIFORMES | Campephagidae | Red-shouldered Cuckooshrike | *Campephaga phoenicea* | F*,eB*,G*,P* |  |
| 83 | PICIFORMES | Picidae | Nubian Woodpecker | *Campethera nubica* | F*,eB*,G*,X*,P* |  |
| 84 | CAPRIMULGIFORMES | Caprimulgidae | Slender-tailed Nightjar | *Caprimulgus clarus* | F*,eB*,G*,X*,M* |  |
| 85 | CAPRIMULGIFORMES | Caprimulgidae | Long-tailed Nightjar | *Caprimulgus climacurus* | eB*,G* |  |
| 86 | CAPRIMULGIFORMES | Caprimulgidae | Donaldson-Smith's Nightjar | *Caprimulgus donaldsoni* | F*,eB*,G*,X* |  |
| 87 | CAPRIMULGIFORMES | Caprimulgidae | Sombre Nightjar | *Caprimulgus fraenatus* | eB*,G*,X* |  |
| 88 | CAPRIMULGIFORMES | Caprimulgidae | Plain Nightjar | *Caprimulgus inornatus* | eB*,G* |  |
| 89 | CAPRIMULGIFORMES | Caprimulgidae | Nubian Nightjar | *Caprimulgus nubicus* | eB*,G* |  |
| 90 | CAPRIMULGIFORMES | Caprimulgidae | Abyssinian Nightjar | *Caprimulgus poliocephalus* | eB*,G*,X*,P* |  |
| 91 | CAPRIMULGIFORMES | Caprimulgidae | Star-spotted Nightjar | *Caprimulgus stellatus* | eB* |  |
| 92 | CAPRIMULGIFORMES | Caprimulgidae | Freckled Nightjar | *Caprimulgus tristigma* | eB*,G*,X* |  |
| 93 | PASSERIFORMES | Hirundinidae | Lesser Striped-Swallow | *Cecropis abyssinica* | F*,eB*,G*,X* |  |
| 94 | PASSERIFORMES | Hirundinidae | Red-rumped Swallow | *Cecropis daurica* | F*,eB*,G*,X*,M* |  |
| 95 | PASSERIFORMES | Hirundinidae | Mosque Swallow | *Cecropis senegalensis* | F*,eB*,G*,P* |  |
| 96 | CUCULIFORMES | Cuculidae | Blue-headed Coucal | *Centropus monachus* | F*,eB*,G*,X*,M*,P* |  |
| 97 | CUCULIFORMES | Cuculidae | Senegal Coucal | *Centropus senegalensis* | eB*,G*,P* |  |
| 98 | CUCULIFORMES | Cuculidae | White-browed Coucal | *Centropus superciliosus* | eB*,G*,X*,M*,P* |  |
| 99 | PASSERIFORMES | Turdidae | Sombre Chat | *Cercomela dubia* | eB*,G* | E^†^ |
| 100 | PASSERIFORMES | Muscicapidae | White-browed Scrub Robin | *Cercotrichas leucophrys* | F*,eB*,G*,X* |  |
| 101 | CORACIIFORMES | Alcedinidae | Pied Kingfisher | *Ceryle rudis* | F*,eB*,G*,P* |  |
| 102 | CORACIIFORMES | Alcedinidae | African Pygmy Kingfisher | *Ispidina picta* | F*,eB*,G*,P* |  |
| 103 | PASSERIFORMES | Nectariniidae | Hunter's Sunbird | *Chalcomitra hunteri* | F*,eB*,G*,X* |  |
| 104 | PASSERIFORMES | Nectariniidae | Scarlet-chested Sunbird | *Chalcomitra senegalensis* | eB*,G*,X*,M*,P* |  |
| 105 | CHARADRIIFORMES | Charadriidae | Kentish Plover | *Charadrius alexandrinus* | F*,eB*,G* |  |
| 106 | CHARADRIIFORMES | Charadriidae | Kittlitz's Plover | *Charadrius pecuarius* | F*,eB*,G*,X*,M*,P* |  |
| 107 | CHARADRIIFORMES | Charadriidae | Three-banded Plover | *Charadrius tricollaris* | eB*,G*,X*,P* |  |
| 108 | PASSERIFORMES | Muscicapidae | Spotted Palm Thrush | *Cichladusa guttata* | F*,eB*,G*,X*,P* |  |
| 109 | PASSERIFORMES | Sturnidae | Violet-backed Starling | *Cinnyricinclus leucogaster* | F*,eB*,G*,M*,P* |  |
| 110 | PASSERIFORMES | Nectariniidae | Purple-banded Sunbird | *Cinnyris bifasciatus* | eB* |  |
| 111 | PASSERIFORMES | Nectariniidae | Copper Sunbird | *Cinnyris cupreus* | eB*,G*,M*,P* |  |
| 112 | PASSERIFORMES | Nectariniidae | Shining Sunbird | *Cinnyris habessinicus* | eB*,G*,X*,P* |  |
| 113 | PASSERIFORMES | Nectariniidae | Mariqua Sunbird | *Cinnyris mariquensis* | F*,eB*,G*,X* |  |
| 114 | PASSERIFORMES | Nectariniidae | Black-bellied Sunbird | *Cinnyris nectarinioides* | F*,eB*,G*,X* |  |
| 115 | PASSERIFORMES | Nectariniidae | Beautiful Sunbird | *Cinnyris pulchellus* | F*,eB*,G*,X*,M*,P* |  |
| 116 | PASSERIFORMES | Nectariniidae | Variable Sunbird | *Cinnyris venustus* | eB*,G*,X*,M*,P* |  |
| 117 | ACCIPITRIFORMES | Accipitridae | Western Banded Snake-Eagle | *Circaetus cinerascens* | eB*,G* |  |
| 118 | ACCIPITRIFORMES | Accipitridae | Black-chested Snake-Eagle | *Circaetus pectoralis* | F*,eB*,G*,P* |  |
| 119 | PASSERIFORMES | Cisticolidae | Desert Cisticola | *Cisticola aridulus* | F*,eB*,G*,X* |  |
| 120 | PASSERIFORMES | Cisticolidae | Boran Cisticola | *Cisticola bodessa* | eB*,X* |  |
| 121 | PASSERIFORMES | Cisticolidae | Short-winged Cisticola | *Cisticola brachypterus* | eB*,G*,X*,M* |  |
| 122 | PASSERIFORMES | Cisticolidae | Pectoral-patch Cisticola | *Cisticola brunnescens* | F*,eB*,G*,X*,P* |  |
| 123 | PASSERIFORMES | Cisticolidae | Singing Cisticola | *Cisticola cantans* | F*,eB*,G*,X*,M* |  |
| 124 | PASSERIFORMES | Cisticolidae | Rattling Cisticola | *Cisticola chiniana* | F*,eB*,G*,X* |  |
| 125 | PASSERIFORMES | Cisticolidae | Ashy Cisticola | *Cisticola cinereolus* | F*,eB*,X* |  |
| 126 | PASSERIFORMES | Cisticolidae | Red-faced Cisticola | *Cisticola erythrops* | F*,eB*,G*,X*,M*,P* |  |
| 127 | PASSERIFORMES | Cisticolidae | Rufous-winged Cisticola | *Cisticola galactotes* | F*,eB*,G*,M*,P* | E^†^ |
| 128 | PASSERIFORMES | Cisticolidae | Zitting Cisticola | *Cisticola juncidis* | eB*,G*,X*,P* |  |
| 129 | PASSERIFORMES | Cisticolidae | Tiny Cisticola | *Cisticola nana* | F*,eB*,G*,X* |  |
| 130 | PASSERIFORMES | Cisticolidae | Croaking Cisticola | *Cisticola natalensis* | eB*,G*,X*,P* |  |
| 131 | PASSERIFORMES | Cisticolidae | Stout Cisticola | *Cisticola robustus* | F*,eB*,G*,P* |  |
| 132 | PASSERIFORMES | Cisticolidae | Red-pate Cisticola | *Cisticola ruficeps* | eB*,G*,X* |  |
| 133 | PASSERIFORMES | Cisticolidae | Foxy Cisticola | *Cisticola troglodytes* | F*,eB*,G*,X* |  |
| 134 | PASSERIFORMES | Estrildidae | Yellow-bellied Waxbill | *Coccopygia quartinia* | F*,eB*,G*,X*,P* |  |
| 135 | COLIIFORMES | Coliidae | Speckled MouseB* | *Colius striatus* | eB*,G*,X*,P* |  |
| 136 | COLUMBIFORMES | Columbidae | White-collared Pigeon | *Columba albitorques* | eB*,G*,X*,M*,P* | E^†^ |
| 137 | COLUMBIFORMES | Columbidae | African Olive Pigeon | *Columba arquatrix* | F*,eB*,G*,X*,M* |  |
| 138 | COLUMBIFORMES | Columbidae | Speckled Pigeon | *Columba guinea* | eB*,G*,X*,P* |  |
| 139 | COLUMBIFORMES | Columbidae | Lemon Dove | *Columba larvata* | F*,eB*,G*,X*,M*,P* |  |
| 140 | COLUMBIFORMES | Columbidae | Rock Pigeon | *Columba livia* | eB*,G*,M* |  |
| 141 | CORACIIFORMES | Coraciidae | Abyssinian Roller | *Coracias abyssinicus* | F*,eB*,G*,X*,P* |  |
| 142 | CORACIIFORMES | Coraciidae | Lilac-breasted Roller | *Coracias caudatus* | F*,eB*,G*,X*,M* |  |
| 143 | CORACIIFORMES | Coraciidae | Rufous-crowned Roller | *Coracias naevius* | F*,eB*,G*,M* |  |
| 144 | PASSERIFORMES | Campephagidae | Grey Cuckooshrike | *Coracina caesia* | F*,eB*,G*,M*,P* |  |
| 145 | PASSERIFORMES | Campephagidae | White-breasted Cuckooshrike | *Coracina pectoralis* | F*,eB*,G*,X* |  |
| 146 | PASSERIFORMES | Corvidae | Pied Crow | *Corvus albus* | F*,eB*,G*,X*,M*,P* |  |
| 147 | PASSERIFORMES | Corvidae | Cape Crow | *Corvus capensis* | F*,eB*,G*,X*,M*,P* |  |
| 148 | PASSERIFORMES | Corvidae | Thick-billed Raven | *Corvus crassirostris* | F*,eB*,G*,X*,M*,P* | E^†^ |
| 149 | PASSERIFORMES | Corvidae | Somali Crow | *Corvus edithae* | eB*,G*,X* |  |
| 150 | PASSERIFORMES | Corvidae | Fan-tailed Raven | *Corvus rhipidurus* | F*,eB*,G*,X*,M*,P* |  |
| 151 | PASSERIFORMES | Corvidae | Brown-necked Raven | *Corvus ruficollis* | eB*,G* |  |
| 152 | CUCULIFORMES | Musophagidae | White-bellied Go-away-bird | *Corythaixoides leucogaster* | eB*,G*,X*,M* |  |
| 153 | CUCULIFORMES | Musophagidae | Bare-faced Go-away-bird | *Corythaixoides personatus* | F*,eB*,G*,X*,M*,P* |  |
| 154 | CORACIIFORMES | Alcedinidae | Malachite Kingfisher | *Corythornis cristatus* | F*,eB*,G*,X*,P* |  |
| 155 | PASSERIFORMES | Muscicapidae | White-crowned Robin-Chat | *Cossypha albicapillus* | eB*,G*,X* |  |
| 156 | PASSERIFORMES | Muscicapidae | White-browed Robin-Chat | *Cossypha heuglini* | eB*,G*,X* |  |
| 157 | PASSERIFORMES | Muscicapidae | Snowy-crowned Robin-Chat | *Cossypha niveicapilla* | F*,eB*,G*,X* |  |
| 158 | PASSERIFORMES | Muscicapidae | Ruppell's Robin-Chat | *Cossypha semirufa* | F*,eB*,G*,X*,M*,P* |  |
| 159 | PASSERIFORMES | Sturnidae | Wattled Starling | *Creatophora cinerea* | F*,eB*,G*,X* |  |
| 160 | CUCULIFORMES | Musophagidae | Eastern Plantain-eater | *Crinifer zonurus* | F*,eB*,G*,X*,M*,P* |  |
| 161 | PASSERIFORMES | Fringillidae | Ankober Serin | *Crithagra ankoberensis* | eB*,G*,X* | E^†^ |
| 162 | PASSERIFORMES | Fringillidae | White-rumped Seedeater | *Crithagra leucopygia* | eB*,G*,P* |  |
| 163 | PASSERIFORMES | Fringillidae | Yellow-fronted Canary | *Crithagra mozambica* | F*,eB*,G*,X*,M*,P* |  |
| 164 | PASSERIFORMES | Fringillidae | Salvadori's Seedeater | *Crithagra xantholaema* | F*,eB*,G*,X* |  |
| 165 | PASSERIFORMES | Fringillidae | Yellow-rumped seedeater | *Crithagra xanthopygia* | F*,eB*,G*,X*,M*,P* | E^†^ |
| 166 | PASSERIFORMES | Fringillidae | African Citril | *Crithagra citrinelloides* | F*,eB*,G*,X* |  |
| 167 | PASSERIFORMES | Fringillidae | Northern Grosbeak-Canary | *Crithagra donaldsoni* | F*,eB*,G*,X* |  |
| 168 | PASSERIFORMES | Fringillidae | Yellow-throated seedeater | *Crithagra flavigula* | eB*,G*, | E^†^ |
| 169 | PASSERIFORMES | Fringillidae | Reichard's Seedeater | *Crithagra reichardi* | F*,eB*,G* |  |
| 170 | PASSERIFORMES | Fringillidae | Reichenow's Seedeater | *Crithagra reichenowi* | F*,eB*,G*,X*,M*,P* |  |
| 171 | PASSERIFORMES | Fringillidae | Streaky Seedeater | *Crithagra striolata* | F*,eB*,G*,X*,M*,P* |  |
| 172 | PASSERIFORMES | Fringillidae | Brown-rumped Seedeater | *Crithagra tristriata* | eB*,G* | E^†^ |
| 173 | PASSERIFORMES | Estrildidae | Abyssinian Crimson-wing | *Cryptospiza salvadorii* | F*,eB*,G*,X* |  |
| 174 | CHARADRIIFORMES | Glareolidae | Somali Courser | *Cursorius somalensis* | F*,eB*,G*,X*,M*,P* |  |
| 175 | ANSERIFORMES | Anatidae | Blue-winged Goose | *Cyanochen cyanoptera* | F*,eB*,G*,P*, | E^†^ |
| 176 | PASSERIFORMES | Nectariniidae | Olive Sunbird | *Cyanomitra olivacea* | eB*,G*,P* |  |
| 177 | APODIFORMES | Apodidae | African Palm-Swift | *Cypsiurus parvus* | eB*,G* |  |
| 178 | PICIFORMES | picidae | Brown-backed Woodpecker | *Dendrocopos obsoletus* | F*,eB*,G*,X*,M*,P* |  |
| 179 | ANSERIFORMES | Anatidae | White-faced Whistling-Duck | *Dendrocygna viduata* | F*,eB*,G*,X*,M*,P* |  |
| 180 | GALLIFORMES | Phasianidae | Crested Francolin | *Dendroperdix sephaena* | F*,eB*,G*,X*,P* |  |
| 181 | PICIFORMES | Picidae | Abyssinian Woodpecker | *Dendropicos abyssinicus* | F*,eB*,G*,X*,M* | E^†^ |
| 182 | PICIFORMES | Picidae | Cardinal Woodpecker | *Dendropicos fuscescens* | eB*,G*,M*,P* |  |
| 183 | PICIFORMES | Picidae | African Gray Woodpecker | *Dendropicos goertae* | F*,eB*,BIF,P* |  |
| 184 | PICIFORMES | Picidae | Bearded Woodpecker | *Dendropicos namaquus* | eB*,G*,X*,P* |  |
| 185 | PICIFORMES | Picidae | Eastern Grey Woodpecker | *Dendropicos spodocephalus* | F*,eB*,G*,X*,M*,P* |  |
| 186 | PASSERIFORMES | Dicruridae | Fork-tailed Drongo | *Dicrurus adsimilis* | eB*,G*,X*,M* |  |
| 187 | PASSERIFORMES | Ploceidae | White-headed Buffalo-Weaver | *Dinemellia dinemelli* | eB*,G* |  |
| 188 | CHARADRIIFORMES | Dromadidae | Crab-plover | *Dromas ardeola* | F*,eB*,G*,X*,P* |  |
| 189 | PASSERIFORMES | Malaconotidae | Northern Puffback | *Dryoscopus gambensis* | F*,eB*,G*,X* |  |
| 190 | PASSERIFORMES | Malaconotidae | Pringle's Puffback | *Dryoscopus pringlii* | F*,eB*,G* |  |
| 191 | PELECANIFORMES | Ardeidae | Western Reef-Heron | *Egretta gularis* | F*,eB*,G*,M*,P* |  |
| 192 | ACCIPITRIFORMES | Accipitridae | Black-shouldered Kite | *Elanus caeruleus* | F*,eB*,G*,X* |  |
| 193 | PASSERIFORMES | Emberizidae | Brown-rumped Bunting | *Emberiza affinis* | eB*,G* |  |
| 194 | PASSERIFORMES | Emberizidae | Golden-breasted Bunting | *Emberiza flaviventris* | F*,eB*,G*,X* |  |
| 195 | PASSERIFORMES | Emberizidae | Somali Bunting | *Emberiza poliopleura* | eB*,G* |  |
| 196 | PASSERIFORMES | Emberizidae | Striolated Bunting | *Emberiza striolata* | F*,eB*,G*,X*,P* |  |
| 197 | PASSERIFORMES | Emberizidae | Cinnamon-breasted Bunting | *Emberiza tahapisi* | F*,eB*,G*,X*,M* |  |
| 198 | PASSERIFORMES | Cisticolidae | Green-backed Eremomela | *Eremomela canescens* | F*,eB*,G*,X* |  |
| 199 | PASSERIFORMES | Cisticolidae | Yellow-vented Eremomela | *Eremomela flavicrissalis* | eB*,G*,X* |  |
| 200 | PASSERIFORMES | Cisticolidae | Yellow-bellied Eremomela | *Eremomela icteropygialis* | F*,eB*,G*,X*,M* |  |
| 201 | PASSERIFORMES | Alaudidae | Chestnut-backed Sparrow-Lark | *Eremopterix leucotis* | eB*,G* |  |
| 202 | PASSERIFORMES | Alaudidae | Black-crowned Sparrow-Lark | *Eremopterix nigriceps* | F*,eB*,G*,X* |  |
| 203 | PASSERIFORMES | Alaudidae | Chestnut-headed Sparrow-Lark | *Eremopterix signatus* | F*,eB*,G*,X*,P* |  |
| 204 | PASSERIFORMES | Estrildidae | Common Waxbill | *Estrilda astrild* | F*,eB*,G*,X* |  |
| 205 | PASSERIFORMES | Estrildidae | Black-cheeked Waxbill | *Estrilda charmosyna* | eB*,G*,M* |  |
| 206 | PASSERIFORMES | Estrildidae | Fawn-breasted Waxbill | *Estrilda paludicola* | F*,eB*,G*,X* |  |
| 207 | PASSERIFORMES | Estrildidae | Crimson-rumped Waxbill | *Estrilda rhodopyga* | eB*,G*,X* |  |
| 208 | PASSERIFORMES | Estrildidae | Black-rumped Waxbill | *Estrilda troglodytes* | F*,eB*,G* |  |
| 209 | PASSERIFORMES | Estrildidae | African Silverbill | *Euodice cantans* | eB*,G*,M*,P* |  |
| 210 | PASSERIFORMES | Ploceidae | Yellow-crowned Bishop | *Euplectes afer* | F*,eB*,G*,M* |  |
| 211 | PASSERIFORMES | Ploceidae | White-winged Widowbird | *Euplectes albonotatus* | F*,eB*,G*,M*,P* |  |
| 212 | PASSERIFORMES | Ploceidae | Red-collared Widowbird | *Euplectes ardens* | eB*,G*,M*,P* |  |
| 213 | PASSERIFORMES | Ploceidae | Fan-tailed Widowbird | *Euplectes axillaris* | F*,eB*,G*,M*,P* |  |
| 214 | PASSERIFORMES | Ploceidae | Yellow Bishop | *Euplectes capensis* | F*,eB*,G*,X*,P* |  |
| 215 | PASSERIFORMES | Ploceidae | Northern Red Bishop | *Euplectes franciscanus* | eB*,G*,P* |  |
| 216 | PASSERIFORMES | Ploceidae | Black Bishop | *Euplectes gierowii* | F*,eB*,G*,X*,M* |  |
| 217 | PASSERIFORMES | Ploceidae | Black-winged Red Bishop | *Euplectes hordeaceus* | eB*,G*,M*,P* |  |
| 218 | PASSERIFORMES | Ploceidae | Yellow-mantled Widowbird | *Euplectes macroura* | F*,eB*,G*,X* |  |
| 219 | OTIDIFORMES | Otididae | White-bellied Bustard | *Eupodotis senegalensis* | eB*,G*,M* |  |
| 220 | PASSERIFORMES | Laniidae | White-rumped Shrike | *Eurocephalus rueppelli* | F*,eB*,G* |  |
| 221 | FALCONIFORMES | Falconidae | Fox Kestrel | *Falco alopex* | F*,eB*,G*,M*,P* |  |
| 222 | FALCONIFORMES | Falconidae | Grey Kestrel | *Falco ardosiaceus* | F*,eB*,G* |  |
| 223 | FALCONIFORMES | Falconidae | Lanner Falcon | *Falco biarmicus* | eB*,G* |  |
| 224 | FALCONIFORMES | Falconidae | Red-necked Falcon | *Falco chicquera* | F*,eB*,G*,M*,P* |  |
| 225 | FALCONIFORMES | Falconidae | African Hobby | *Falco cuvierii* | eB*,G* |  |
| 226 | FALCONIFORMES | Falconidae | Barbary Falcon | *Falco pelegrinoides* | F*,eB*,G* |  |
| 227 | FALCONIFORMES | Falconidae | Peregrine Falcon | *Falco peregrinus* | F*,eB*,G*,X*,M*,P* |  |
| 228 | FALCONIFORMES | Falconidae | Common Kestrel | *Falco tinnunculus* | F*,eB*,G*,X*,M* |  |
| 229 | GRUIFORMES | Rallidae | Red-knobbed Coot | *Fulica cristata* | eB*,M*,P* |  |
| 230 | PASSERIFORMES | Alaudidae | Crested Lark | *Galerida cristata* | eB*,G*,X*,P* |  |
| 231 | PASSERIFORMES | Alaudidae | Thekla Lark | *Galerida theklae* | F*,eB*,G*,X*,P* |  |
| 232 | CHARADRIIFORMES | Scolopacidae | African Snipe | *Gallinago nigripennis* | F*,eB*,G* |  |
| 233 | GRUIFORMES | Rallidae | Common Moorhen | *Gallinula chloropus* | F*,eB*,G* |  |
| 234 | CHARADRIIFORMES | Glareolidae | Collared Pratincole | *Glareola pratincola* | F*,eB*,G*,X* |  |
| 235 | STRIGIFORMES | Strigidae | Pearl-spotted Owlet | *Glaucidium perlatum* | F*,eB*,G*,X*,P* |  |
| 236 | GRUIFORMES | Gruidae | Wattled Crane | *Grus carunculata* | F*,eB*,G*,X* |  |
| 237 | PASSERIFORMES | Passeridae | Bush Petronia | *Gymnoris dentata* | F*,eB*,G*,X*,M* |  |
| 238 | PASSERIFORMES | Passeridae | Yellow-spotted Petronia | *Gymnoris pyrgita* | F*,eB*,G*,P* |  |
| 239 | ACCIPITRIFORMES | Accipitridae | Lammergeier | *Gypaetus barbatus* | F*,eB*,G*,X*,P* |  |
| 240 | ACCIPITRIFORMES | Accipitridae | White-backed Vulture | *Gyps africanus* | F*,eB*,G*,X* |  |
| 241 | ACCIPITRIFORMES | Accipitridae | Rueppell's Griffon | *Gyps rueppelli* | eB*,G* |  |
| 242 | CORACIIFORMES | Alcedinidae | Brown-hooked Kingfish | *Halcyon albiventris* | F*,eB*,G*,X*,M*,P* |  |
| 243 | CORACIIFORMES | Alcedinidae | Striped Kingfisher | *Halcyon chelicuti* | F*,eB*,G*,X*,P* |  |
| 244 | ACCIPITRIFORMES | Accipitridae | African Fish-Eagle | *Haliaeetus vocifer* | F*,eB*,G*,X*,P* |  |
| 245 | PASSERIFORMES | Nectariniidae | Collared Sunbird | *Hedydipna collaris* | F*,eB*,G* |  |
| 246 | PASSERIFORMES | Nectariniidae | Nile Valley Sunbird | *Hedydipna metallica* | F*,eB*,G*,X* |  |
| 247 | PASSERIFORMES | Alaudidae | Archer's Lark | *Heteromirafra archeri* | F*,G*,X* |  |
| 248 | PASSERIFORMES | Alaudidae | Sidamo Lark | *Heteromirafra sidamoensis* | F*,eB*,G* |  |
| 249 | ACCIPITRIFORMES | Accipitridae | Ayres's Hawk-Eagle | *Hieraaetus ayresii* | F*,G*,P* |  |
| 250 | FALCONIFORMES | Accipitridae | African Hawk Eagle | *Hieraaetus spilogaster* | F*,eB*,G*,X* |  |
| 251 | CHARADRIIFORMES | Recurvirostridae | Black-winged Stilt | *Himantopus himantopus* | F*,eB*,G*,X*,M*,P* |  |
| 252 | PASSERIFORMES | Hirundinidae | Ethiopian Swallow | *Hirundo aethiopica* | F*,eB*,G*,P* |  |
| 253 | PASSERIFORMES | Hirundinidae | Red-chested Swallow | *Hirundo lucida* | eB*,G*,X*,P* |  |
| 254 | PASSERIFORMES | Hirundinidae | White-tailed Swallow | *Hirundo megaensis* | F*,eB*,G*,P* | E^†^ |
| 255 | PASSERIFORMES | Hirundinidae | Wire-tailed Swallow | *Hirundo smithii* | eB*,G* |  |
| 256 | PASSERIFORMES | Hyliotidae | Yellow-bellied Hyliota | *Hyliota flavigaster* | eB*,G* |  |
| 257 | CHARADRIIFORMES | Laridae | Sooty Gull | *Ichthyaetus hemprichii* | eB*,G* |  |
| 258 | CHARADRIIFORMES | Laridae | White-eyed Gull | *Ichthyaetus leucophthalmus* | F*,eB*,G*,X*,P* |  |
| 259 | PASSERIFORMES | Acrocephalidae | Eastern Olivaceous Warbler | *Iduna pallida* | F*,eB*,G*,X*,M*,P* |  |
| 260 | PICIFORMES | Indicatoridae | Lesser Honeyguide | *Indicator minor* | F*,eB*,G*,X* |  |
| 261 | PICIFORMES | Indicatoridae | Scaly-throated Honeyguide | *Indicator variegatus* | F*,eB*,G*,X*,M* |  |
| 262 | PICIFORMES | Picidae | Red-throated Wryneck | *Jynx ruficollis* | eB*,G*,P* |  |
| 263 | ACCIPITRIFORMES | Accipitridae | Lizard Buzzard | *Kaupifalco monogrammicus* | F*,eB*,G*,X* |  |
| 264 | PASSERIFORMES | Estrildidae | Black-faced Firefinch | *Lagonosticta larvata* | eB*,G* |  |
| 265 | PASSERIFORMES | Estrildidae | Jameson's Firefinch | *Lagonosticta rhodopareia* | F*,eB*,G*,X*,P* |  |
| 266 | PASSERIFORMES | Estrildidae | African Firefinch | *Lagonosticta rubricata* | F*,eB*,G* |  |
| 267 | PASSERIFORMES | Estrildidae | Bar-breasted Firefinch | *Lagonosticta rufopicta* | F*,eB*,G*,X*,M*,P* |  |
| 268 | PASSERIFORMES | Estrildidae | Red-billed Firefinch | *Lagonosticta senegala* | F*,eB*,G*,X* |  |
| 269 | PASSERIFORMES | Sturnidae | Greater Blue-eared Starling | *Lamprotornis chalybaeus* | P*,F*,eB*,G*,X* |  |
| 270 | PASSERIFORMES | Sturnidae | Lesser Blue-eared Starling | *Lamprotornis chloropterus* | eB*,G*,X*,M*,P* |  |
| 271 | PASSERIFORMES | Sturnidae | Rueppell's Starling | *Lamprotornis purpuroptera* | F*,eB*,G*,X* |  |
| 272 | PASSERIFORMES | Sturnidae | Golden-breasted Starling | *Lamprotornis regius* | F*,eB*,G*,X* |  |
| 273 | PASSERIFORMES | Sturnidae | Shelley's Starling | *Lamprotornis shelleyi* | eB*,G*,eB*,G*,X* |  |
| 274 | PASSERIFORMES | Sturnidae | Splendid Starling | *Lamprotornis splendidus* | F*,eB*,G*,X*,M* |  |
| 275 | PASSERIFORMES | Sturnidae | Superb Starling | *Lamprotornis superbus* | eB*,G*,X*,P* |  |
| 276 | PASSERIFORMES | Sturnidae | White-crowned Starling | *Lamprotornis albicapillus* | eB*,G*,X* |  |
| 277 | PASSERIFORMES | Malaconotidae | Ethiopian Boubou | *Laniarius aethiopicus* | eB*,G*,X* |  |
| 278 | PASSERIFORMES | Malaconotidae | Black-headed Gonolek | *Laniarius erythrogaster* | F*,eB*,G*,X* |  |
| 279 | PASSERIFORMES | Malaconotidae | Slate-colored Boubou | *Laniarius funebris* | eB*,G*,M*,P* |  |
| 280 | PASSERIFORMES | Malaconotidae | Red-naped Bushshrike | *Laniarius ruficeps* | eB*,G*,X* |  |
| 281 | PASSERIFORMES | Laniidae | Southern Fiscal | *Lanius collaris* | eB*,G*,X* |  |
| 282 | PASSERIFORMES | Laniidae | Taita Fiscal | *Lanius dorsalis* | eB*,G* |  |
| 283 | PASSERIFORMES | Laniidae | Northern Shrike | *Lanius excubitor* | F*,eB*,G*,X* |  |
| 284 | PASSERIFORMES | Laniidae | Southern Gray Shrike | *Lanius meridionalis* | eB*,G* |  |
| 285 | PASSERIFORMES | Laniidae | Somali Fiscal | *Lanius somalicus* | eB*,G* |  |
| 286 | OTIDIFORMES | Otididae | Hartlaub's Bustard | *Lissotis hartlaubii* | F*,eB*,G*,X* |  |
| 287 | OTIDIFORMES | Otididae | Black-bellied Bustard | *Lissotis melanogaster* | F*,eB*,G*,X*,P* |  |
| 288 | PASSERIFORMES | Estrildidae | Black-and-white Mannikin | *Lonchura bicolor* | eB*,M* |  |
| 289 | PASSERIFORMES | Estrildidae | Bronze Mannikin | *Lonchura cucullata* | F*,eB*,G*,X*,M*,P* |  |
| 290 | PASSERIFORMES | Estrildidae | Magpie Mannikin | *Lonchura fringilloides* | eB*,M* |  |
| 291 | ACCIPITRIFORMES | Accipitridae | Long-crested Eagle | *Lophaetus occipitalis* | F*,eB*,P* |  |
| 292 | BUCEROTIFORMES | Bucerotidae | Crowned Hornbill | *Lophoceros alboterminatus* | F*,eB* |  |
| 293 | BUCEROTIFORMES | Bucerotidae | Hemprich's Hornbill | *Lophoceros hemprichii* | F*,eB*,G*,X* |  |
| 294 | BUCEROTIFORMES | Bucerotidae | African Gray Hornbill | *Lophoceros nasutus* | F*,eB*,G*,X*,M*,P* |  |
| 295 | OTIDIFORMES | Otididae | Buff-crested Bustard | *Lophotis gindiana* | F*,eB*,G*,X*,P* |  |
| 296 | PICIFORMES | Lybiidae | Double-toothed Barbet | *Lybius bidentatus* | F*,eB*,G*,X*,M*,P* |  |
| 297 | PICIFORMES | Lybiidae | Black-billed Barbet | *Lybius guifsobalito* | eB*,G* |  |
| 298 | PICIFORMES | Lybiidae | Banded Barbet | *Lybius undatus* | F*,eB*,G*,X*,M*,P* | E^†^ |
| 299 | ACCIPITRIFORMES | Accipitridae | Bat Hawk | *Macheiramphus alcinus* | F*,eB*,G*,X*,P* |  |
| 300 | PASSERIFORMES | Motacillidae | Abyssinian Longclaw | *Macronyx flavicollis* | F*,eB*,G*,X* | E^†^ |
| 301 | PASSERIFORMES | Malaconotidae | Gray-headed Bushshrike | *Malaconotus blanchoti* | F*,eB*,G*,X*,P* |  |
| 302 | PASSERIFORMES | Estrildidae | Green-backed Twinspot | *Mandingoa nitidula* | F* |  |
| 303 | CORACIIFORMES | Alcedinidae | Giant Kingfisher | *Megaceryle maxima* | eB*,G*,X*,M*,P* |  |
| 304 | PASSERIFORMES | Muscicapidae | Abyssinian Slaty-Flycatcher | *Melaenornis chocolatinus* | F*,eB*,G*,X*,M*,P* | E^†^ |
| 305 | PASSERIFORMES | Muscicapidae | Northern Black-Flycatcher | *Melaenornis edolioides* | eB* |  |
| 306 | PASSERIFORMES | Muscicapidae | Southern Black Flycatcher | *Melaenornis pammelaina* | G*,X* |  |
| 307 | ACCIPITRIFORMES | Paridae | White-backed Black Tit | *Melaniparus leuconotus* | G*,X* |  |
| 308 | ACCIPITRIFORMES | Paridae | Acacia Tit | *Melaniparus thruppi* | F*,eB*,G*,M* |  |
| 309 | ACCIPITRIFORMES | Accipitridae | Dark Chanting-Goshawk | *Melierax metabates* | eB*,G*,X* |  |
| 310 | ACCIPITRIFORMES | Accipitridae | Eastern Chanting-Goshawk | *Melierax poliopterus* | eB*,G*,X* |  |
| 311 | PASSERIFORMES | Macrosphenidae | Moustached Grass-Warbler | *Melocichla mentalis* | F*,eB*,G*,X*,P* |  |
| 312 | CORACIIFORMES | Meropidae | Blue-breasted Bee-eater | *Merops variegatus* | eB*,G*,P* |  |
| 313 | CORACIIFORMES | Meropidae | Red-throated Bee-eater | *Merops bulocki* | F*,eB*,G*,X*,P* |  |
| 314 | CORACIIFORMES | Meropidae | Northern Carmine Bee-eater | *Merops nubicus* | F*,eB*,G*,X*,P* |  |
| 315 | CORACIIFORMES | Meropidae | Little Bee-eater | *Merops pusillus* | F*,eB*,G*,P* |  |
| 316 | PELECANIFORMES | Ardeidae | Intermediate Egret | *Egretta intermedia* | F*,eB*,G*,M*,P* |  |
| 317 | ACCIPITRIFORMES | Accipitridae | Gabar Goshawk | *Micronisus gabar* | F*,eB*,G* |  |
| 318 | CHARADRIIFORMES | Jacanidae | Lesser Jacana | *Microparra capensis* | F*,eB*,G*,M*,P* |  |
| 319 | ACCIPITRIFORMES | Accipitridae | Black Kite | *Milvus migrans* | eB*,G* |  |
| 320 | PASSERIFORMES | Alaudidae | Singing Bushlark | *Mirafra cantillans* | eB*,G* |  |
| 321 | PASSERIFORMES | Alaudidae | Collared Lark | *Mirafra collaris* | eB*,G*,X*,P* |  |
| 322 | PASSERIFORMES | Alaudidae | Red-winged Lark | *Mirafra hypermetra* | eB*,G*,X*,P* |  |
| 323 | PASSERIFORMES | Alaudidae | Flappet Lark | *Mirafra rufocinnamomea* | F*,eB*,G*,M* |  |
| 324 | PASSERIFORMES | Muscicapidae | Little Rock-Thrush | *Monticola rufocinereus* | F*,eB*,G*,X*,P* |  |
| 325 | PASSERIFORMES | Motacillidae | African Pied Wagtail | *Motacilla aguimp* | F*,eB*,G*,X*,P* |  |
| 326 | PASSERIFORMES | Motacillidae | Mountain Wagtail | *Motacilla clara* | F*,eB*,G*,X*,M*,P* |  |
| 327 | PASSERIFORMES | Muscicapidae | Dusky-brown Flycatcher | *Muscicapa adusta* | eB*,G* |  |
| 328 | PASSERIFORMES | Muscicapidae | Gambaga Flycatcher | *Muscicapa gambagae* | eB*,G*,P* |  |
| 329 | CICONIIFORMES | Ciconiidae | Yellow-billed Stork | *Mycteria ibis* | eB*,G* |  |
| 330 | PASSERIFORMES | Muscicapidae | Gray Tit-Flycatcher | *Myioparus plumbeus* | eB*,G* |  |
| 331 | PASSERIFORMES | Muscicapidae | White-fronted Black-Chat | *Myrmecocichla albifrons* | F*,eB*,G*,X* |  |
| 332 | PASSERIFORMES | Muscicapidae | Rueppell's Chat | *Myrmecocichla melaena* | F*,eB*,G*,X*,P* | E^†^ |
| 333 | ACCIPITRIFORMES | Accipitridae | Hooded Vulture | *Necrosyrtes monachus* | eB*,G*,P* |  |
| 334 | PASSERIFORMES | Nectariniidae | Malachite Sunbird | *Nectarinia famosa* | eB*,G*,X*,M*,P* |  |
| 335 | PASSERIFORMES | Nectariniidae | Tacazze Sunbird | *Nectarinia tacazze* | eB*,G* |  |
| 336 | ACCIPITRIFORMES | Accipitridae | Egyptian Vulture | *Neophron percnopterus* | eB*,G* |  |
| 337 | OTIDIFORMES | Otididae | Heuglin's Bustard | *Neotis heuglinii* | eB*,G*,X*,M* |  |
| 338 | PASSERIFORMES | Malaconotidae | Brubru | *Nilaus afer* | F*,eB*,G*,X*,P* |  |
| 339 | GALLIFORMES | Numididae | Helmeted Guineafowl | *Numida meleagris* | eB*,G*,X* |  |
| 340 | PELECANIFORMES | Ardeidae | Black-crowned Night-Heron | *Nycticorax nycticorax* | F*,eB*,G* |  |
| 341 | PASSERIFORMES | Estrildidae | Gray-headed Silverbill | *Odontospiza griseicapilla* | F*,eB*,G*,P* |  |
| 342 | COLUMBIFORMES | Columbidae | Namaqua Dove | *Oena capensis* | F*,eB*,G*,X* |  |
| 343 | PASSERIFORMES | Muscicapidae | Red-breasted Wheatear | *Oenanthe bottae* | eB*,G* |  |
| 344 | PASSERIFORMES | Muscicapidae | White-crowned Wheatear | *Oenanthe leucopyga* | F*,eB*,G*,X* |  |
| 345 | PASSERIFORMES | Muscicapidae | Abyssinian Wheatear | *Oenanthe lugubris* | eB*,G* | E^†^ |
| 346 | PASSERIFORMES | Muscicapidae | Somali Wheatear | *Oenanthe phillipsi* | F*,eB*,G* | E^†^ |
| 347 | PASSERIFORMES | Muscicapidae | Familiar Chat | *Oenanthe familiaris* | F*,eB*,G*,X* |  |
| 348 | PASSERIFORMES | Muscicapidae | Blackstart | *Oenanthe melanura* | F*,eB*,G*,X* |  |
| 349 | PASSERIFORMES | Muscicapidae | Brown-tailed Rock Chat | *Oenanthe scotocerca* | F*,eB*,G*,X*,M* |  |
| 350 | PASSERIFORMES | Sturnidae | White-billed Starling | *Onychognathus albirostris* | eB*,G* | E^†^ |
| 351 | PASSERIFORMES | Sturnidae | Somali Starling | *Onychognathus blythii* | F*,eB*,G*,P* | E^†^ |
| 352 | PASSERIFORMES | Sturnidae | Red-winged Starling | *Onychognathus morio* | F*,eB*,G*,X* |  |
| 353 | PASSERIFORMES | Sturnidae | Bristle-crowned Starling | *Onychognathus salvadorii* | F*,eB*,G*,X* |  |
| 354 | PASSERIFORMES | Sturnidae | Slender-billed Starling | *Onychognathus tenuirostris* | F*,eB*,G*,X*,M*,P* |  |
| 355 | PASSERIFORMES | Oriolidae | African Black-headed Oriole | *Oriolus larvatus* | F*,eB*,G*,X*,P* |  |
| 356 | PASSERIFORMES | Oriolidae | Ethiopian Black-headed Oriole | *Oriolus monacha* | eB*,G* | E^†^ |
| 357 | PASSERIFORMES | Estrildidae | African Quailfinch | *Ortygospiza fuscocrissa* | F*,eB*G*,X* |  |
| 358 | STRIGIFORMES | Strigidae | African Scops-Owl | *Otus senegalensis* | F*,eB*,G* |  |
| 359 | ANSERIFORMES | Anatidae | Maccoa Duck | *Oxyura maccoa* | eB*,G*,P* |  |
| 360 | ACCIPITRIFORMES | Pandionidae | Western Osprey | *Pandion haliaetus* | F*,eB*,G*,X*,M*,P* |  |
| 361 | PASSERIFORMES | Sylviidae | Abyssinian Catbird | *Parophasma galinieri* | eB*,G* | E^†^ |
| 362 | ACCIPITRIFORMES | Paridae | White-shouldered Black Tit | *Parus guineensis* | F*,eB*,M*,P* |  |
| 363 | ACCIPITRIFORMES | Paridae | White-backed Black Tit | *Parus leuconotus* | F*,eB* | E^†^ |
| 364 | ACCIPITRIFORMES | Paridae | Somali Tit | *Parus thruppi* | eB*,G* |  |
| 365 | PASSERIFORMES | Passeridae | Somali Sparrow | *Passer castanopterus* | F*,eB* |  |
| 366 | PASSERIFORMES | Passeridae | House Sparrow | *Passer domesticus* | F*,eB*,G*,X*,P* |  |
| 367 | PASSERIFORMES | Passeridae | Chestnut Sparrow | *Passer eminibey* | eB*,G* |  |
| 368 | PASSERIFORMES | Passeridae | Parrot-billed Sparrow | *Passer gongonensis* | F*,eB*,G*,X*,P* |  |
| 369 | PASSERIFORMES | Passeridae | Northern Gray-headed Sparrow | *Passer griseus* | F*,eB*,G*,X* |  |
| 370 | PASSERIFORMES | Passeridae | Shelley's Sparrow | *Passer shelleyi* | F*,eB*,G*,X*,M*,P* |  |
| 371 | PASSERIFORMES | Passeridae | Swainson's Sparrow | *Passer swainsonii* | F*,eB*,G*,P* |  |
| 372 | PELECANIFORMES | Pelecanidae | Pink-backed Pelican | *Pelecanus rufescens* | F*,eB*,G* |  |
| 373 | GALLIFORMES | Phasianidae | Coqui Francolin | *Peliperdix coqui* | eB*,G*,M*,P* |  |
| 374 | SULIFORMES | Phalacrocoracidae | Long-tailed Cormorant | *Phalacrocorax africanus* | eB*,G*,P* |  |
| 375 | SULIFORMES | Phalacrocoracidae | Great Cormorant | *Phalacrocorax carbo* | eB*,G*,X* |  |
| 376 | BUCEROTIFORMES | Phoeniculidae | Green Woodhoopoe | *Phoeniculus purpureus* | F*,eB*,G*,X*,P* |  |
| 377 | BUCEROTIFORMES | Phoeniculidae | Black-billed Woodhoopoe | *Phoeniculus somaliensis* | F*,eB*,G*,X*,M*,P* |  |
| 378 | PASSERIFORMES | Pycnonotidae | Northern Brownbul | *Phyllastrephus strepitans* | F*,eB*,G*,X*,M*,P* |  |
| 379 | PASSERIFORMES | Cisticolidae | Buff-bellied Warbler | *Phyllolais pulchella* | F*,eB*,G*,X*,M*,P* |  |
| 380 | PASSERIFORMES | Phylloscopidae | Brown Woodland-Warbler | *Phylloscopus umbrovirens* | F*,eB*,X*,M*,P* |  |
| 381 | PASSERIFORMES | Muscicapidae | Moorland Chat | *Pinarochroa sordida* | eB*,G* |  |
| 382 | PELECANIFORMES | Threskiornithidae | Eurasian Spoonbill | *Platalea leucorodia* | F*,eB*,G*,M*,P* |  |
| 383 | PASSERIFORMES | Platysteiridae | Brown-throated Wattle-eye | *Platysteira cyanea* | F*,Bird,G*,P* |  |
| 384 | ANSERIFORMES | Anatidae | Spur-winged Goose | *Plectropterus gambensis* | eB*,G*,X*,M* |  |
| 385 | PASSERIFORMES | Passeridae | White-browed Sparrow-Weaver | *Plocepasser mahali* | F*,eB*,G*,X* |  |
| 386 | PASSERIFORMES | Passeridae | Chestnut-crowned Sparrow-Weaver | *Plocepasser superciliosus* | F*,eB*,G*,X*,M*,P* |  |
| 387 | PASSERIFORMES | Ploceidae | Baglafecht Weaver | *Ploceus baglafecht* | eB*,G* |  |
| 388 | PASSERIFORMES | Ploceidae | Golden Palm Weaver | *Ploceus bojeri* | eB*,G*,X*,M*,P* |  |
| 389 | PASSERIFORMES | Ploceidae | Village Weaver | *Ploceus cucullatus* | F*,eB*,G*,X* |  |
| 390 | PASSERIFORMES | Ploceidae | Juba Weaver | *Ploceus dichrocephalus* | eB*,G*,X*,P* |  |
| 391 | PASSERIFORMES | Ploceidae | Rueppell's Weaver | *Ploceus galbula* | F*,eB*,G*,X*,M*,P* |  |
| 392 | PASSERIFORMES | Ploceidae | Lesser Masked-Weaver | *Ploceus intermedius* | F*,eB*,G*,X*,P* |  |
| 393 | PASSERIFORMES | Ploceidae | Little Weaver | *Ploceus luteolus* | eB*,G*,P* |  |
| 394 | PASSERIFORMES | Ploceidae | Black-headed Weaver | *Ploceus melanocephalus* | eB*,G* |  |
| 395 | PASSERIFORMES | Ploceidae | Black-necked Weaver | *Ploceus nigricollis* | F*,eB*,G*,X*,M*,P* |  |
| 396 | PASSERIFORMES | Ploceidae | Spectacled Weaver | *Ploceus ocularis* | F*,eB*,G*,X*,M* |  |
| 397 | PASSERIFORMES | Ploceidae | Chestnut Weaver | *Ploceus rubiginosus* | eB*,G*,X*,M*,P* |  |
| 398 | PASSERIFORMES | Ploceidae | Speke's Weaver | *Ploceus spekei* | F*,eB*,G*,X* |  |
| 399 | PASSERIFORMES | Ploceidae | Northern Masked-Weaver | *Ploceus taeniopterus* | eB*,G*,X* |  |
| 400 | PASSERIFORMES | Ploceidae | Vitelline Masked-Weaver | *Ploceus vitellinus* | eB*,G* |  |
| 401 | PASSERIFORMES | Ploceidae | Compact Weaver | *Ploceus superciliosus* | eB*,G* |  |
| 402 | PODICIPEDIFORMES | Podicipedidae | Great Crested Grebe | *Podiceps cristatus* | F*,eB*,G*,X*,P* |  |
| 403 | PASSERIFORMES | Sturnidae | Sharpe's Starling | *Poeoptera sharpii* | F*,eB*,G*,X*,M*,P* | E^†^ |
| 404 | PICIFORMES | Lybiidae | Yellow-fronted Tinkerbird | *Pogoniulus chrysoconus* | F*,eB*,G*,X*,P* |  |
| 405 | PICIFORMES | Lybiidae | Red-fronted Tinkerbird | *Pogoniulus pusillus* | F*,eB*,G*,X*,M*,P* |  |
| 406 | PSITTACIFORMES | Psittacidae | Yellow-fronted Parrot | *Poicephalus flavifrons* | F*,eB*,G*,X* | E^†^ |
| 407 | PSITTACIFORMES | Psittacidae | Red-bellied Parrot | *Poicephalus rufiventris* | F*,eB*,G* |  |
| 408 | ACCIPITRIFORMES | Pandionidae | Martial Eagle | *Polemaetus bellicosus* | F*,eB*,G*,X*,M* |  |
| 409 | FALCONIFORMES | Falconidae | Pygmy Falcon | *Polihierax semitorquatus* | eB*,G* |  |
| 410 | GRUIFORMES | Rallidae | Purple Swamphen | *Porphyrio porphyrio* | eB*,G* |  |
| 411 | PASSERIFORMES | Cisticolidae | Graceful Prinia | *Prinia gracilis* | F*,eB*,G*,X* |  |
| 412 | PASSERIFORMES | Cisticolidae | Pale Prinia | *Prinia somalica* | eB*,G*,X*,M*,P* |  |
| 413 | PASSERIFORMES | Cisticolidae | Tawny-flanked Prinia | *Prinia subflava* | F*,eB*,G*,X* |  |
| 414 | PASSERIFORMES | Prionopidae | White Helmetshrike | *Prionops plumatus* | eB*,G*,P* |  |
| 415 | PICIFORMES | Indicatoridae | Brown-backed/Wahlberg's Honeyguide | *Prodotiscus regulus* | F*,eB*,G* |  |
| 416 | PASSERIFORMES | Hirundinidae | Grey-rumped Swallow | *Pseudhirundo griseopyga* | F*,G*,X*,M* ,P* |  |
| 417 | PASSERIFORMES | Timaliidae | African Hill Babbler | *Pseudoalcippe abyssinica* | eB*,G*,X* |  |
| 418 | PASSERIFORMES | Passeridae | Gray-cpped Social-Weaver | *Pseudonigrita arnaudi* | F*,eB*,G*,X* |  |
| 419 | PASSERIFORMES | Passeridae | Black-capped Social-Weaver | *Pseudonigrita cabanisi* | eB*,G*,P* |  |
| 420 | PSITTACIFORMES | Psittaculidae | Rose-ringed Parakeet | *Psittacula krameri* | F*,eB*,X*,P*,G* |  |
| 421 | GALLIFORMES | Phasianidae | Chestnut-naped Francolin | *Pternistis castaneicollis* | F*,eB*,X*,P*,G*,M* | E^†^ |
| 422 | GALLIFORMES | Phasianidae | Erckel's Francolin | *Pternistis erckelii* | G*,F*,eB*,X* |  |
| 423 | GALLIFORMES | Phasianidae | Harwood's Francolin | *Pternistis harwoodi* | eB*,G*,X* | E^†^ |
| 424 | GALLIFORMES | Phasianidae | Yellow-necked Spurfowl | *Pternistis leucoscepus* | F*,eB*,X*,G* |  |
| 425 | GALLIFORMES | Phasianidae | Scaly Francolin | *Pternistis squamatus* | F*,eB*,G*,X* |  |
| 426 | PTEROCLIFORMES | Pteroclidae | Black-faced Sandgrouse | *Pterocles decoratus* | F*,eB*,G* |  |
| 427 | PTEROCLIFORMES | Pteroclidae | Lichtenstein's Sandgrouse | *Pterocles lichtensteinii* | F*,eB*,G*,X*,M* |  |
| 428 | PTEROCLIFORMES | Pteroclidae | Four-banded Sandgrouse | *Pterocles quadricinctus* | eB*,G*,X* |  |
| 429 | STRIGIFORMES | Strigidae | Northern White-faced Owl | *Ptilopsis leucotis* | eB*,G*,X*,P* |  |
| 430 | PASSERIFORMES | Hirundinidae | Rock Martin | *Ptyonoprogne fuligula* | F*,eB*,G*,X*,M*,P* |  |
| 431 | PASSERIFORMES | Pycnonotidae | Common Bulbul | *Pycnonotus barbatus* | F*,eB*,G*,X* |  |
| 432 | PASSERIFORMES | Corvidae | Red-billed Chough | *Pyrrhocorax pyrrhocorax* | eB*,G* |  |
| 433 | PASSERIFORMES | Estrildidae | Orange-winged Pytilia | *Pytilia afra* | F*,eB*,G*,P* |  |
| 434 | PASSERIFORMES | Estrildidae | Red-billed Pytilia | *Pytilia lineata* | F*,eB*,G*,X* |  |
| 435 | PASSERIFORMES | Estrildidae | Green-winged Pytilia | *Pytilia melba* | F*,eB*,G*,M* |  |
| 436 | PASSERIFORMES | Ploceidae | Red-billed Quelea | *Quelea quelea* | F*,eB*,G*,X* |  |
| 437 | BUCEROTIFORMES | Phoeniculidae | Black Scimitar-bill | *Rhinopomastus aterrimus* | F*,eB*,G*,X* |  |
| 438 | BUCEROTIFORMES | Phoeniculidae | Abyssinian Scimitar-bill | *Rhinopomastus minor* | F*,eB*,G*,X* |  |
| 439 | CHARADRIIFORMES | Glareolidae | Three-banded Courser | *Rhinoptilus cinctus* | F*,eB*,G*,X*,P* |  |
| 440 | PASSERIFORMES | Hirundinidae | Brown-throated Martin | *Riparia paludicola* | F*,eB*,G*,X*,M*,P* |  |
| 441 | PASSERIFORMES | Hirundinidae | Brown-throated/Plain Martin | *Rougetius rougetii* | F*,eB*,G*,M*,P* | E^†^ |
| 442 | PASSERIFORMES | Muscicapidae | African Stonechat | *Saxicola torquatus* | F*,eB*,X*,G*,X* |  |
| 443 | GALLIFORMES | Phasianidae | Moorland Francolin | *Scleroptila psilolaema* | F*,eB*,G*,X*,M*,P* |  |
| 444 | PELECANIFORMES | Scopidae | Hamerkop | *Scopus umbretta* | F*,eB*,G*,M* |  |
| 445 | PASSERIFORMES | Fringillidae | White-bellied Canary | *Serinus dorsostriatus* | F*,eB*,G*,X* |  |
| 446 | PASSERIFORMES | Fringillidae | Yellow-crowned Canary | *Serinus flavivertex* | eB*,G*,X*,M*,P* |  |
| 447 | PASSERIFORMES | Fringillidae | Ethiopian Siskin | *Serinus nigriceps* | F*,eB*,G*,X* | E^†^ |
| 448 | PASSERIFORMES | Sturnidae | Magpie Starling | *Speculipastor bicolor* | F*,eB*,G*,P* |  |
| 449 | COLUMBIFORMES | Columbidae | Laughing Dove | *Spilopelia senegalensis* | F*,eB*,G*,X* |  |
| 450 | PASSERIFORMES | Alaudidae | Short-tailed Lark | *Spizocorys fremantlii* | F*,eB*,G*,X* |  |
| 451 | PASSERIFORMES | Ploceidae | Speckle-fronted Weaver | *Sporopipes frontalis* | F*,eB*,G*,X* |  |
| 452 | ACCIPITRIFORMES | Accipitridae | Crowned Hawk-Eagle | *Stephanoaetus coronatus* | eB*,G* |  |
| 453 | CHARADRIIFORMES | Laridae | Saunders's Tern | *Sternula saundersi* | eB*,G*,X*,P* |  |
| 454 | COLUMBIFORMES | Columbidae | Ring-necked Dove | *Streptopelia capicola* | F*,eB*,G*,X* |  |
| 455 | COLUMBIFORMES | Columbidae | Mourning Collared-Dove | *Streptopelia decipiens* | F*,eB*,G*,X*,P* |  |
| 456 | COLUMBIFORMES | Columbidae | Dusky Turtle-Dove | *Streptopelia lugens* | eB*,G*,X* |  |
| 457 | COLUMBIFORMES | Columbidae | White-winged Collared-Dove | *Streptopelia reichenowi* | eB*,G*,P* |  |
| 458 | COLUMBIFORMES | Columbidae | African Collared-Dove | *Streptopelia roseogrisea* | F*,eB*,G*,X*,M*,P* |  |
| 459 | COLUMBIFORMES | Columbidae | Red-eyed Dove | *Streptopelia semitorquata* | F*,eB*,G*,X* |  |
| 460 | COLUMBIFORMES | Columbidae | Vinaceous Dove | *Streptopelia vinacea* | F*,eB*,G*,X* |  |
| 461 | STRIGIFORMES | Strigidae | African Wood-Owl | *Strix woodfordii* | eB*,G* |  |
| 462 | STRUTHIONIFORMES | Struthionidae | Common Ostrich | *Struthio camelus* | eB*,X* |  |
| 463 | STRUTHIONIFORMES | Struthionidae | Common Ostrich | *Struthio molybdophanes* | eB*,G* |  |
| 464 | STRUTHIONIFORMES | Struthionidae | Common Ostrich | *Sula leucogaster* | F*,eB*,G*,X* |  |
| 465 | PASSERIFORMES | Sylviidae | Banded Parisoma | *Sylvia boehmi* | eB*,G* |  |
| 466 | PASSERIFORMES | Sylviidae | Arabian Warbler | *Sylvia leucomelaena* | F*,eB*,G*,X* |  |
| 467 | PASSERIFORMES | Sylviidae | Brown Parisoma | *Sylvia lugens* | F*,eB*,G*,X*,M* |  |
| 468 | PASSERIFORMES | Macrosphenidae | Northern Crombec | *Sylvietta brachyura* | F*,eB*,G*,X* |  |
| 469 | PASSERIFORMES | Macrosphenidae | Somali Crombec | *Sylvietta isabellina* | eB* |  |
| 470 | PASSERIFORMES | Macrosphenidae | Short-billed Crombec | *Sylvietta philippae* | F*,eB*,G*,X*,M* | E^†^ |
| 471 | PASSERIFORMES | Macrosphenidae | Red-faced Crombec | *Sylvietta whytii* | F*,eB*,G*,X*,M* |  |
| 472 | PODICIPEDIFORMES | Podicipedidae | Little Grebe | *Tachybaptus ruficollis* | F*,eB*,G*,X* |  |
| 473 | ANSERIFORMES | Anatidae | Ruddy Shelduck | *Tadorna ferruginea* | F*,eB*,G*,X*,M*,P* |  |
| 474 | CUCULIFORMES | Musophagidae | White-cheeked Turaco | *Tauraco leucotis* | F*,eB*,G*,X* |  |
| 475 | CUCULIFORMES | Musophagidae | Prince Ruspoli's Turaco | *Tauraco ruspolii* | F*,eB*,G*,X* | E^†^ |
| 476 | PASSERIFORMES | Malaconotidae | Three-streaked Tchagra | *Tchagra jamesi* | F*,eB*,G*,X*,P* |  |
| 477 | PASSERIFORMES | Malaconotidae | Black-crowned Tchagra | *Tchagra senegalus* | F*,eB*,G*,P* |  |
| 478 | PASSERIFORMES | Malaconotidae | Sulphur-breasted Bush-shrike | *Telophorus sulfureopectus* | F*,eB*,G*,X* |  |
| 479 | PASSERIFORMES | Malaconotidae | Rosy-patched Bushshrike | *Telophorus cruentus* | F*,eB*,G* |  |
| 480 | ACCIPITRIFORMES | Accipitridae | Bateleur | *Terathopius ecaudatus* | F*,eB*,G*,X*,M*,P* |  |
| 481 | PASSERIFORMES | Monarchidae | African Paradise-Flycatcher | *Terpsiphone viridis* | eB* |  |
| 482 | CHARADRIIFORMES | Sternidae | Greater Crested Tern | *Thalasseus bergi* | F*,eB*,G* |  |
| 483 | ANSERIFORMES | Anatidae | White-backed Duck | *Thalassornis leuconotus* | F*,eB*,G*,X* |  |
| 484 | PASSERIFORMES | Muscicapidae | Mocking Cliff-Chat | *Thamnolaea cinnamomeiventris* | F*,eB*,G*,M* |  |
| 485 | PASSERIFORMES | Turdidae | White-winged Cliff-Chat | *Thamnolaea semirufa* | F*,eB*,G*,X*,P* | E^†^ |
| 486 | PELECANIFORMES | Threskiornithidae | Sacred Ibis | *Threskiornis aethiopicus* | eB*,G*,X*,M* |  |
| 487 | BUCEROTIFORMES | Bucerotidae | Von der Decken's Hornbill | *Tockus deckeni* | F*,eB*,G*,X* |  |
| 488 | BUCEROTIFORMES | Bucerotidae | Eastern Yellow-billed Hornbill | *Tockus flavirostris* | G*,M*,X* |  |
| 489 | PICIFORMES | Lybiidae | D'Arnaud's Barbet | *Trachyphonus darnaudii* | F*,eB*,G*,X* |  |
| 490 | PICIFORMES | Lybiidae | Red-and-yellow Barbet | *Trachyphonus erythrocephalus* | F*,eB*,G*,X* |  |
| 491 | PICIFORMES | Lybiidae | Yellow-breasted Barbet | *Trachyphonus margaritatus* | F*,eB*,G*,X* |  |
| 492 | COLUMBIFORMES | Columbidae | African Green-Pigeon | *Treron calvus* | eB*,G*,M* |  |
| 493 | COLUMBIFORMES | Columbidae | Bruce's Green-Pigeon | *Treron waalia* | F*,eB*,G*,X*,M*,P* |  |
| 494 | PICIFORMES | Lybiidae | Red-fronted Barbet | *Tricholaema diademata* | F*,eB*,G*,X* |  |
| 495 | PICIFORMES | Lybiidae | Black-throated Barbet | *Tricholaema melanocephala* | F*,eB*,G*,X* |  |
| 496 | PASSERIFORMES | Leiothrichidae | Scaly Chatterer | *Turdoides aylmeri* | F*,eB*,G*,X* |  |
| 497 | PASSERIFORMES | Leiothrichidae | White-headed Babbler | *Turdoides leucocephala* | eB*,G* |  |
| 498 | PASSERIFORMES | Leiothrichidae | White-rumped Babbler | *Turdoides leucopygia* | F*,eB*,G*,X*,P* |  |
| 499 | PASSERIFORMES | Leiothrichidae | Brown Babbler | *Turdoides plebejus* | eB*,G*,X* |  |
| 500 | PASSERIFORMES | Leiothrichidae | Rufous Chatterer | *Turdoides rubiginosa* | F*,eB*,G*,X*,M* |  |
| 501 | PASSERIFORMES | Turdidae | Abyssinian Thrush | *Turdus abyssinicus* | F*,eB*,G*,X*,P* |  |
| 502 | PASSERIFORMES | Turdidae | Groundscraper Thrush | *Turdus ludoviciae* | eB*,G* | E^†^ |
| 503 | PASSERIFORMES | Turdidae | African Thrush | *Turdus pelios* | F*,eB*,G*,M*,P* |  |
| 504 | PASSERIFORMES | Turdidae | African Bare-eyed Thrush | *Turdus tephronotus* | F*,eB*,G*,X* |  |
| 505 | PASSERIFORMES | Turdidae | Groundscraper Thrush | *Turdus litsitsirupa* | F*,eB*,G*,X*,P* |  |
| 506 | COLUMBIFORMES | Columbidae | Black-billed Wood-Dove | *Turtur abyssinicus* | eB*,G*,X*,P* |  |
| 507 | COLUMBIFORMES | Columbidae | Blue-spotted Wood-Dove | *Turtur afer* | F*,eB*,G*,X*,P* |  |
| 508 | COLUMBIFORMES | Columbidae | Emerald-spotted Wood-Dove | *Turtur chalcospilos* | F*,eB*,G*,X*,M*,P* |  |
| 509 | COLUMBIFORMES | Columbidae | Tambourine Dove | *Turtur tympanistria* | F*,eB*,G*,X*,P* |  |
| 510 | STRIGIFORMES | Tytonidae | Western Barn Owl | *Tyto alba* | eB*,G*,M* |  |
| 511 | PASSERIFORMES | Estrildidae | Red-cheeked Cordonbleu | *Uraeginthus bengalus* | F*,eB*,G*,X*,P* |  |
| 512 | PASSERIFORMES | Estrildidae | Blue-capped Cordonbleu | *Uraeginthus cyanocephalus* | eB*,G* |  |
| 513 | PASSERIFORMES | Estrildidae | Purple Grenadier | *Uraeginthus ianthinogaster* | F*,eB*,G*,X* |  |
| 514 | COLIIFORMES | Coliidae | Blue-naped MouseB* | *Urocolius macrourus* | F*,eB*,G*,X*,P* |  |
| 515 | PASSERIFORMES | Cisticolidae | Red-fronted Warbler | *Urorhipis rufifrons* | F*,eB*,G*,X*,P* |  |
| 516 | CHARADRIIFORMES | Charadriidae | Crowned Lapwing | *Vanellus coronatus* | F*,eB*,G*,X*,M* |  |
| 517 | CHARADRIIFORMES | Charadriidae | Spot-breasted Lapwing | *Vanellus melanocephalus* | F*,eB*,G*,X*,M*,P* | E^†^ |
| 518 | CHARADRIIFORMES | Charadriidae | Spot-breasted Lapwing | *Vanellus melanopterus* | F*,eB*,G*,X*,M* |  |
| 519 | CHARADRIIFORMES | Charadriidae | African Wattled Lapwing | *Vanellus senegallus* | F*,eB*,G*,X*,M*,P* |  |
| 520 | CHARADRIIFORMES | Charadriidae | Spur-winged Lapwing | *Vanellus spinosus* | F*,eB*,G*,X*,M*,P* |  |
| 521 | CHARADRIIFORMES | Charadriidae | Black-headed Lapwing | *Vanellus tectus* | eB*,G*,X* |  |
| 522 | PASSERIFORMES | Viduidae | Village Indigobird | *Vidua chalybeata* | F*,eB*,G*,X*,P* |  |
| 523 | PASSERIFORMES | Viduidae | Straw-tailed Whydah | *Vidua fischeri* | eB*,G*,X* |  |
| 524 | PASSERIFORMES | Viduidae | Steel-blue Whydah | *Vidua hypocherina* | eB*,G* |  |
| 525 | PASSERIFORMES | Viduidae | Exclamatory Paradise-Whydah | *Vidua interjecta* | eB*,G*,X* |  |
| 526 | PASSERIFORMES | Viduidae | Baka Indigobird | *Vidua larvaticola* | eB*,X* |  |
| 527 | PASSERIFORMES | Viduidae | Pin-tailed Whydah | *Vidua macroura* | F*,eB*,G*,X*,M*,P* |  |
| 528 | PASSERIFORMES | Viduidae | Sahel Paradise-Whydah | *Vidua orientalis* | eB*,G* |  |
| 529 | PASSERIFORMES | Viduidae | Long-tailed Paradise-Whydah | *Vidua paradisaea* | F*,eB*,G*,X*,M* |  |
| 530 | PASSERIFORMES | Corvidae | Stresemann's Bush-Crow | *Zavattariornis stresemanni* | F*,eB*,G*,X*,M*,P* | E^†^ |
| 531 | PASSERIFORMES | Zosteropidae | Abyssinian White-eye | *Zosterops abyssinicus* | F*,eB*,G*,X*,M* |  |
| 532 | PASSERIFORMES | Zosteropidae | Montane White-eye | *Zosterops poliogastrus* | F*,eB*,G*,X*,M* |  |
| 533 | PASSERIFORMES | Zosteropidae | African Yellow White-eye | *Zosterops senegalensis* | F*,eB*,G*,X*,M* |  |

E^†^= Endemic birds to the region

P*- Puplished articles

Temmerman, L. De, Spanhove, T., Werbrouck, R., Werbrouck, D., Temmerman, L. De, Matson, D. & Davies, K. (2009). Ethiopia 08/10-02/11/2009.

Bot S., Gordijn R., Poelstra J.W., Radstaak S., van S.B. (2011). Birding trip report: Ethiopia, January 29 - February 21, 2011. 2011. Available at cloudbirders.com at <http://94.209.207.109/Apache/doc/doc00363.pdf>.

Bart De Keersmaecker (2008 & 2010). Available at Observations.org.

eB*- eBird (http://ebird.org/)

F*- Fieldwork

G*= GBIF (http://www.gbif.org/)

M*=Museum

X*= Xeno-Canto (http://www.xeno-canto.org/)
