# Supplementary material for: Factors determining species richness patterns of breeding birds along an elevational gradient in the Horn of Africa region
Source: Ecol Evol. 2019 Aug 5;9(17):9609–23. doi: 10.1002/ece3.5491 (PMC6745872; doi:10.1002/ece3.5491)
Supplement: Supplementary file 2 [file ECE3-9-9609-s002.docx]

**Factors Determining Species Richness Patterns of Breeding Birds along an Elevational Gradient in the Horn of Africa Region**

Ahunim Fenitie Abebe^1, 2, 3^, Tianlong Cai ^1, 3^, Melaku Wale^4^, Gang Song^1^, Jon Fjeldså^5*^ and Fumin Lei^1, 3, 6 *^

^1^Key Laboratory of the Zoological Systematics and Evolution, Institute of Zoology, Chinese Academy of Sciences, Beijing, 100101, China,

^2^Department of Biology, Faculty of Natural and Computational Sciences, Assosa University, Assosa, Ethiopia

^3^University of Chinese Academy of Sciences, Beijing, 100049, China

^4^College of Sciences, Bahir Dar University, Ethiopia

^5^Center for Macroecology, Evolution and Climate, Natural History Museum of Denmark, University of Copenhagen, DK-2100, Copenhagen, Denmark

^6^Center for Excellence in Animal Evolution and Genetics, Chinese Academy of Sciences, Kunming, 650223, China

*Correspondence: Fumin Lei, Institute of Zoology, Chinese Academy of Sciences, Beijing 100101, China.E-mail: leifm@ioz.ac.cn

**Table S2** Simple ordinary least squares (OLS) linear regression between bird species richness and explanatory variables along the elevational gradient in the Horn of Africa region. Significant parameters of each simple regression at *P* < 0.05 are marked in bold without taking auto-correlation in account. TAR, Mean Temperature Annual Range; AMT, Annual Mena Temperature; AP, Annual Precipitation; NDVI, Normalized Difference Vegetation Index; EVI, Enhanced Vegetation Index.

| Species Groups |  | AP | EVI | NDVI | TAR | Area | AMT | Elevation |
| --- | --- | --- | --- | --- | --- | --- | --- | --- |
| Dry side all birds | *r* | **-0.51235** | **0.5312** | **0.48918** | **-0.3692** | **0.46075** | **0.37361** | **-0.72136** |
|  | *P* | **0.001** | **0.0004** | **0.0006** | **0.0134** | **0.0018** | **0.0157** | **0.0001** |
| Dry side passerine birds | *r* | **-0.54964** | **0.53372** | **0.48829** | **-0.3106** | **0.39141** | **0.45846** | **-0.68226** |
|  | *P* | **0.0004** | **0.0001** | **0.0009** | **0.046** | **0.0122** | **0.0033** | **0.0001** |
| Dry side non-passerine birds | *r* | **-0.5683** | **0.49972** | **0.46045** | **-0.3651** | **0.52622** | **0.42489** | **-0.76194** |
|  | *P* | **0.0003** | **0.001** | **0.0013** | **0.0153** | **0.0006** | **0.0054** | **0.0001** |
| Eastern slope all birds | *r* | -0.039463 | **0.7954** | **0.8669** | **0.58389** | 0.038951 | 0.058117 | **-0.38524** |
|  | *P* | 0.7942 | **0.0001** | **0.0001** | **0.0001** | 0.7966 | 0.7123 | **0.0107** |
| Eastern slope passerine birds | *r* | -0.0012396 | **0.74107** | **0.83686** | **0.54369** | 0.010328 | 0.0057526 | **-0.39542** |
|  | *P* | 0.9939 | **0.0001** | **0.0001** | **0.0001** | 0.9436 | 0.9724 | **0.0071** |
| Eastern slope non-passerine birds | *r* | -0.017018 | **0.78601** | **0.87237** | **0.56909** | 0.019191 | 0.036714 | **-0.36628** |
|  | *P* | 0.9172 | **0.0001** | **0.0001** | **0.0001** | 0.9048 | 0.8145 | **0.0137** |
| Wet side all birds | *r* | **0.37301** | **0.80041** | **0.72077** | **-0.3519** | **0.60895** | 0.089002 | -0.2756 |
|  | *P* | **0.0149** | **0.0001** | **0.0001** | **0.0236** | **0.0001** | 0.5758 | 0.0764 |
| Wet side passerine birds | *r* | **0.57974** | **0.65327** | **0.53789** | **-0.3589** | **0.49953** | -0.13669 | -0.045321 |
|  | *P* | **0.0002** | **0.0001** | **0.0005** | **0.0198** | **0.0009** | 0.3968 | 0.7749 |
| Wet side non-passerine birds | *r* | **0.43442** | **0.74538** | **0.65469** | **-0.4296** | **0.53799** | -0.000279 | -0.094592 |
|  | *P* | **0.0047** | **0.0001** | **0.0001** | **0.005** | **0.0002** | 0.9984 | 0.5547 |
| Western slope all birds | *r* | -0.049335 | **0.60909** | **0.8803** | **-0.7919** | **0.78535** | **0.51514** | **-0.67645** |
|  | *P* | 0.7721 | **0.0001** | **0.0001** | **0.0001** | **0.0001** | **0.0007** | **0.0001** |
| Western slope passerine birds | *r* | 0.051883 | **0.57958** | **0.82362** | **0.72026** | **0.41549** | **-0.82257** | **-0.5872** |
|  | *P* | 0.743 | **0.0001** | **0.0001** | **0.0001** | **0.0076** | **0.0001** | **0.0003** |
| Western slope non-passerine birds | *r* | 0.090965 | **0.56502** | **0.81454** | **0.71404** | **0.3399** | **-0.70756** | **-0.49932** |
|  | *P* | 0.5719 | **0.0002** | **0.0001** | **0.0001** | **0.0298** | **0.0001** | **0.0014** |

**Table S3** Polynomial regressions for the bird species richness patterns along the elevational gradients in the Horn of Africa region for each species groups (richness as a function of elevation, elevation^2^ … Elevation^5^).

| Species Group |  | Dry side all birds | Dry side passerine birds | Dry side non-passerine birds | Eastern slope all birds | Eastern slope passerine birds | Eastern slope non-passerine birds | Wet side all birds | Wet side passerine birds | Wet side non-passerine birds | Western slope all birds | Western slope passerine birds | Western slope non-passerine birds |
| --- | --- | --- | --- | --- | --- | --- | --- | --- | --- | --- | --- | --- | --- |
| Order 1 | *AIC_c_* | 4.3381E05 | 1.4614E05 | 78407 | 4.6624E05 | 1.2707E05 | 47670 | 5.5861E05 | 1.264E05 | 1.1787E05 | 3.4013E05 | 1.011E05 | 1.1105E05 |
|  | *R^2^* | 0.52036 | 0.46548 | 0.58055 | 0.14841 | 0.10864 | 0.13416 | 0.075953 | 0.002054 | 0.035694 | 0.45758 | 0.3448 | 0.24932 |
|  | *P* | 3.2296E-07 | 2.3874E-06 | 2.7487E-08 | 0.0098124 | 0.030895 | 0.01447 | 0.077304 | 0.77843 | 0.2308 | .6566E-06 | 6.804E-05 | 0.00088917 |
| Order 2 | *AIC_c_* | 2.5732E05 | 78084 | 53023 | 1.2556E05 | 41782 | 12424 | 1.0189E05 | 24248 | 18900 | 1.0738E05 | 31289 | 31085 |
|  | *R^2^* | 0.7155 | 0.71442 | 0.71637 | 0.77068 | 0.70695 | 0.77443 | 0.83146 | 0.80861 | 0.84542 | 0.82877 | 0.79726 | 0.78991 |
|  | *P* | 2.7954E-10 | 2.9875E-10 | 2.65E-10 | 7.7481E-14 | 2.1818E-11 | 5.522E-14 | 8.3347E-16 | 2.2733E-14 | 1.5427E-16 | 6.6224E-15 | 1.5079E-13 | 1.3353E-13 |
| Order 3 | *AIC_c_* | 81412 | 26757 | 15433 | 89801 | 23386 | 9745.3 | 88091 | 20385 | 18011 | 60776 | 21495 | 21388 |
|  | *R^2^* | 0.91 | 0.90216 | 0.91748 | 0.836 | 0.83601 | 0.82314 | 0.85429 | 0.83912 | 0.85272 | .90309 | 0.86075 | 0.85548 |
|  | *P* | 7.5891E-18 | 3.1221E-17 | 1.7406E-18 | 9.3588E-16 | 2.2795E-15 | 4.2012E-15 | 5.9424E-16 | 9.5277E-15 | 7.2823E-16 | 2.6451E-18 | 1.7635E-15 | 1.3224E-15 |
| Order 4 | *AIC_c_* | 79582 | 25484 | 15380 | 72041 | 21288 | 7821.4 | 80662 | 20364 | 17857 | 41776 | 13878 | 14413 |
|  | *R^2^* | 0.91202 | .90683 | 0.91778 | 0.86844 | 0.85075 | 0.85814 | 0.86659 | 0.83931 | 0.854 | 0.9334 | 0.91013 | 0.90265 |
|  | *P* | 6.1318E-17 | 1.5711E-16 | 2.0187E-17 | 1.1931E-16 | 3.4591E-15 | 5.1317E-16 | 1.1154E-15 | 8.2168E-14 | 5.8327E-15 | 4.4678E-20 | 8.2526E-18 | 1.0645E-17 |
| Order  5 | *AIC_c_* | 25252 | 8750.4 | 4581.1 | 14006 | 21288 | 1473.5 | 53003 | 13732 | 12356 | 17863 | 7300.6 | 14103 |
|  | *R^2^* | 0.9721 | 0.96805 | 0.97557 | 0.97444 | 0.85075 | 0.97349 | 0.91235 | 0.89169 | 0.89903 | .97154 | 0.95278 | 0.90476 |
|  | *P* | 6.9957E-24 | 6.0765E-23 | 8.376E-25 | 3.6501E-29 | 3.4591E-15 | 7.2972E-29 | 5.2041E-18 | 6.635E-16 | 6.5003E-17 | 2.9741E-25 | 1.58E-21 | 7.1361E-17 |

All polynomial regressions are highly significant (*P* < 0.01). Bold numbers indicate the best reg­ression model selected by lowest AIC_c_ value and lowest P values to overcome over fitting.

**Table S4** Multiple OLS regression for Dry side all birds from all combinations. Parameters of each multiple regression that are significant at *P* < 0.05 are hued with bright green. It is sorted ascendingly by AIC values. TAR, Mean Temperature Annual Range; AMT, Annual Mena Temperature; AP, Annual Precipitation; NDVI, Normalized Difference Vegetation Index; EVI, Enhanced Vegetation Index.

| Model | Variables | No. vars. | AIC | *R*^2^ | R_adj2_ | *F* | DF | | *P* |
| --- | --- | --- | --- | --- | --- | --- | --- | --- | --- |
| 25 | TAR+ Area+AP+AMT+NDVI | 5 | 466.9674 | 0.8028 | 0.7738 | 27.68 | 5 | 34 | 4.452e-11 |
| 44 | TAR+ Area+AP+AMT+NDVI+ EVI | 6 | 468.742 | 0.8039 | 0.768 | 22.54 | 6 | 33 | 2.283e-10 |
| 41 | TAR+ Area+AP+AMT+ EVI | 5 | 469.5343 | 0.7897 | 0.7588 | 25.53 | 5 | 34 | 1.295e-10 |
| 43 | Area+AP+AMT+NDVI+ EVI | 5 | 479.7932 | 0.7282 | 0.6882 | 18.22 | 5 | 34 | 9.057e-09 |
| 39 | AMT+ Area+AP+ EVI | 4 | 479.9355 | 0.7133 | 0.6805 | 21.76 | 4 | 35 | 4.324e-09 |
| 24 | Area+AP+AMT+NDVI | 4 | 480.0013 | 0.7128 | 0.68 | 21.71 | 4 | 35 | 4.448e-09 |
| 40 | TAR+ Area+AP+NDVI+ EVI | 5 | 480.8021 | 0.7213 | 0.6803 | 17.6 | 5 | 34 | 1.372e-08 |
| 30 | TAR+Area+AP+EVI | 4 | 485.1722 | 0.6731 | 0.6358 | 18.02 | 4 | 35 | 4.052e-08 |
| 18 | AP+AMT+NDVI | 3 | 486.701 | 0.643 | 0.6133 | 21.61 | 3 | 36 | 3.526e-08 |
| 37 | AP+AMT+NDVI+ EVI | 4 | 487.4391 | 0.6541 | 0.6146 | 16.55 | 4 | 35 | 1.064e-07 |
| 19 | Area+AP+NDVI | 3 | 488.458 | 0.627 | 0.5959 | 20.17 | 3 | 36 | 7.684e-08 |
| 20 | AMT+ Area+AP | 3 | 488.458 | 0.627 | 0.5959 | 20.17 | 3 | 36 | 7.684e-08 |
| 21 | TAR+ Area+AP+NDVI | 4 | 488.458 | 0.627 | 0.5959 | 20.17 | 3 | 36 | 7.684e-08 |
| 22 | TAR+ Area+AP+AMT | 4 | 488.458 | 0.627 | 0.5959 | 20.17 | 3 | 36 | 7.684e-08 |
| 23 | TAR+AP+AMT+NDVI | 4 | 488.458 | 0.627 | 0.5959 | 20.17 | 3 | 36 | 7.684e-08 |
| 34 | TAR+ AP+AMT+EVI | 4 | 488.8007 | 0.6421 | 0.6012 | 15.7 | 4 | 35 | 1.898e-07 |
| 8 | AP+NDVI | 2 | 489.2166 | 0.6003 | 0.5787 | 27.79 | 2 | 37 | 4.279e-08 |
| 42 | TAR+AP+AMT+NDVI+ EVI | 5 | 489.4384 | 0.6541 | 0.6032 | 12.86 | 5 | 34 | 4.709e-07 |
| 38 | Area+AP+NDVI+ EVI | 4 | 489.6543 | 0.6344 | 0.5926 | 15.18 | 4 | 35 | 2.727e-07 |
| 14 | TAR+ AP+NDVI | 3 | 490.3797 | 0.6086 | 0.576 | 18.66 | 3 | 36 | 1.8e-07 |
| 55 | AP+NDVI+EVI | 3 | 490.5308 | 0.6071 | 0.5744 | 18.54 | 3 | 36 | 1.924e-07 |
| 33 | TAR+ AP+NDVI+EVI | 4 | 491.963 | 0.6127 | 0.5684 | 13.84 | 4 | 35 | 7.252e-07 |
| 11 | TAR+Area+AP | 3 | 492.3358 | 0.589 | 0.5547 | 17.2 | 3 | 36 | 4.276e-07 |
| 28 | AP+EVI | 2 | 492.4559 | 0.5666 | 0.5432 | 24.19 | 2 | 37 | 1.914e-07 |
| 48 | AP + Area +EVI | 3 | 493.0379 | 0.5817 | 0.5469 | 16.69 | 3 | 36 | 5.831e-07 |
| 51 | TAR+AP+EVI | 3 | 493.3814 | 0.5781 | 0.543 | 16.44 | 3 | 36 | 6.787e-07 |
| 10 | NDVI+AMT | 2 | 495.7595 | 0.5293 | 0.5039 | 20.8 | 2 | 37 | 8.822e-07 |
| 56 | TAR+Area+AMT+NDVI | 4 | 496.7564 | 0.5634 | 0.5135 | 11.29 | 4 | 35 | 5.47e-06 |
| 53 | NDVI + AMT+EVI | 3 | 497.1121 | 0.5369 | 0.4983 | 13.91 | 3 | 36 | 3.517e-06 |
| 54 | AP + AMT +EVI | 3 | 497.1121 | 0.5369 | 0.4983 | 13.91 | 3 | 36 | 3.517e-06 |
| 15 | TAR+ AP+AMT | 3 | 497.2044 | 0.5358 | 0.4971 | 13.85 | 3 | 36 | 3.662e-06 |
| 57 | NDVI+AMT+Area | 3 | 497.7574 | 0.5293 | 0.4901 | 13.5 | 3 | 36 | 4.671e-06 |
| 36 | TAR+AMT+NDVI+ EVI | 4 | 498.0668 | 0.5488 | 0.4973 | 10.64 | 4 | 35 | 9.477e-06 |
| 58 | TAR+Area+AMT+NDVI+EVI | 5 | 498.675 | 0.5642 | 0.5002 | 8.805 | 5 | 34 | 1.95e-05 |
| 9 | AP+AMT | 2 | 498.747 | 0.4928 | 0.4654 | 17.98 | 2 | 37 | 3.513e-06 |
| 45 | AMT+EVI | 2 | 499.0622 | 0.4888 | 0.4612 | 17.69 | 2 | 37 | 4.064e-06 |
| 17 | Area+AMT+NDVI+EVI | 4 | 499.1079 | 0.5369 | 0.484 | 10.15 | 4 | 35 | 1.465e-05 |
| 50 | TAR+AMT+EVI | 3 | 499.5446 | 0.5078 | 0.4668 | 12.38 | 3 | 36 | 1.025e-05 |
| 47 | AMT+Area+EVI | 3 | 500.9279 | 0.4905 | 0.4481 | 11.55 | 3 | 36 | 1.88e-05 |
| 32 | TAR+Area+AMT+EVI | 4 | 500.9373 | 0.5152 | 0.4598 | 9.3 | 4 | 35 | 3.143e-05 |
| 5 | TAR+AP | 2 | 502.3681 | 0.4448 | 0.4147 | 14.82 | 2 | 37 | 1.875e-05 |
| 6 | TAR+NDVI | 2 | 502.3681 | 0.4448 | 0.4147 | 14.82 | 2 | 37 | 1.875e-05 |
| 26 | Area+EVI | 2 | 504.7212 | 0.4111 | 0.3793 | 12.92 | 2 | 37 | 5.567e-05 |
| 3 | NDVI+Area | 2 | 505.464 | 0.4001 | 0.3676 | 12.34 | 2 | 37 | 7.849e-05 |
| 49 | NDVI+Area+EVI | 3 | 506.1773 | 0.4191 | 0.3707 | 8.656 | 3 | 36 | 0.000186 |
| 46 | TAR+Area +EVI | 3 | 506.2562 | 0.4179 | 0.3694 | 8.616 | 3 | 36 | 0.0001925 |
| 12 | TAR+Area+NDVI | 3 | 506.9762 | 0.4073 | 0.358 | 8.248 | 3 | 36 | 0.0002631 |
| 16 | TAR+ Area+ NDVI | 3 | 506.9762 | 0.4073 | 0.358 | 8.248 | 3 | 36 | 0.0002631 |
| 31 | TAR+Area+NDVI+EVI | 4 | 507.3715 | 0.4307 | 0.3656 | 6.618 | 4 | 35 | 0.0004471 |
| 35 | TAR+ Area+ NDVI+ EVI | 4 | 507.3715 | 0.4307 | 0.3656 | 6.618 | 4 | 35 | 0.0004471 |
| 7 | TAR+AMT | 2 | 507.9868 | 0.361 | 0.3265 | 10.45 | 2 | 37 | 0.0002521 |
| 13 | TAR+Area+AMT | 3 | 508.8729 | 0.3786 | 0.3268 | 7.31 | 3 | 36 | 0.0005982 |
| 27 | NDVI+EVI | 2 | 512.5029 | 0.2846 | 0.246 | 7.361 | 2 | 37 | 0.002035 |
| 29 | TAR+EVI | 2 | 512.5037 | 0.2846 | 0.246 | 7.361 | 2 | 37 | 0.002036 |
| 2 | AP+Area | 2 | 513.2206 | 0.2717 | 0.2323 | 6.901 | 2 | 37 | 0.002837 |
| 1 | TAR+Area | 2 | 514.2209 | 0.2533 | 0.2129 | 6.274 | 2 | 37 | 0.004505 |
| 52 | TAR+NDVI+EVI | 3 | 514.4092 | 0.2863 | 0.2268 | 4.814 | 3 | 36 | 0.006407 |
| 4 | AMT+Area | 2 | 515.9894 | 0.2195 | 0.1773 | 5.203 | 2 | 37 | 0.01021 |

**Table S5** Multiple OLS regression for Dry side passerine birds from all combinations. Parameters of each multiple regressions that are significant at *P* < 0.05 are hued bright with green. It is sorted ascendingly by AIC values. TAR, Mean Temperature Annual Range; AMT, Annual Mena Temperature; AP, Annual Precipitation; NDVI, Normalized Difference Vegetation Index; EVI, Enhanced Vegetation Index.

| Model | Variables | No. vars. | AIC | *R*^2^ | R_adj_^2^ | *F* | DF | | *P* |
| --- | --- | --- | --- | --- | --- | --- | --- | --- | --- |
| 25 | TAR+ Area+AP+AMT+NDVI | 5 | 412.5369 | 0.7809 | 0.7476 | 23.52 | 5 | 33 | 5.266e-10 |
| 44 | TAR+ Area+AP+AMT+NDVI+ EVI | 6 | 414.0362 | 0.7836 | 0.7431 | 19.32 | 6 | 32 | 2.237e-09 |
| 41 | TAR+ Area+AP+AMT+ EVI | 5 | 414.6161 | 0.7689 | 0.7338 | 21.95 | 5 | 33 | 1.242e-09 |
| 22 | TAR+ Area+AP+AMT | 4 | 421.3124 | 0.7111 | 0.6771 | 20.92 | 4 | 34 | 8.899e-09 |
| 21 | TAR+ Area+AP+NDVI | 4 | 422.5724 | 0.7016 | 0.6665 | 19.99 | 4 | 34 | 1.522e-08 |
| 40 | TAR+ Area+AP+NDVI+ EVI | 5 | 424.5689 | 0.7017 | 0.6564 | 15.52 | 5 | 33 | 7.377e-08 |
| 39 | AMT+ Area+AP+ EVI | 4 | 425.2665 | 0.6803 | 0.6427 | 18.09 | 4 | 34 | 4.788e-08 |
| 43 | Area+AP+AMT+NDVI+ EVI | 5 | 425.3602 | 0.6955 | 0.6494 | 15.08 | 5 | 33 | 1.019e-07 |
| 24 | Area+AP+AMT+NDVI | 4 | 426.0841 | 0.6735 | 0.6351 | 17.53 | 4 | 34 | 6.775e-08 |
| 30 | TAR+Area+AP+EVI | 4 | 427.8064 | 0.6588 | 0.6186 | 16.41 | 4 | 34 | 1.406e-07 |
| 19 | Area+AP+NDVI | 3 | 432.4912 | 0.595 | 0.5603 | 17.14 | 3 | 35 | 5.119e-07 |
| 54 | AP + AMT +EVI | 3 | 432.6246 | 0.5936 | 0.5587 | 17.04 | 3 | 35 | 5.429e-07 |
| 18 | AP+AMT+NDVI | 3 | 433.1722 | 0.5878 | 0.5525 | 16.64 | 3 | 35 | 6.91e-07 |
| 11 | TAR+Area+AP | 3 | 433.2021 | 0.5875 | 0.5522 | 16.62 | 3 | 35 | 7.001e-07 |
| 37 | AP+AMT+NDVI+ EVI | 4 | 433.2802 | 0.6074 | 0.5612 | 13.15 | 4 | 34 | 1.419e-06 |
| 38 | Area+AP+NDVI+ EVI | 4 | 433.4868 | 0.6053 | 0.5588 | 13.03 | 4 | 34 | 1.548e-06 |
| 34 | TAR+ AP+AMT+EVI | 4 | 434.6072 | 0.5938 | 0.546 | 12.42 | 4 | 34 | 2.479e-06 |
| 8 | AP+NDVI | 2 | 434.9771 | 0.5456 | 0.5204 | 21.61 | 2 | 36 | 6.822e-07 |
| 23 | TAR+AP+AMT+NDVI | 4 | 434.984 | 0.5898 | 0.5416 | 12.22 | 4 | 34 | 2.904e-06 |
| 42 | TAR+AP+AMT+NDVI+ EVI | 5 | 435.2676 | 0.6075 | 0.548 | 10.21 | 5 | 33 | 5.596e-06 |
| 48 | AP + Area +EVI | 3 | 435.7376 | 0.5598 | 0.5221 | 14.84 | 3 | 35 | 2.137e-06 |
| 55 | AP+NDVI+EVI | 3 | 436.0139 | 0.5567 | 0.5187 | 14.65 | 3 | 35 | 2.413e-06 |
| 14 | TAR+ AP+NDVI | 3 | 436.375 | 0.5526 | 0.5142 | 14.41 | 3 | 35 | 2.827e-06 |
| 28 | AP+EVI | 2 | 437.0917 | 0.5203 | 0.4936 | 19.52 | 2 | 36 | 1.81e-06 |
| 33 | TAR+ AP+NDVI+EVI | 4 | 437.6581 | 0.5607 | 0.509 | 10.85 | 4 | 34 | 8.898e-06 |
| 51 | TAR+AP+EVI | 3 | 438.5645 | 0.5267 | 0.4862 | 12.98 | 3 | 35 | 7.39e-06 |
| 10 | NDVI+AMT | 2 | 439.9043 | 0.4844 | 0.4558 | 16.91 | 2 | 36 | 6.631e-06 |
| 56 | TAR+Area+AMT+NDVI | 4 | 440.1769 | 0.5314 | 0.4763 | 9.639 | 4 | 34 | 2.541e-05 |
| 53 | NDVI + AMT+EVI | 3 | 441.0455 | 0.4956 | 0.4524 | 11.46 | 3 | 35 | 2.189e-05 |
| 57 | NDVI+AMT+Area | 3 | 441.5524 | 0.489 | 0.4452 | 11.17 | 3 | 35 | 2.731e-05 |
| 20 | AMT+ Area+AP | 3 | 442.0006 | 0.4831 | 0.4388 | 10.91 | 3 | 35 | 3.322e-05 |
| 58 | TAR+Area+AMT+NDVI+EVI | 5 | 442.034 | 0.5331 | 0.4624 | 7.536 | 5 | 33 | 8.211e-05 |
| 45 | AMT+EVI | 2 | 442.1192 | 0.4543 | 0.424 | 14.98 | 2 | 36 | 1.843e-05 |
| 36 | TAR+AMT+NDVI+ EVI | 4 | 442.4379 | 0.5034 | 0.445 | 8.617 | 4 | 34 | 6.487e-05 |
| 17 | Area+AMT+NDVI+EVI | 4 | 442.7203 | 0.4998 | 0.441 | 8.494 | 4 | 34 | 7.289e-05 |
| 50 | TAR+AMT+EVI | 3 | 443.3467 | 0.465 | 0.4191 | 10.14 | 3 | 35 | 5.974e-05 |
| 32 | TAR+Area+AMT+EVI | 4 | 443.8117 | 0.4856 | 0.4251 | 8.025 | 4 | 34 | 0.0001143 |
| 47 | AMT+Area+EVI | 3 | 444.0406 | 0.4554 | 0.4 | 9.755 | 3 | 35 | 8.08e-05 |
| 15 | TAR+ AP+AMT | 3 | 444.2333 | 0.4527 | 0.4058 | 9.649 | 3 | 35 | 8.787e-05 |
| 9 | AP+AMT | 2 | 444.7454 | 0.4163 | 0.3838 | 12.84 | 2 | 36 | 6.193e-05 |
| 5 | TAR+AP | 2 | 447.0758 | 0.3803 | 0.3459 | 11.05 | 2 | 36 | 0.0001816 |
| 26 | Area+EVI | 2 | 447.3092 | 0.3766 | 0.342 | 10.87 | 2 | 36 | 0.0002022 |
| 3 | NDVI+Area | 2 | 448.1035 | 0.3638 | 0.3284 | 10.29 | 2 | 36 | 0.0002918 |
| 49 | NDVI+Area+EVI | 3 | 448.3237 | 0.3922 | 0.3401 | 7.527 | 3 | 35 | 0.0005169 |
| 46 | TAR+Area +EVI | 3 | 449.2186 | 0.378 | 0.3247 | 7.091 | 3 | 3 | 0.00076 |
| 12 | TAR+Area+NDVI | 3 | 450.0449 | 0.3647 | 0.3103 | 6.698 | 3 | 35 | 0.001084 |
| 16 | TAR+ Area+ NDVI | 3 | 450.0449 | 0.3647 | 0.3103 | 6.698 | 3 | 35 | 0.001084 |
| 31 | TAR+Area+NDVI+EVI | 4 | 450.1195 | 0.3953 | 0.3242 | 5.557 | 4 | 34 | 0.001491 |
| 35 | TAR+ Area+ NDVI+ EVI | 4 | 450.1195 | 0.3953 | 0.3242 | 5.557 | 4 | 34 | 0.001491 |
| 13 | TAR+Area+AMT | 3 | 450.3484 | 0.3598 | 0.3049 | 6.556 | 3 | 35 | 0.001235 |
| 2 | AP+Area | 2 | 450.6191 | 0.3214 | 0.2837 | 8.525 | 2 | 36 | 0.0009317 |
| 7 | TAR+AMT | 2 | 451.3025 | 0.3094 | 0.271 | 8.064 | 2 | 36 | 0.001277 |
| 27 | NDVI+EVI | 2 | 452.2232 | 0.2929 | 0.2536 | 7.456 | 2 | 36 | 0.001953 |
| 29 | TAR+EVI | 2 | 452.3116 | 0.2913 | 0.2519 | 7.398 | 2 | 36 | 0.002035 |
| 52 | TAR+NDVI+EVI | 3 | 453.9316 | 0.2982 | 0.238 | 4.956 | 3 | 35 | 0.005688 |
| 6 | TAR+NDVI | 2 | 454.3676 | 0.2529 | 0.2114 | 6.094 | 2 | 36 | 0.005256 |
| 4 | AMT+Area | 2 | 456.4128 | 0.2127 | 0.169 | 4.863 | 2 | 36 | 0.01351 |
| 1 | TAR+Area | 2 | 458.0028 | 0.1799 | 0.1344 | 3.949 | 2 | 36 | 0.02814 |

**Table S6** Multiple OLS regression for Dry side none-passerine birds from all combinations. Parameters of each multiple regressions that are significant at *P* < 0.05 are hued with bright green .It is sorted ascendingly by AIC values. TAR, Mean Temperature Annual Range; AMT, Annual Mena Temperature; AP, Annual Precipitation; NDVI, Normalized Difference Vegetation Index; EVI, Enhanced Vegetation Index.

| Model | Variables | No. vars. | AIC | *R*^2^ | R_adj_^2^ | *F* | DF | | *P* |
| --- | --- | --- | --- | --- | --- | --- | --- | --- | --- |
| 25 | TAR+ Area+AP+AMT+NDVI | 5 | 400.4946 | 0.8173 | 0.7904 | 30.42 | 5 | 34 | 1.244e-11 |
| 44 | TAR+ Area+AP+AMT+NDVI+ EVI | 6 | 402.4479 | 0.8175 | 0.7843 | 24.64 | 6 | 33 | 7.177e-11 |
| 41 | TAR+ Area+AP+AMT+ EVI | 5 | 403.54 | 0.8028 | 0.7738 | 27.69 | 5 | 34 | 4.426e-11 |
| 22 | TAR+ Area+AP+AMT | 4 | 408.2077 | 0.7671 | 0.7405 | 28.82 | 4 | 35 | 1.217e-10 |
| 24 | Area+AP+AMT+NDVI | 4 | 412.3395 | 0.7417 | 0.7122 | 25.13 | 4 | 35 | 7.193e-10 |
| 43 | Area+AP+AMT+NDVI+ EVI | 5 | 412.8019 | 0.7515 | 0.7149 | 20.56 | 5 | 34 | 2.068e-09 |
| 39 | AMT+ Area+AP+ EVI | 4 | 413.2069 | 0.7361 | 0.7059 | 24.4 | 4 | 35 | 1.044e-09 |
| 21 | TAR+ Area+AP+NDVI | 4 | 413.9484 | 0.7311 | 0.7004 | 23.79 | 4 | 35 | 1.435e-09 |
| 40 | TAR+ Area+AP+NDVI+ EVI | 5 | 415.8415 | 0.7318 | 0.6924 | 18.56 | 5 | 34 | 7.254e-09 |
| 18 | AP+AMT+NDVI | 3 | 417.3963 | 0.6919 | 0.6662 | 26.95 | 3 | 36 | 2.574e-09 |
| 54 | AP + AMT +EVI | 3 | 418.172 | 0.6859 | 0.6597 | 26.2 | 3 | 36 | 3.634e-09 |
| 37 | AP+AMT+NDVI+ EVI | 4 | 418.4978 | 0.6987 | 0.6643 | 20.29 | 4 | 35 | 1.007e-08 |
| 38 | Area+AP+NDVI+ EVI | 4 | 418.4978 | 0.6987 | 0.6643 | 20.29 | 4 | 35 | 1.007e-08 |
| 23 | TAR+AP+AMT+NDVI | 4 | 419.1746 | 0.6936 | 0.6586 | 19.81 | 4 | 35 | 1.345e-08 |
| 34 | TAR+ AP+AMT+EVI | 4 | 420.0964 | 0.6865 | 0.6506 | 19.16 | 4 | 35 | 1.993e-08 |
| 42 | TAR+AP+AMT+NDVI+ EVI | 5 | 420.4463 | 0.6991 | 0.6549 | 15.8 | 5 | 34 | 4.819e-08 |
| 30 | TAR+Area+AP+EVI | 4 | 420.6585 | 0.682 | 0.6457 | 18.77 | 4 | 35 | 2.534e-08 |
| 8 | AP+NDVI | 2 | 421.8259 | 0.6382 | 0.6186 | 32.63 | 2 | 37 | 6.798e-09 |
| 19 | Area+AP+NDVI | 3 | 422.3186 | 0.6515 | 0.6225 | 22.44 | 3 | 36 | 2.293e-08 |
| 14 | TAR+ AP+NDVI | 3 | 422.5005 | 0.65 | 0.6208 | 22.28 | 3 | 36 | 2.486e-08 |
| 55 | AP+NDVI+EVI | 3 | 423.4617 | 0.6414 | 0.6116 | 21.47 | 3 | 36 | 3.809e-08 |
| 33 | TAR+ AP+NDVI+EVI | 4 | 424.3694 | 0.6511 | 0.6112 | 16.33 | 4 | 35 | 1.231e-07 |
| 28 | AP+EVI | 2 | 426.2743 | 0.5956 | 0.5737 | 27.25 | 2 | 37 | 5.32e-08 |
| 11 | TAR+Area+AP | 3 | 426.477 | 0.6134 | 0.5812 | 19.04 | 3 | 36 | 1.449e-07 |
| 51 | TAR+AP+EVI | 3 | 426.4893 | 0.6133 | 0.581 | 19.03 | 3 | 36 | 1.457e-07 |
| 52 | TAR+NDVI+EVI | 3 | 426.4893 | 0.6133 | 0.581 | 19.03 | 3 | 36 | 1.457e-07 |
| 15 | TAR+ AP+AMT | 3 | 427.3128 | 0.6052 | 0.5723 | 18.4 | 3 | 36 | 2.098e-07 |
| 48 | AP + Area +EVI | 3 | 427.7411 | 0.601 | 0.5677 | 18.07 | 3 | 36 | 2.536e-07 |
| 20 | AMT+ Area+AP | 3 | 429.2708 | 0.5854 | 0.5509 | 16.94 | 3 | 36 | 4.987e-07 |
| 9 | AP+AMT | 2 | 429.2737 | 0.5641 | 0.5406 | 23.94 | 2 | 37 | 2.13e-07 |
| 10 | NDVI+AMT | 2 | 430.3364 | 0.5524 | 0.5282 | 22.83 | 2 | 37 | 3.482e-07 |
| 56 | TAR+Area+AMT+NDVI | 4 | 431.772 | 0.5802 | 0.5322 | 12.09 | 4 | 35 | 2.825e-06 |
| 53 | NDVI + AMT+EVI | 3 | 431.9865 | 0.5563 | 0.5193 | 15.04 | 3 | 36 | 1.654e-06 |
| 57 | NDVI+AMT+Area | 3 | 432.1154 | 0.5548 | 0.5178 | 14.96 | 3 | 36 | 1.75e-06 |
| 36 | TAR+AMT+NDVI+ EVI | 4 | 432.2656 | 0.575 | 0.5264 | 11.84 | 4 | 35 | 3.477e-06 |
| 58 | TAR+Area+AMT+NDVI+EVI | 5 | 433.7602 | 0.5803 | 0.5186 | 9.402 | 5 | 34 | 1.07e-05 |
| 17 | Area+AMT+NDVI+EVI | 4 | 433.7762 | 0.5586 | 0.5082 | 11.07 | 4 | 35 | 6.56e-06 |
| 5 | TAR+AP | 2 | 434.2135 | 0.5068 | 0.4802 | 19.01 | 2 | 37 | 2.092e-06 |
| 50 | TAR+AMT+EVI | 3 | 434.3335 | 0.5295 | 0.4903 | 13.5 | 3 | 36 | 4.649e-06 |
| 45 | AMT+EVI | 2 | 434.6392 | 0.5015 | 0.4746 | 18.61 | 2 | 37 | 2.547e-06 |
| 47 | AMT+Area+EVI | 3 | 435.8539 | 0.5112 | 0.4705 | 12.55 | 3 | 36 | 9.07e-06 |
| 32 | TAR+Area+AMT+EVI | 4 | 436.2361 | 0.5306 | 0.477 | 9.891 | 4 | 35 | 1.837e-05 |
| 26 | Area+EVI | 2 | 439.5193 | 0.4369 | 0.4064 | 14.35 | 2 | 37 | 2.434e-05 |
| 3 | NDVI+Area | 2 | 439.7511 | 0.4336 | 0.403 | 14.16 | 2 | 37 | 2.709e-05 |
| 49 | NDVI+Area+EVI | 3 | 440.8763 | 0.4458 | 0.3997 | 9.655 | 3 | 36 | 8.175e-05 |
| 46 | TAR+Area +EVI | 3 | 440.95 | 0.4448 | 0.3986 | 9.615 | 3 | 36 | 8.442e-05 |
| 12 | TAR+Area+NDVI | 3 | 441.0718 | 0.4431 | 0.3967 | 9.549 | 3 | 36 | 8.902e-05 |
| 16 | TAR+ Area+ NDVI | 3 | 441.0718 | 0.4431 | 0.3967 | 9.549 | 3 | 36 | 8.902e-05 |
| 7 | TAR+AMT | 2 | 441.4038 | 0.4097 | 0.3778 | 12.84 | 2 | 37 | 5.818e-05 |
| 31 | TAR+Area+NDVI+EVI | 4 | 441.8936 | 0.4593 | 0.3975 | 7.433 | 4 | 35 | 0.0001918 |
| 35 | TAR+ Area+ NDVI+ EVI | 4 | 441.8936 | 0.4593 | 0.3975 | 7.433 | 4 | 35 | 0.0001918 |
| 13 | TAR+Area+AMT | 3 | 443.01 | 0.4155 | 0.3668 | 8.53 | 3 | 36 | 0.0002069 |
| 2 | AP+Area | 2 | 445.8652 | 0.3401 | 0.3044 | 9.532 | 2 | 37 | 0.0004581 |
| 1 | TAR+Area | 2 | 447.9008 | 0.3056 | 0.2681 | 8.142 | 2 | 37 | 0.001174 |
| 4 | AMT+Area | 2 | 449.0234 | 0.2858 | 0.2472 | 7.404 | 2 | 37 | 0.001974 |
| 29 | TAR+EVI | 2 | 450.7398 | 0.2545 | 0.2142 | 6.316 | 2 | 37 | 0.004366 |
| 27 | NDVI+EVI | 2 | 450.877 | 0.252 | 0.2115 | 6.231 | 2 | 37 | 0.004652 |
| 6 | TAR+NDVI | 2 | 452.4138 | 0.2227 | 0.1806 | 5.299 | 2 | 37 | 0.009469 |

**Table S7** Multiple OLS regression for eastern slope all birds from all combinations. Parameters of each multiple regressions that are significant at *P* < 0.05 are hued with bright green .It is sorted ascendingly by AIC values. TAR, Mean Temperature Annual Range; AMT, Annual Mena Temperature; AP, Annual Precipitation; NDVI, Normalized Difference Vegetation Index; EVI, Enhanced Vegetation Index.

| Model | Variables | No. vars. | AIC | *R*^2^ | R_adj_^2^ | *F* | DF | | *P* |
| --- | --- | --- | --- | --- | --- | --- | --- | --- | --- |
| 22 | TAR+ Area+AP+AMT | 4 | 462.2952 | 0.9078 | 0.8986 | 98.49 | 4 | 40 | < 2.2e-16 |
| 44 | TAR+ Area+AP+AMT+NDVI+ EVI | 6 | 463.2236 | 0.9139 | 0.9003 | 67.23 | 6 | 38 | < 2.2e-16 |
| 25 | TAR+ Area+AP+AMT+NDVI | 5 | 463.888 | 0.9087 | 0.8969 | 77.59 | 5 | 39 | < 2.2e-16 |
| 41 | TAR+ Area+AP+AMT+ EVI | 5 | 463.9569 | 0.9085 | 0.8968 | 77.46 | 5 | 39 | < 2.2e-16 |
| 15 | TAR+ AP+AMT | 3 | 482.5907 | 0.8487 | 0.8377 | 76.67 | 3 | 41 | < 2.2e-16 |
| 42 | TAR+AP+AMT+NDVI+ EVI | 5 | 483.1118 | 0.86 | 0.842 | 47.9 | 5 | 39 | 1.288e-15 |
| 23 | TAR+AP+AMT+NDVI | 4 | 483.7998 | 0.8514 | 0.8365 | 57.27 | 4 | 40 | 5e-16 |
| 58 | TAR+Area+AMT+NDVI+EVI | 5 | 484.0181 | 0.8571 | 0.8388 | 46.79 | 5 | 39 | 1.898e-15 |
| 34 | TAR+ AP+AMT+EVI | 4 | 484.4307 | 0.8493 | 0.8342 | 56.34 | 4 | 40 | 6.602e-16 |
| 36 | TAR+AMT+NDVI+ EVI | 4 | 485.9436 | 0.8441 | 0.8285 | 54.14 | 4 | 40 | 1.286e-15 |
| 21 | TAR+ Area+AP+NDVI | 4 | 492.8121 | 0.8184 | 0.8002 | 45.06 | 4 | 40 | 2.644e-14 |
| 40 | TAR+ Area+AP+NDVI+ EVI | 5 | 494.8049 | 0.8184 | 0.7951 | 35.16 | 5 | 39 | 1.905e-13 |
| 50 | TAR+AMT+EVI | 3 | 496.1818 | 0.7954 | 0.7804 | 53.12 | 3 | 41 | 3.495e-14 |
| 7 | TAR+AMT | 2 | 496.6027 | 0.7841 | 0.7738 | 76.25 | 2 | 42 | 1.049e-14 |
| 56 | TAR+Area+AMT+NDVI | 4 | 497.7089 | 0.7975 | 0.7773 | 39.39 | 4 | 40 | 2.275e-13 |
| 32 | TAR+Area+AMT+EVI | 4 | 497.7285 | 0.7974 | 0.7772 | 39.37 | 4 | 40 | 2.294e-13 |
| 13 | TAR+Area+AMT | 3 | 498.5172 | 0.7845 | 0.7687 | 49.75 | 3 | 41 | 1.006e-13 |
| 14 | TAR+ AP+NDVI | 3 | 498.6999 | 0.7836 | 0.7678 | 49.49 | 3 | 41 | 1.093e-13 |
| 49 | NDVI+Area+EVI | 3 | 498.989 | 0.7822 | 0.7663 | 49.08 | 3 | 41 | 1.246e-13 |
| 24 | Area+AP+AMT+NDVI | 4 | 499.2034 | 0.7907 | 0.7697 | 37.77 | 4 | 40 | 4.384e-13 |
| 38 | Area+AP+NDVI+ EVI | 4 | 499.9193 | 0.7873 | 0.7661 | 37.02 | 4 | 40 | 6.002e-13 |
| 43 | Area+AP+AMT+NDVI+ EVI | 5 | 500.6033 | .7935 | 0.767 | 29.96 | 5 | 39 | 2.25e-12 |
| 33 | TAR+ AP+NDVI+EVI | 4 | 500.6227 | 0.784 | 0.7624 | 36.29 | 4 | 40 | 8.172e-13 |
| 27 | NDVI+EVI | 2 | 500.6406 | 0.7638 | 0.7526 | 67.91 | 2 | 42 | 6.902e-14 |
| 17 | Area+AMT+NDVI+EVI | 4 | 500.6478 | 0.7839 | 0.7622 | 36.26 | 4 | 40 | 8.262e-13 |
| 52 | TAR+NDVI+EVI | 3 | 500.7198 | 0.7737 | 0.7571 | 46.72 | 3 | 41 | 2.727e-13 |
| 31 | TAR+Area+NDVI+EVI | 4 | 500.9541 | 0.7824 | 0.7606 | 35.95 | 4 | 40 | 9.451e-13 |
| 35 | TAR+ Area+ NDVI+ EVI | 4 | 500.9541 | 0.7824 | : 0.7606 | 35.95 | 4 | 40 | 9.451e-13 |
| 3 | NDVI+Area | 2 | 501.1021 | 0.7614 | 0.75 | 67 | 2 | 42 | 8.561e-14 |
| 53 | NDVI + AMT+EVI | 3 | 501.3169 | 0.7706 | 0.75 | 45.92 | 3 | 41 | 3.573e-13 |
| 55 | AP+NDVI+EVI | 3 | 501.5074 | 0.7697 | 0.7528 | 45.67 | 3 | 41 | 3.894e-13 |
| 10 | NDVI+AMT | 2 | 501.8275 | 0.7575 | 0.7459 | 65.59 | 2 | 42 | 1.201e-13 |
| 19 | Area+AP+NDVI | 3 | 502.0258 | 0.767 | 0.75 | 44.99 | 3 | 41 | 4.923e-13 |
| 6 | TAR+NDVI | A | 502.1327 | 0.7558, | 0.7442 | 65.01 | 2 | 42 | 1.385e-13 |
| 18 | AP+AMT+NDVI | 3 | 502.1623 | 0.7663 | 0.7492 | 44.81 | 3 | 41 | 5.237e-13 |
| 8 | AP+NDVI | 2 | 502.534 | 0.7536 | 0.7419 | 64.24 | 2 | 42 | 1.67e-13 |
| 12 | TAR+Area+NDVI | 3 | 503.0175 | 0.7618 | 0.7444 | 43.71 | 3 | 41 | 7.711e-13 |
| 16 | TAR+ Area+ NDVI | 3 | 503.0175 | 0.7618 | 0.7444 | 43.71 | 3 | 41 | 7.711e-13 |
| 57 | NDVI+AMT+Area | 3 | 503.102 | 0.7614 | 0.7439 | 43.6 | 3 | 41 | 8.011e-13 |
| 37 | AP+AMT+NDVI+ EVI | 4 | 503.2459 | 0.771 | 0.7481 | 33.67 | 4 | 40 | 2.581e-12 |
| 30 | TAR+Area+AP+EVI | 4 | 503.4529 | 0.77 | 0.7469 | 33.47 | 4 | 40 | 2.826e-12 |
| 51 | TAR+AP+EVI | 3 | 508.7301 | 0.7296 | 0.7098 | 36.87 | 3 | 41 | 1.02e-11 |
| 39 | AMT+ Area+AP+ EVI | 4 | 515.9994 | 0.696 | 0.6656 | 22.89 | 4 | 40 | 6.791e-10 |
| 54 | AP + AMT +EVI | 3 | 517.0402 | 0.6747 | 0.6509 | 28.35 | 3 | 41 | 4.332e-10 |
| 29 | TAR+EVI | 2 | 520.1555 | 0.6356 | 0.6182 | 36.62 | 2 | 42 | 6.224e-10 |
| 45 | AMT+EVI | 2 | 520.4251 | 0.6334 | 0.6159 | 36.28 | 2 | 42 | 7.059e-10 |
| 28 | AP+EVI | 2 | 520.4448 | 0.6332 | 0.6157 | 36.25 | 2 | 42 | 7.124e-10 |
| 26 | Area+EVI | 2 | 520.4798 | 0.6329 | 0.6154 | 36.21 | 2 | 42 | 7.241e-10 |
| 48 | AP + Area +EVI | 3 | 521.8259 | 0.6382 | 0.6118 | 24.11 | 3 | 41 | 3.735e-09 |
| 46 | TAR+Area +EVI | 3 | 521.9383 | 0.6373 | 0.6108 | 24.02 | 3 | 41 | 3.929e-09 |
| 47 | AMT+Area+EVI | 3 | 522.4174 | 0.6334 | 0.6066 | 23.62 | 3 | 41 | 4.873e-09 |
| 9 | AP+AMT | 2 | 552.4221 | 0.2535 | 0.218 | 7.131 | 2 | 42 | 0.002156 |
| 20 | AMT+ Area+AP | 3 | 554.1155 | 0.2586 | 0.2043 | 4.766 | 3 | 41 | 0.006101 |
| 4 | AMT+Area | 2 | 561.0868 | 0.095 | 0.0519 | 2.204 | 2 | 42 | 0.1229 |
| 5 | TAR+AP | 2 | 565.2613 | 0.007026 | -0.04026 | 0.1486 | 2 | 42 | 0.8624 |
| 1 | TAR+Area | 2 | 565.4212 | 0.003492 | -0.0439 | 0.07359 | 2 | 42 | 0.9292 |
| 2 | AP+Area | 2 | 565.504 | 0.001656 | -0.04588 | 0.03484 | 2 | 42 | 0.9658 |
| 11 | TAR+Area+AP | 3 | 567.2131 | 0.008088 | -0.06449 | 0.1114 | 3 | 41 | 0.9529 |

**Table S8** Multiple OLS regression for eastern slope Passerine birds from all combinations. Parameters of each multiple regressions that are significant at *P* < 0.05 are hued with green .It is sorted ascendingly by AIC values. TAR, Mean Temperature Annual Range; AMT, Annual Mena Temperature; AP, Annual Precipitation; NDVI, Normalized Difference Vegetation Index; EVI, Enhanced Vegetation Index.

| Model | Variables | No. vars. | AIC | *R*^2^ | R_adj_^2^ | *F* | DF | | *P* |
| --- | --- | --- | --- | --- | --- | --- | --- | --- | --- |
| 25 | TAR+ Area+AP+AMT+NDVI | 5 | 430.4004 | 0.8042 | 0.7784 | 31.21 | 5 | 38 | 1.771e-12 |
| 24 | Area+AP+AMT+NDVI | 4 | 431.1381 | 0.7916 | 0.7702 | 37.03 | 4 | 39 | 8.613e-13 |
| 44 | TAR+ Area+AP+AMT+NDVI+ EVI | 6 | 432.2851 | 0.8047 | 0.773 | 25.4 | 6 | 37 | 1.005e-11 |
| 43 | Area+AP+AMT+NDVI+ EVI | 5 | 433.1373 | 0.7916 | 0.7642 | 28.87 | 5 | 38 | 5.649e-12 |
| 21 | TAR+ Area+AP+NDVI | 4 | 433.9077 | 0.778 | 0.7553 | 34.18 | 4 | 39 | 2.892e-12 |
| 40 | TAR+ Area+AP+NDVI+ EVI | 5 | 435.0697 | 0.7822 | 0.7536 | 27.3 | 5 | 38 | 1.28e-11 |
| 38 | Area+AP+NDVI+ EVI | 4 | 438.8928 | 0.7514 | 0.7259 | 29.47 | 4 | 39 | 2.55e-11 |
| 18 | AP+AMT+NDVI | 3 | 439.013 | 0.7391 | 0.7196 | 37.78 | 3 | 40 | 9.488e-12 |
| 49 | NDVI+Area+EVI | 3 | 439.1846 | 0.7381 | 0.7 | 37.58 | 3 | 40 | 1.025e-11 |
| 23 | TAR+AP+AMT+NDVI | 4 | 439.7414 | 0.7466 | 0.7206 | 28.72 | 4 | 39 | 3.692e-11 |
| 27 | NDVI+EVI | 2 | 440.0631 | 0.7204 | 0.7068 | 52.82 | 2 | 41 | 4.504e-12 |
| 17 | Area+AMT+NDVI+EVI | 4 | 440.826 | 0.7402 | 0.7136 | 27.79 | 4 | 39 | 5.923e-11 |
| 37 | AP+AMT+NDVI+ EVI | 4 | 440.857 | 0.7401 | 0.7134 | 27.76 | 4 | 39 | 6.003e-11 |
| 53 | NDVI + AMT+EVI | 3 | 441.1029 | 0.7264 | 0.7059 | 35.41 | 3 | 40 | 2.433e-11 |
| 31 | TAR+Area+NDVI+EVI | 4 | 441.1458 | 0.7383 | 0.7115 | 27.51 | 4 | 39 | 6.808e-11 |
| 35 | TAR+ Area+ NDVI+ EVI | 4 | 441.1458 | 0.7383 | 0.7115 | 27.51 | 4 | 39 | 6.808e-11 |
| 42 | TAR+AP+AMT+NDVI+ EVI | 5 | 441.5214 | 0.7478 | 0.7147 | 22.54 | 5 | 38 | 1.948e-10 |
| 55 | AP+NDVI+EVI | 3 | 441.6321 | 0.7231 | 0.7024 | 34.83 | 3 | 40 | 3.089e-11 |
| 41 | TAR+ Area+AP+AMT+ EVI | 5 | 441.8082 | 0.7462 | 0.7128 | 22.34 | 5 | 38 | 2.198e-10 |
| 19 | Area+AP+NDVI | 3 | 441.8303 | 0.7219 | 0.701 | 34.61 | 3 | 40 | 3.377e-11 |
| 3 | NDVI+Area | 2 | 441.9501 | 0.7082 | 0.6939 | 49.74 | 2 | 41 | 1.085e-11 |
| 52 | TAR+NDVI+EVI | 3 | 441.977 | 0.721 | 0.7 | 34.45 | 3 | 40 | 3.608e-11 |
| 58 | TAR+Area+AMT+NDVI+EVI | 5 | 442.3349 | 0.7431 | 0.7093 | 21.99 | 5 | 38 | 2.744e-10 |
| 10 | NDVI+AMT | 2 | 442.4312 | 0.705 | 0.6906 | 48.98 | 2 | 41 | 1.358e-11 |
| 36 | TAR+AMT+NDVI+ EVI | 4 | 442.6894 | 0.729 | 0.7012 | 26.23 | 4 | 39 | 1.333e-10 |
| 6 | TAR+NDVI | 2 | 442.7757 | 0.7026 | 0.6881 | 48.44 | 2 | 41 | 1.594e-11 |
| 8 | AP+NDVI | 2 | 443.0767 | 0.7006 | 0.686 | 47.97 | 2 | 41 | 1.834e-11 |
| 39 | AMT+ Area+AP+ EVI | 4 | 443.3004 | 0.7252 | 0.697 | 25.73 | 4 | 39 | 1.74e-10 |
| 33 | TAR+ AP+NDVI+EVI | 4 | 443.5868 | 0.7234 | 0.6951 | 25.5 | 4 | 39 | 1.97e-10 |
| 12 | TAR+Area+NDVI | 3 | 443.8219 | 0.709 | 0.6872 | 32.49 | 3 | 4 | 8.28e-11 |
| 16 | TAR+ Area+ NDVI | 3 | 443.8219 | 0.709 | 0.6872 | 32.49 | 3 | 40 | 8.28e-11 |
| 57 | NDVI+AMT+Area | 3 | 443.9497 | 0.7082 | 0.6863 | 32.35 | 3 | 40 | 8.77e-11 |
| 14 | TAR+ AP+NDVI | 3 | 444.7347 | 0.7029 | 0.6806 | 31.55 | 3 | 40 | 1.249e-10 |
| 56 | TAR+Area+AMT+NDVI | 4 | 445.274 | 0.7126 | 0.6831 | 24.18 | 4 | 39 | 4.104e-10 |
| 54 | AP + AMT +EVI | 3 | 449.4839 | 0.669 | 0.6442 | 26.95 | 3 | 40 | 1.057e-09 |
| 34 | TAR+ AP+AMT+EVI | 4 | 450.7642 | : 0.6744 | 0.641 | 20.2 | 4 | 39 | 4.443e-09 |
| 30 | TAR+Area+AP+EVI | 4 | 453.0467 | 0.6571 | 0.6219 | 18.68 | 4 | 39 | 1.192e-08 |
| 29 | TAR+EVI | 2 | 460.5578 | 0.5545 | 0.5328 | 25.52 | 2 | 41 | 6.317e-08 |
| 28 | AP+EVI | 2 | 460.8637 | 0.5514 | 0.5296 | 25.2 | 2 | 41 | 7.285e-08 |
| 45 | AMT+EVI | 2 | 460.9359 | 0.5507 | 0.5288 | 25.13 | 2 | 41 | 7.534e-08 |
| 26 | Area+EVI | 2 | 461.0831 | 0.5492 | 0.5272 | 24.97 | 2 | 41 | 8.069e-08 |
| 51 | TAR+AP+EVI | 3 | 461.0954 | 0.5691 | 0.5368 | : 17.61 | 3 | 40 | 1.921e-07 |
| 48 | AP + Area +EVI | 3 | 462.1407 | 0.5587 | 0.5257 | 16.88 | 3 | 40 | 3.064e-07 |
| 50 | TAR+AMT+EVI | 3 | 462.519 | 0.5549 | 0.52 | 16.62 | 3 | 40 | 3.628e-07 |
| 46 | TAR+Area +EVI | 3 | 462.5373 | 0.5548 | 0.5214 | 16.61 | 3 | 40 | 3.657e-07 |
| 47 | AMT+Area+EVI | 3 | 462.7468 | 0.5526 | 0.5191 | 16.47 | 3 | 40 | 4.016e-07 |
| 32 | TAR+Area+AMT+EVI | 4 | 464.5116 | 0.555 | 0.5094 | 12.16 | 4 | 39 | 1.643e-06 |
| 7 | TAR+AMT | 2 | 495.7867 | 0.007965 | -0.04043 | 0.1646 | 2 | 41 | 0.8488 |
| 5 | TAR+AP | 2 | 495.8389 | 0.006787 | -0.04166 | 0.1401 | 2 | 41 | 0.8697 |
| 1 | TAR+Area | 2 | 495.944 | 0.004413 | -0.04415 | 0.09086 | 2 | 41 | 0.9133 |
| 2 | AP+Area | 2 | 496.1244 | 0.0003218 | -0.04844 | 0.006599 | 2 | 41 | 0.9934 |
| 9 | AP+AMT | 2 | 496.1264 | 0.0002769 | -0.04849 | 0.005677 | 2 | 41 | 0.9943 |
| 4 | AMT+Area | 2 | 496.1333 | 0.0001206 | -0.04865 | 0.002472 | 2 | 41 | 0.9975 |
| 13 | TAR+Area+AMT | 3 | 497.1827 | 0.02149 | -0.0519 | 0.2928 | 3 | 40 | 0.8303 |
| 11 | TAR+Area+AP | 3 | 497.352 | 0.01772 | -0.05595 | 0.2405 | 3 | 40 | 0.8676 |
| 15 | TAR+ AP+AMT | 3 | 497.7589 | 0.008593 | -0.06576 | 0.1156 | 3 | 40 | 0.9505 |
| 20 | AMT+ Area+AP | 3 | 498.1019 | 0.0008338 | -0.0741 | 0.01113 | 3 | 40 | 0.9984 |
| 22 | TAR+ Area+AP+AMT | 4 | 499.1306 | 0.02265 | -0.07759 | 0.2259 | 4 | 39 | 0.9222 |

**Table S9** Multiple OLS regression for eastern slope non-passerine birds from all combinations. Parameters of each multiple regressions that are significant at *P* < 0.05 are hued with green .It is sorted ascendingly by AIC values. TAR, Mean Temperature Annual Range; AMT, Annual Mena Temperature; AP, Annual Precipitation; NDVI, Normalized Difference Vegetation Index; EVI, Enhanced Vegetation Index.

| Model | Variables | No. vars. | AIC | *R*^2^ | R_adj_^2^ | *F* | DF | | *P* |
| --- | --- | --- | --- | --- | --- | --- | --- | --- | --- |
| 25 | TAR+ Area+AP+AMT+NDVI | 5 | 385.3337 | 0.8424 | 0.8222 | 41.69 | 5 | 39 | 1.255e-14 |
| 24 | Area+AP+AMT+NDVI | 4 | 386.1597 | 0.8322 | 0.8154 | 49.59 | 4 | 40 | 5.542e-15 |
| 44 | TAR+ Area+AP+AMT+NDVI+ EVI | 6 | 387.2514 | 0.8427 | 0.8178 | 33.92 | 6 | 38 | 8.328e-14 |
| 43 | Area+AP+AMT+NDVI+ EVI | 5 | 388.1521 | 0.8322 | 0.8107 | 38.69 | 5 | 39 | 4.186e-14 |
| 21 | TAR+ Area+AP+NDVI | 4 | 388.4357 | 0.8235 | 0.8058 | 46.65 | 4 | 40 | 1.509e-14 |
| 40 | TAR+ Area+AP+NDVI+ EVI | 5 | 389.5966 | 0.8267 | 0.8045 | 37.22 | 5 | 39 | 7.754e-14 |
| 49 | NDVI+Area+EVI | 3 | 392.2459 | 0.7991 | 0.7844 | 54.37 | 3 | 41 | 2.395e-14 |
| 18 | AP+AMT+NDVI | 3 | 392.8733 | 0.7963 | 0.7814 | 53.43 | 3 | 3.182e-14 |  |
| 38 | Area+AP+NDVI+ EVI | 4 | 393.3988 | 0.8029 | 0.7832 | 40.73 | 4 | 40 | 1.337e-13 |
| 23 | TAR+AP+AMT+NDVI | 4 | 393.9132 | 0.8006 | 0.7807 | 40.16 | 4 | 40 | 1.676e-13 |
| 31 | TAR+Area+NDVI+EVI | 4 | 394.1457 | 0.7996 | 0.7795 | 39.9 | 4 | 40 | 1.857e-13 |
| 35 | TAR+ Area+ NDVI+ EVI | 4 | 394.1457 | 0.7996 | 0.7795 | 39.9 | 4 | 40 | 1.857e-13 |
| 17 | Area+AMT+NDVI+EVI | 4 | 394.2407 | 0.7992 | 0.7791 | 39.79 | 4 | 40 | 1.936e-13 |
| 53 | NDVI + AMT+EVI | 3 | 394.458 | 0.789 | 0.7736 | 51.11 | 3 | 41 | 6.521e-14 |
| 37 | AP+AMT+NDVI+ EVI | 4 | 394.6706 | 0.7972 | 0.777 | 39.32 | 4 | 40 | 2.338e-13 |
| 3 | NDVI+Area | 2 | 395.2578 | 0.7755 | 0.7648 | 72.53 | 2 | 42 | 2.38e-14 |
| 55 | AP+NDVI+EVI | 3 | 395.4478 | 0.7843 | 0.7685 | 49.7 | 3 | 41 | 1.021e-13 |
| 27 | NDVI+EVI | 2 | 395.5416 | 0.7741 | 0.7633 | 71.94 | 2 | 42 | 2.717e-14 |
| 10 | NDVI+AMT | 2 | 395.5808 | 0.7739 | 0.7631 | 71.86 | 2 | 42 | 2.767e-14 |
| 58 | TAR+Area+AMT+NDVI+EVI | 5 | 395.5828 | 0.8021 | 0.7767 | 31.61 | 5 | 39 | 9.944e-13 |
| 42 | TAR+AP+AMT+NDVI+ EVI | 5 | 395.653 | 0.8018 | 0.7764 | 31.55 | 5 | 39 | .025e-12 |
| 36 | TAR+AMT+NDVI+ EVI | 4 | 396.1455 | 0.7905 | 0.7695 | 37.73 | 4 | 40 | 4.468e-13 |
| 19 | Area+AP+NDVI | 3 | 396.4026 | 0.7797 | 0.7636 | 48.37 | 3 | 41 | 1.573e-13 |
| 12 | TAR+Area+NDVI | 3 | 396.5827 | 0.7788 | 0.7626 | 48.12 | 3 | 41 | 1.706e-13 |
| 16 | TAR+ Area+ NDVI | 3 | 396.5827 | 0.7788 | 0.7626 | 48.12 | 3 | 41 | 1.706e-13 |
| 52 | TAR+NDVI+EVI | 3 | 396.6849 | 0.7783 | 0.7621 | 47.98 | 3 | 41 | 1.787e-13 |
| 6 | TAR+NDVI | A | 396.7404 | 0.768 | 0.7569 | 69.5 | 2 | 42 | 4.753e-14 |
| 41 | TAR+ Area+AP+AMT+ EVI | 5 | 397.028 | 0.7956 | 0.7694 | 30.36 | 5 | 39 | 1.839e-12 |
| 57 | NDVI+AMT+Area | 3 | 397.0602 | 0.7765 | 0.7601 | 47.47 | 3 | 41 | 2.118e-13 |
| 8 | AP+NDVI | 2 | 397.1474 | 0.7658 | 0.7547 | 68.69 | 2 | 42 | 5.748e-14 |
| 33 | TAR+ AP+NDVI+EVI | 4 | 397.44 | 0.7844 | 0.7628 | : 36.37 | 4 | 40 | 7.884e-13 |
| 56 | TAR+Area+AMT+NDVI | 4 | 398.1889 | 0.7807 | .7588 | 35.61 | 4 | 40 | 1.095e-12 |
| 14 | TAR+ AP+NDVI | 3 | 398.6371 | 0.7685 | 0.7515 | 45.37 | 3 | 41 | 4.323e-13 |
| 39 | AMT+ Area+AP+ EVI | 4 | 398.7306 | 0.7781 | 0.7559 | 35.06 | 4 | 40 | 1.389e-12 |
| 54 | AP + AMT +EVI | 3 | 404.4085 | 0.7368 | 0.7175 | 38.26 | 3 | 41 | 5.875e-12 |
| 34 | TAR+ AP+AMT+EVI | 4 | 405.8503 | 0.7401 | 0.7141 | 28.47 | 4 | 40 | 3.136e-11 |
| 30 | TAR+Area+AP+EVI | 4 | 408.0774 | 0.7269 | 0.6995 | 26.61 | 4 | 40 | 8.298e-11 |
| 29 | TAR+EVI | 2 | 415.3434 | 0.6492 | 0.6325 | 38.86 | 2 | 42 | 2.801e-10 |
| 45 | AMT+EVI | 2 | 415.8394 | 0.6453 | 0.6284 | 38.2 | 2 | 42 | 3.53e-10 |
| 26 | Area+EVI | 2 | 416.4023 | 0.6408 | 0.6237 | 37.46 | 2 | 42 | 4.591e-10 |
| 51 | TAR+AP+EVI | 3 | 416.47 | 0.6559 | 0.6307 | 26.05 | 3 | 41 | 1.354e-09 |
| 28 | AP+EVI | 2 | 416.5674 | 0.6395 | 0.6223 | 37.25 | 2 | 42 | 4.958e-10 |
| 46 | TAR+Area +EVI | 3 | 417.3215 | 0.6493 | 0.6237 | 25.31 | 3 | 41 | 1.987e-09 |
| 50 | TAR+AMT+EVI | 3 | 417.3367 | 0.6492 | 0.6235 | 25.29 | 3 | 41 | 2e-09 |
| 47 | AMT+Area+EVI | 3 | 417.6599 | 0.6467 | 0.6208 | 25.01 | 3 | 41 | 2.313e-09 |
| 48 | AP + Area +EVI | 3 | 417.6599 | 0.6467 | 0.6208 | 25.01 | 3 | 41 | 2.313e-09 |
| 32 | TAR+Area+AMT+EVI | 4 | 419.3051 | 0.6495 | 0.6144 | 18.53 | 4 | 40 | 1.098e-08 |
| 5 | TAR+AP | 2 | 461.7 | 0.01713 | -0.02967 | 0.366 | 2 | 42 | 0.6957 |
| 7 | TAR+AMT | 2 | 461.7072 | 0.01697 | -0.02984 | 0.3626 | 2 | 42 | 0.698 |
| 1 | TAR+Area | 2 | 461.9008 | 0.01274 | -0.03428 | 0.2709 | 2 | 42 | 0.764 |
| 9 | AP+AMT | 2 | 462.213 | 0.005864 | -0.04148 | 0.1239 | 2 | 42 | 0.8838 |
| 4 | AMT+Area | 2 | 462.4061 | 0.001588 | -0.04596 | 0.0334 | 2 | 42 | 0.9672 |
| 2 | AP+Area | 2 | 462.461 | 0.0003695 | -0.04723 | 0.007762 | 2 | 42 | 0.9923 |
| 11 | TAR+Area+AP | 3 | 462.829 | 0.03597 | -0.03457 | 0.51 | 3 | 41 | 0.6776 |
| 13 | TAR+Area+AMT | 3 | 463.0452 | 0.03133 | -0.03955 | 0.442 | 3 | 41 | 0.7242 |
| 15 | TAR+ AP+AMT | 3 | 463.6978 | 0.01718 | -0.05473 | 0.2389 | 3 | 41 | 0.8687 |
| 20 | AMT+ Area+AP | 3 | 464.1673 | 0.006872 | -0.0658 | 0.09456 | 3 | 41 | 0.9626 |
| 22 | TAR+ Area+AP+AMT | 4 | 464.7455 | 0.03776 | -0.05846 | 0.3924 | 4 | 40 | 0.8128 |

**Table S10** Multiple OLS regression for Wet side all birds from all combinations. Parameters of each multiple regressions that are significant at *P* < 0.05 are hued with bright green .It is sorted ascendingly by AIC values. TAR, Mean Temperature Annual Range; AMT, Annual Mena Temperature; AP, Annual Precipitation; NDVI, Normalized Difference Vegetation Index; EVI, Enhanced Vegetation Index.

| Model | Variables | No. vars. | AIC | *R*^2^ | R_adj_^2^ | *F* | DF | | *P* |
| --- | --- | --- | --- | --- | --- | --- | --- | --- | --- |
| 56 | TAR+Area+AMT+NDVI | 4 | 430.6275 | 0.9133 | 0.9039 | 97.42 | 4 | 37 | < 2.2e-16 |
| 57 | NDVI+AMT+Area | 3 | 431.4616 | 0.9072 | 0.8999 | 123.9 | 3 | 38 | < 2.2e-16 |
| 58 | TAR+Area+AMT+NDVI+EVI | 5 | 432.4592 | 0.9136 | 0.9016 | 76.16 | 5 | 36 | < 2.2e-16 |
| 25 | TAR+ Area+AP+AMT+NDVI | 5 | 432.5823 | 0.9134 | 0.9013 | 75.92 | 5 | 36 | < 2.2e-16 |
| 23 | TAR+AP+AMT+NDVI | 4 | 432.9354 | 0.9084 | 0.8985 | 91.71 | 4 | 37 | < 2.2e-16 |
| 17 | Area+AMT+NDVI+EVI | 4 | 433.2171 | 0.9078 | 0.8978 | 91.04 | 4 | 37 | < 2.2e-16 |
| 24 | Area+AP+AMT+NDVI | 4 | 433.4599 | 0.9072 | 0.8972 | 90.46 | 4 a | 37 | < 2.2e-16 |
| 18 | AP+AMT+NDVI | 3 | 433.6154 | 0.9023 | 0.8946 | 117 | 3 | 38 | < 2.2e-16 |
| 10 | NDVI+AMT | 2 | 434.098 | 0.8964 | 0.8911 | 168.7 | 2 | 39 | < 2.2e-16 |
| 44 | TAR+ Area+AP+AMT+NDVI+ EVI | 6 | 434.3516 | 0.9138 | 0.8991 | 61.88 | 6 | 35 | < 2.2e-16 |
| 36 | TAR+AMT+NDVI+ EVI | 4 | 434.8625 | 0.9041 | 0.8937 | 87.18 | 4 | 37 | < 2.2e-16 |
| 42 | TAR+AP+AMT+NDVI+ EVI | 5 | 434.9315 | 0.9084 | 0.8957 | 71.39 | 5 | 36 | < 2.2e-16 |
| 43 | Area+AP+AMT+NDVI+ EVI | 5 | 435.1864 | 0.9078 | 0.895 | 70.92 | 5 | 36 | < 2.2e-16 |
| 37 | AP+AMT+NDVI+ EVI | 4 | 435.6154 | 0.9023 | 0.8918 | 85.47 | 4 | 37 | < 2.2e-16 |
| 53 | NDVI + AMT+EVI | 3 | 436.098 | 0.8964 | 0.8882 | 109.6 | 3 | 38 | < 2.2e-16 |
| 14 | TAR+ AP+NDVI | 3 | 440.8884 | 0.8839 | 0.8747 | 96.42 | 3 | 38 | < 2.2e-16 |
| 21 | TAR+ Area+AP+NDVI | 4 | 441.1026 | 0.8887 | 0.8767 | 73.87 | 4 | 37 | < 2.2e-16 |
| 19 | Area+AP+NDVI | 3 | 442.0127 | 0.8807 | 0.8713 | 93.54 | 3 | 38 | < 2.2e-16 |
| 8 | AP+NDVI | 2 | 442.103 | 0.8746 | 0.8682 | 136.1 | 2 | 39 | < 2.2e-16 |
| 33 | TAR+ AP+NDVI+EVI | 4 | 442.341 | 0.8854 | .873 | 71.45 | 4 | 37 | < 2.2e-16 |
| 40 | TAR+ Area+AP+NDVI+ EVI | 5 | 443.0556 | 0.8888 | 0.8734 | 57.57 | 5 | 36 | 3.61e-16 |
| 55 | AP+NDVI+EVI | 3 | 443.4187 | 0.8767 | 0.8669 | 90.04 | 3 | 38 | < 2.2e-16 |
| 38 | Area+AP+NDVI+ EVI | 4 | 443.9446 | 0.8809 | 0.8681 | 68.43 | 4 | 37 | < 2.2e-16 |
| 32 | TAR+Area+AMT+EVI | 4 | 448.5686 | 0.8671 | 0.8527 | 60.33 | 4 | 37 | 1.044e-15 |
| 47 | AMT+Area+EVI | 3 | 448.7123 | 0.8601 | 0.8491 | 77.88 | 3 | 38 | 2.751e-16 |
| 41 | TAR+ Area+AP+AMT+ EVI | 5 | 450.43 | 0.8675 | 0.8491 | 47.14 | 5 | 36 | 8.227e-15 |
| 39 | AMT+ Area+AP+ EVI | 4 | 450.6557 | 0.8603 | 0.8452 | 56.96 | 4 | 37 | 2.599e-15 |
| 30 | TAR+Area+AP+EVI | 4 | 452.6763 | 0.8534 | 0.8376 | 53.85 | 4 | 37 | 6.282e-15 |
| 28 | AP+EVI | 2 | 452.8077 | 0.8383 | 0.83 | 101.1 | 2 | 39 | 3.734e-16 |
| 51 | TAR+AP+EVI | 3 | 452.819 | 0.8457 | 0.8336 | 69.44 | 3 | 38 | 1.75e-15 |
| 48 | AP + Area +EVI | 3 | 453.0781 | 0.8448 | 0.8325 | 68.94 | 3 | 38 | 1.966e-15 |
| 34 | TAR+ AP+AMT+EVI | 4 | 454.7974 | 0.8458 | 0.8291 | 50.74 | 4 | 37 | 1.586e-14 |
| 54 | AP + AMT +EVI | 3 | 454.8076 | 0.8383 | 0.8255 | 65.65 | 3 | 38 | 4.284e-15 |
| 50 | TAR+AMT+EVI | 3 | 459.3424 | 0.8198 | 0.8056 | 57.63 | 3 | 38 | 3.298e-14 |
| 45 | AMT+EVI | 2 | 460.2803 | 0.8068 | 0.7968 | 81.41 | 2 | 39 | 1.199e-14 |
| 29 | TAR+EVI | 2 | 473.1753 | 0.7373 | 0.7238 | 54.73 | 2 | 39 | 4.776e-12 |
| 46 | TAR+Area +EVI | 3 | 474.2713 | 0.7429 | 0.7226 | 36.6 | 3 | 38 | 2.699e-11 |
| 52 | TAR+NDVI+EVI | 3 | 475.1746 | 0.7373 | 0.7166 | 35.55 | 3 | 38 | 4.047e-11 |
| 31 | TAR+Area+NDVI+EVI | 4 | 475.8309 | 0.7456 | 0.7181 | 27.11 | 4 | 37 | 1.488e-10 |
| 35 | TAR+ Area+ NDVI+ EVI | 4 | 475.8309 | 0.7456 | 0.7181 | 27.11 | 4 | 37 | 1.488e-10 |
| 6 | TAR+NDVI | 2 | 481.6929 | 0.6783 | 0.6618 | 41.11 | 2 | 39 | 2.492e-10 |
| 12 | TAR+Area+NDVI | 3 | 483.6222 | 0.6788 | 0.6534 | 26.77 | 3 | 38 | 1.779e-09 |
| 16 | TAR+ Area+ NDVI | 3 | 483.6222 | 0.6788 | .6534 | 26.77 | 3 | 38 | 1.779e-09 |
| 27 | NDVI+EVI | 2 | 485.0633 | 0.6514 | 0.6335 | 36.43 | 2 | 39 | 1.192e-09 |
| 49 | NDVI+Area+EVI | 3 | 485.1937 | 0.6665 | 0.6402 | 25.32 | 3 | 38 | 3.59e-09 |
| 26 | Area+EVI | 2 | 486.1567 | 0.6422 | 0.6238 | 35 | 2 | 39 | 1.98e-09 |
| 15 | TAR+ AP+AMT | 3 | 489.8451 | 0.6275 | 0.5981 | 21.34 | 3 | 38 | 2.863e-08 |
| 9 | AP+AMT | 2 | 490.5544 | 0.6027 | 0.5823 | 29.58 | 2 | 39 | 1.525e-08 |
| 22 | TAR+ Area+AP+AMT | 4 | 491.0469 | 0.6345 | 0.595 | 16.06 | 4 | 37 | 1.043e-07 |
| 20 | AMT+ Area+AP | 3 | 491.8238 | 0.6095 | 0.5787 | 19.77 | 3 | 38 | 6.915e-08 |
| 11 | TAR+Area+AP | 3 | 492.4979 | 0.6032 | 0.5719 | 19.26 | 3 | 38 | 9.336e-08 |
| 2 | AP+Area | 2 | 493.1735 | 0.5771 | 0.5554 | 26.61 | 2 | 39 | 5.146e-08 |
| 13 | TAR+Area+AMT | 3 | 496.4403 | 0.5642 | 0.5298 | 16.4 | 3 | 38 | 5.387e-07 |
| 3 | NDVI+Area | 2 | 498.4506 | 0.5205 | 0.4959 | 21.17 | 2 | 39 | 5.964e-07 |
| 4 | AMT+Area | 2 | 498.5775 | 0.519 | 0.4944 | 21.04 | 2 | 39 | 6.326e-07 |
| 1 | TAR+Area | 2 | 499.121 | 0.5128 | 0.48 | 20.52 | 2 | 39 | 8.141e-07 |
| 7 | TAR+AMT | 2 | 520.4715 | 0.19 | 0.1484 | 4.573 | 2 | 39 | 0.01643 |
| 5 | TAR+AP | 2 | 521.3691 | 0.1725 | .13 | 4.064 | 2 | 39 | 0.02493 |

**Table S11** Multiple OLS regression for Wet side passerine birds from all combinations. Parameters of each multiple regressions that are significant at *P* < 0.05 are hued with bright green .It is sorted ascendingly by AIC values. TAR, Mean Temperature Annual Range; AMT, Annual Mena Temperature; AP, Annual Precipitation; NDVI, Normalized Difference Vegetation Index; EVI, Enhanced Vegetation Index.

| Model | Variables | No. vars. | AIC | *R*^2^ | R_adj_^2^ | *F* | DF | | *P* |
| --- | --- | --- | --- | --- | --- | --- | --- | --- | --- |
| 56 | TAR+Area+AMT+NDVI | 4 | 366.2963 | 0.8927 | 0.8808 | 74.88 | 4 | 36 | < 2.2e-16 |
| 25 | TAR+ Area+AP+AMT+NDVI | 5 | 367.6205 | 0.8945 | 0.8794 | 59.32 | 5 | 35 | 4.239e-16 |
| 36 | TAR+AMT+NDVI+ EVI | 4 | 367.9906 | 0.8882 | 0.8758 | 71.48 | 4 | 36 | < 2.2e-16 |
| 58 | TAR+Area+AMT+NDVI+EVI | 5 | 368.1874 | 0.893 | 0.8777 | 58.41 | 5 | 35 | 5.387e-16 |
| 23 | TAR+AP+AMT+NDVI | 4 | 368.2959 | 0.8873 | 0.8748 | 70.89 | 4 | 36 | < 2.2e-16 |
| 44 | TAR+ Area+AP+AMT+NDVI+ EVI | 6 | 369.5956 | 0.8945 | 0.8759 | 48.06 | 6 | 34 | 3.433e-15 |
| 42 | TAR+AP+AMT+NDVI+ EVI | 5 | 369.835 | 0.8886 | 0.8727 | 55.84 | 5 | 35 | 1.081e-15 |
| 57 | NDVI+AMT+Area | 3 | 373.031 | 0.8672 | 0.8565 | 80.56 | 3 | 37 | 2.773e-16 |
| 18 | AP+AMT+NDVI | 3 | 374.8813 | 0.8611 | 0.8498 | 76.46 | 3 | 37 | 6.37e-16 |
| 24 | Area+AP+AMT+NDVI | 4 | 374.9531 | 0.8675 | 0.8528 | 58.91 | 4 | 36 | 2.64e-15 |
| 17 | Area+AMT+NDVI+EVI | 4 | 375.0281 | 0.8672 | 0.8525 | 58.79 | 4 | 36 | 2.728e-15 |
| 10 | NDVI+AMT | 2 | 376.009 | 0.8501 | 0.8422 | 107.7 | 2 | 38 | < 2.2e-16 |
| 37 | AP+AMT+NDVI+ EVI | 4 | 376.6605 | 0.8618 | 0.8465 | 56.14 | 4 | 36 | 5.553e-15 |
| 43 | Area+AP+AMT+NDVI+ EVI | 5 | 376.9529 | 0.8675 | 0.8485 | 45.82 | 5 | 35 | 2.18e-14 |
| 53 | NDVI + AMT+EVI | 3 | 377.8039 | 0.8508 | 0.8387 | 70.35 | 3 | 37 | 2.368e-15 |
| 14 | TAR+ AP+NDVI | 3 | 378.0039 | 0.8501 | 0.838 | 69.95 | 3 | 37 | 2.591e-15 |
| 21 | TAR+ Area+AP+NDVI | 4 | 378.4802 | 0.8556 | 0.8395 | 53.32 | 4 | 36 | 1.226e-14 |
| 8 | AP+NDVI | 2 | 379.7519 | 0.8358 | 0.8271 | 96.68 | 2 | 38 | 1.242e-15 |
| 33 | TAR+ AP+NDVI+EVI | 4 | 379.9574 | 0.8503 | 0.8336 | 51.11 | 4 | 36 | 2.331e-14 |
| 40 | TAR+ Area+AP+NDVI+ EVI | 5 | 380.3861 | 0.8559 | 0.8353 | 41.58 | 5 | 35 | 9.261e-14 |
| 19 | Area+AP+NDVI | 3 | 380.9768 | 0.8388 | 0.8258 | 64.19 | 3 | 37 | 9.846e-15 |
| 55 | AP+NDVI+EVI | 3 | 381.7167 | 0.8359 | 0.8226 | 62.82 | 3 | 37 | 1.373e-14 |
| 38 | Area+AP+NDVI+ EVI | 4 | 382.9451 | 0.839 | 0.8211 | 46.89 | 4 | 36 | 8.546e-14 |
| 32 | TAR+Area+AMT+EVI | 4 | 385.3859 | 0.8291 | 0.8101 | 43.66 | 4 | 36 | 2.468e-13 |
| 47 | AMT+Area+EVI | 3 | 386.824 | 0.8141 | 0.7991 | 54.02 | 3 | 37 | 1.358e-13 |
| 41 | TAR+ Area+AP+AMT+ EVI | 5 | 387.3806 | 0.8291 | 0.8047 | 33.96 | 5 | 35 | 1.753e-12 |
| 39 | AMT+ Area+AP+ EVI | 4 | 388.5757 | 0.8153 | 0.7947 | 39.71 | 4 | 36 | 9.856e-13 |
| 51 | TAR+AP+EVI | 3 | 388.7536 | 0.8052 | 0.7894 | 50.97 | 3 | 37 | 3.228e-13 |
| 30 | TAR+Area+AP+EVI | 4 | 388.9049 | 0.8138 | 0.7931 | 39.33 | 4 | 36 | 1.137e-12 |
| 48 | AP + Area +EVI | 3 | 389.1245 | 0.8034 | 0.7875 | 50.4 | 3 | 37 | 3.812e-13 |
| 28 | AP+EVI | 2 | 389.2065 | 0.7932 | 0.7823 | 72.86 | 2 | 38 | 9.933e-14 |
| 34 | TAR+ AP+AMT+EVI | 4 | 390.7395 | 0.8052 | 0.7836 | 37.21 | 4 | 36 | 2.519e-12 |
| 54 | AP + AMT +EVI | 3 | 391.095 | 0.7937 | 0.777 | 47.46 | 3 | 37 | 9.221e-13 |
| 50 | TAR+AMT+EVI | 3 | 395.1868 | 0.7721 | 0.7536 | 41.78 | 3 | 37 | 5.768e-12 |
| 45 | AMT+EVI | 2 | 399.7514 | 0.7325 | 0.7184 | 52.03 | 2 | 38 | 1.316e-11 |
| 9 | AP+AMT | 2 | 408.3656 | 0.67 | 0.6526 | 38.57 | 2 | 38 | 7.128e-10 |
| 20 | AMT+ Area+AP | 3 | 409.5043 | 0.6768 | 0.6506 | 25.83 | 3 | 37 | 3.468e-09 |
| 2 | AP+Area | 2 | 410.0695 | 0.6559 | 0.6378 | 36.22 | 2 | 38 | 1.57e-09 |
| 15 | TAR+ AP+AMT | 3 | 410.2633 | 0.6708 | 0.6441 | 25.13 | 3 | 37 | 4.864e-09 |
| 29 | TAR+EVI | 2 | 410.8556 | 0.6493 | 0.6308 | 35.18 | 2 | 38 | 2.26e-09 |
| 22 | TAR+ Area+AP+AMT | 4 | 411.382 | 0.6778 | 0.642 | 18.93 | 4 | 36 | 1.851e-08 |
| 11 | TAR+Area+AP | 3 | 411.9568 | 0.6569 | .6291 | 23.61 | 3 | 37 | 1.034e-08 |
| 46 | TAR+Area +EVI | 3 | 412.7054 | 0.6506 | 0.6222 | 22.96 | 3 | 37 | 1.443e-08 |
| 52 | TAR+NDVI+EVI | 3 | 412.8537 | 0.6493 | 0.6209 | 22.83 | 3 | 37 | 1.542e-08 |
| 31 | TAR+Area+NDVI+EVI | 4 | 414.6196 | 0.6513 | 0.6126 | 16.81 | 4 | 36 | 7.392e-08 |
| 35 | TAR+ Area+ NDVI+ EVI | 4 | 414.6196 | 0.6513 | 0.6126 | 16.81 | 4 | 36 | 7.392e-08 |
| 6 | TAR+NDVI | 2 | 417.6248 | 0.5863 | 0.5646 | 26.93 | 2 | 38 | 5.206e-08 |
| 4 | AMT+Area | 2 | 418.7364 | 0.575 | 0.5526 | 25.7 | 2 | 38 | 8.714e-08 |
| 12 | TAR+Area+NDVI | 3 | 419.4764 | 0.5878 | 0.5544 | 17.59 | 3 | 37 | 2.924e-07 |
| 16 | TAR+ Area+ NDVI | 3 | 419.4764 | 0.5878 | 0.5544 | 17.59 | 3 | 37 | 2.924e-07 |
| 13 | TAR+Area+AMT | 3 | 419.7285 | 0.5853 | 0.5517 | 17.41 | 3 | 37 | 3.27e-07 |
| 49 | NDVI+Area+EVI | 3 | 427.6775 | 0.4966 | 0.4557 | 12.16 | 3 | 37 | 1.096e-05 |
| 27 | NDVI+EVI | 2 | 428.3896 | 0.4621 | 0.4338 | 16.32 | 2 | 38 | 7.638e-06 |
| 1 | TAR+Area | 2 | 430.6331 | 0.4319 | 0.402 | 14.44 | 2 | 38 | 2.16e-05 |
| 26 | Area+EVI | 2 | 430.9396 | 0.4276 | .3975 | 14.19 | 2 | 38 | 2.49e-05 |
| 5 | TAR+AP | 2 | 437.0084 | 0.3363, | 0.3014 | 9.627 | 2 | 38 | 0.0004146 |
| 3 | NDVI+Area | 2 | 439.3452 | 0.2974 | 0.2604 | 8.041 | 2 | 38 | 0.001224 |
| 7 | TAR+AMT | 2 | 447.903 | 0.1343 | 0.08871 | 2.947 | 2 | 38 | 0.0646 |

**Table S12** Multiple OLS regression for Wet side non-passerine birds from all combinations. Parameters of each multiple regressions that are significant at *P* < 0.05 are hued with green. It is sorted ascendingly by AIC values. TAR, Mean Temperature Annual Range; AMT, Annual Mena Temperature; AP, Annual Precipitation; NDVI, Normalized Difference Vegetation Index; EVI, Enhanced Vegetation Index.

| Model | Variables | No. vars. | AIC | *R*^2^ | R_adj_^2^ | *F* | DF | | *P* |
| --- | --- | --- | --- | --- | --- | --- | --- | --- | --- |
| 56 | TAR+Area+AMT+NDVI | 4 | 366.7029 | 0.9064 | 0.8963 | 89.56 | 4 | 37 | < 2.2e-16 |
| 25 | TAR+ Area+AP+AMT+NDVI | 5 | 368.2781 | 0.9073 | 0.8945 | 70.49 | 5 | 36 | < 2.2e-16 |
| 23 | TAR+AP+AMT+NDVI | 4 | 368.3448 | 0.9027 | 0.8921 | 85.77 | 4 | 37 | < 2.2e-16 |
| 36 | TAR+AMT+NDVI+ EVI | 4 | 368.5622 | 0.9021 | 0.8916 | 85.28 | 4 | 37 | < 2.2e-16 |
| 58 | TAR+Area+AMT+NDVI+EVI | 5 | 368.683 | 0.9064 | 0.8934 | 69.75 | 5 | 36 | < 2.2e-16 |
| 42 | TAR+AP+AMT+NDVI+ EVI | 5 | 370.1432 | 0.9031 | 0.8897 | 67.12 | 5 | 36 | < 2.2e-16 |
| 44 | TAR+ Area+AP+AMT+NDVI+ EVI | 6 | 370.2774 | 0.9073 | 0.8914 | 57.11 | 6 | 35 | < 2.2e-16 |
| 57 | NDVI+AMT+Area | 3 | 371.6297 | 0.8896 | 0.8809 | 102.1 | 3 | 38 | < 2.2e-16 |
| 10 | NDVI+AMT | 2 | 372.0916 | 0.8829 | 0.8769 | 147.1 | 2 | 39 | < 2.2e-16 |
| 18 | AP+AMT+NDVI | 3 | 373.1923 | 0.8854 | 0.8764 | 97.87 | 3 | 38 | < 2.2e-16 |
| 24 | Area+AP+AMT+NDVI | 4 | 373.509 | 0.8899 | 0.878 | 74.77 | 4 | 37 | < 2.2e-16 |
| 17 | Area+AMT+NDVI+EVI | 4 | 373.6293 | 0.8896 | 0.8777 | 74.53 | 4 | 37 | < 2.2e-16 |
| 53 | NDVI + AMT+EVI | 3 | 373.992 | 0.8832 | 0.874 | 95.79 | 3 | 38 | < 2.2e-16 |
| 37 | AP+AMT+NDVI+ EVI | 4 | 375.089 | 0.8857 | 0.8733 | 71.67 | 4 | 37 | < 2.2e-16 |
| 43 | Area+AP+AMT+NDVI+ EVI | 5 | 375.496 | 0.8899 | 0.8747 | 58.22 | 5 | 36 | 3.02e-16 |
| 21 | TAR+ Area+AP+NDVI | 4 | 379.2785 | 0.8737 | 0.86 | 63.99 | 4 | 37 | 4.077e-16 |
| 14 | TAR+ AP+NDVI | 3 | 380.6267 | 0.8632 | 0.8524 | 79.94 | 3 | 38 | < 2.2e-16 |
| 40 | TAR+ Area+AP+NDVI+ EVI | 5 | 381.237 | 0.8738 | 0.8563 | 49.87 | 5 | 36 | 3.446e-15 |
| 33 | TAR+ AP+NDVI+EVI | 4 | 382.3124 | 0.8642 | 0.8496 | 58.89 | 4 | 37 | 1.536e-15 |
| 19 | Area+AP+NDVI | 3 | 383.7345 | 0.8527 | 0.8411 | 73.34 | 3 | 38 | 7.284e-16 |
| 8 | AP+NDVI | 2 | 385.412 | 0.8392 | 0.831 | 101.8 | 2 | 39 | 3.315e-16 |
| 38 | Area+AP+NDVI+ EVI | 4 | 385.7216 | 0.8528 | 0.8368 | 53.57 | 4 | 37 | 6.808e-15 |
| 32 | TAR+Area+AMT+EVI | 4 | 386.769 | 0.849 | 0.8327 | 52.03 | 4 | 37 | 1.075e-14 |
| 55 | AP+NDVI+EVI | 3 | 386.9477 | 0.841 | 0.8285 | 67 | 3 | 38 | 3.096e-15 |
| 41 | TAR+ Area+AP+AMT+ EVI | 5 | 388.3924 | 0.8504 | 0.8296 | 40.93 | 5 | 36 | 7.12e-14 |
| 47 | AMT+Area+EVI | 3 | 389.7167 | 0.8302 | 0.8168 | 61.92 | 3 | 38 | 1.077e-14 |
| 51 | TAR+AP+EVI | 3 | 390.1959 | 0.8282 | 0.8147 | 61.07 | 3 | 38 | 1.336e-14 |
| 30 | TAR+Area+AP+EVI | 4 | 391.5277 | 0.8309 | 0.8127 | 45.46 | 4 | 37 | 8.572e-14 |
| 39 | AMT+ Area+AP+ EVI | 4 | 391.5635 | 0.8308 | 0.8125 | 45.42 | 4 | 37 | 8.707e-14 |
| 34 | TAR+ AP+AMT+EVI | 4 | 391.9415 | 0.8293 | 0.8108 | 44.93 | 4 | 37 | 1.027e-13 |
| 28 | AP+EVI | 2 | 393.1041 | 0.8069 | 0.797 | 81.5 | 2 | 39 | 1.179e-14 |
| 50 | TAR+AMT+EVI | 3 | 394.0477 | 0.8117 | 0.7969 | 54.61 | 3 | 38 | 7.559e-14 |
| 54 | AP + AMT +EVI | 3 | 394.6308 | 0.8091 | 0.794 | 53.68 | 3 | 38 | 9.826e-14 |
| 48 | AP + Area +EVI | 3 | 394.7396 | 0.8086 | 0.7935 | 53.51 | 3 | 38 | 1.032e-13 |
| 45 | AMT+EVI | 2 | 397.744 | 0.7844 | 0.7733 | 70.94 | 2 | 39 | 1.016e-13 |
| 29 | TAR+EVI | 2 | 410.3548 | 0.7089 | 0.6939 | 47.48 | 2 | 39 | 3.547e-11 |
| 46 | TAR+Area +EVI | 3 | 412.1386 | 0.7104 | 0.6875 | 31.07 | 3 | 38 | 2.545e-10 |
| 52 | TAR+NDVI+EVI | 3 | 412.3251 | 0.7091 | 0.6861 | 30.87 | 3 | 38 | 2.766e-10 |
| 31 | TAR+Area+NDVI+EVI | 4 | 413.8339 | 0.7125 | 0.6814 | 22.92 | 4 | 37 | 1.373e-09 |
| 35 | TAR+ Area+ NDVI+ EVI | 4 | 413.8339 | 0.7125 | 0.6814 | 22.92 | 4 | 37 | 1.373e-09 |
| 6 | TAR+NDVI | 2 | 417.9149 | 0.6514 | 0.6336 | 36.45 | 2 | 39 | 1.186e-09 |
| 12 | TAR+Area+NDVI | 3 | 419.908 | 0.6515 | 0.624 | 23.68 | 3 | 38 | 8.216e-09 |
| 16 | TAR+ Area+ NDVI | 3 | 419.908 | 0.6515 | 0.624 | 23.68 | 3 | 38 | 8.216e-09 |
| 15 | TAR+ AP+AMT | 3 | 424.6245 | 0.6101 | 0.5793 | 19.82 | 3 | 38 | 6.731e-08 |
| 22 | TAR+ Area+AP+AMT | 4 | 425.8988 | 0.6168 | 0.5753 | 14.89 | 4 | 37 | 2.442e-07 |
| 27 | NDVI+EVI | 2 | 426.0501 | 0.577 | 0.5553 | 26.59 | 2 | 39 | 5.183e-08 |
| 11 | TAR+Area+AP | 3 | 426.3717 | 0.5935 | 0.5614 | 18.5 | 3 | 38 | 1.465e-07 |
| 49 | NDVI+Area+EVI | 3 | 427.0757 | 0.5867 | 0.554 | 17.98 | 3 | 38 | 2.004e-07 |
| 9 | AP+AMT | 2 | 427.2624 | 0.5646 | 0.5422 | 25.28 | 2 | 39 | 9.099e-08 |
| 26 | Area+EVI | 2 | 428.1163 | 0.5556 | 0.5328 | 24.38 | 2 | 39 | 1.353e-07 |
| 20 | AMT+ Area+AP | 3 | 428.6339 | 0.571 | 0.5372 | 16.86 | 3 | 38 | 4.005e-07 |
| 2 | AP+Area | 2 | 428.971 | 0.5465 | 0.5232 | 23.5 | 2 | 39 | 2.012e-07 |
| 13 | TAR+Area+AMT | 3 | 430.1135 | 0.5557 | 0.5206 | 15.84 | 3 | 38 | 7.724e-07 |
| 1 | TAR+Area | 2 | 433.6193 | 0.4934 | 0.4674 | 18.99 | 2 | 39 | 1.741e-06 |
| 4 | AMT+Area | 2 | 434.3089 | 0.485 | 0.4586 | 18.37 | 2 | 39 | 2.398e-06 |
| 3 | NDVI+Area | 2 | 438.674 | 0.4286 | 0.3993 | 14.63 | 2 | 39 | 1.82e-05 |
| 5 | TAR+AP | 2 | 450.4141 | 0.2444 | 0.2056 | 6.306 | 2 | 39 | 0.004239 |
| 7 | TAR+AMT | 2 | 451.622 | 0.2223 | 0.1824 | 5.574 | 2 | 39 | 0.007427 |

**Table S13** Multiple OLS regression for western slope all birds from all combinations. Parameters of each multiple regressions that are significant at *P* < 0.05 are hued with bright green. It is sorted ascendingly by AIC values. TAR, Mean Temperature Annual Range; AMT, Annual Mena Temperature; AP, Annual Precipitation; NDVI, Normalized Difference Vegetation Index; EVI, Enhanced Vegetation Index.

| Model | Variables | No. vars. | AIC | *R*^2^ | R_adj_^2^ | *F* | DF | | *P* |
| --- | --- | --- | --- | --- | --- | --- | --- | --- | --- |
| 33 | TAR+ AP+NDVI+EVI | 4 | 413.8538 | 0.9138 | 0.904 | 92.8 | 4 | 35 | < 2.2e-16 |
| 14 | TAR+ AP+NDVI | 3 | 413.8816 | 0.9094 | 0.9018 | 120.4 | 3 | 36 | < 2.2e-16 |
| 23 | TAR+AP+AMT+NDVI | 4 | 414.5348 | 0.9124 | 0.9023 | 91.08 | 4 | 35 | < 2.2e-16 |
| 42 | TAR+AP+AMT+NDVI+ EVI | 5 | 414.8967 | 0.9159 | 0.9035 | 74.03 | 5 | 34 | < 2.2e-16 |
| 21 | TAR+ Area+AP+NDVI | 4 | 415.4588 | 0.9103 | 0.9001 | 88.8 | 4 | 35 | < 2.2e-16 |
| 40 | TAR+ Area+AP+NDVI+ EVI | 5 | 415.7071 | 0.9141 | 0.9015 | 72.41 | 5 | 34 | < 2.2e-16 |
| 25 | TAR+ Area+AP+AMT+NDVI | 5 | 416.5247 | 0.9124 | 0.8995 | 70.8 | 5 | 34 | < 2.2e-16 |
| 44 | TAR+ Area+AP+AMT+NDVI+ EVI | 6 | 416.8165 | 0.916 | 0.9008 | 60.01 | 6 | 33 | 2.426e-16 |
| 36 | TAR+AMT+NDVI+ EVI | 4 | 417.0797 | 0.9066 | 0.8959 | 84.93 | 4 | 35 | < 2.2e-16 |
| 56 | TAR+Area+AMT+NDVI | 4 | 417.69 | 0.9052 | 0.8943 | 83.51 | 4 | 35 | < 2.2e-16 |
| 58 | TAR+Area+AMT+NDVI+EVI | 5 | 417.9826 | 0.9091 | 0.8958 | 68.03 | 5 | 34 | < 2.2e-16 |
| 8 | AP+NDVI | 2 | 418.6399 | 0.8927 | 0.8869 | 153.9 | 2 | 37 | < 2.2e-16 |
| 55 | AP+NDVI+EVI | 3 | 419.1144 | 0.8967 | 0.8881 | 104.1 | 3 | 36 | < 2.2e-16 |
| 18 | AP+AMT+NDVI | 3 | 419.6763 | 0.8952 | 0.8865 | 102.5 | 3 | 36 | < 2.2e-16 |
| 19 | Area+AP+NDVI | 3 | 420.4183 | 0.8933 | 0.8844 | 100.4 | 3 | 36 | < 2.2e-16 |
| 37 | AP+AMT+NDVI+ EVI | 4 | 420.4468 | 0.8984 | 0.8868 | 77.37 | 4 | 35 | < 2.2e-16 |
| 6 | TAR+NDVI | 2 | 420.8018 | 0.8867 | 0.8806 | 144.8 | 2 | 37 | < 2.2e-16 |
| 52 | TAR+NDVI+EVI | 3 | 421.0283 | 0.8916 | 0.8826 | 98.72 | 3 | 36 | < 2.2e-16 |
| 38 | Area+AP+NDVI+ EVI | 4 | 421.0606 | 0.8968 | 0.885 | 76.05 | 4 | 35 | < 2.2e-16 |
| 24 | Area+AP+AMT+NDVI | 4 | 421.6404 | 0.8953 | 0.8834 | 74.83 | 4 | 35 | < 2.2e-16 |
| 12 | TAR+Area+NDVI | 3 | 421.7908 | 0.8895 | 0.8803 | 96.63 | 3 | 36 | < 2.2e-16 |
| 16 | TAR+ Area+ NDVI | 3 | 421.7908 | 0.8895 | 0.8803 | 96.63 | 3 | 36 | < 2.2e-16 |
| 43 | Area+AP+AMT+NDVI+ EVI | 5 | 422.324 | 0.8987 | 0.8838 | 60.33 | 5 | 34 | 6.277e-16 |
| 31 | TAR+Area+NDVI+EVI | 4 | 422.4383 | 0.8932 | 0.881 | 73.18 | 4 | 35 | < 2.2e-16 |
| 35 | TAR+ Area+ NDVI+ EVI | 4 | 422.4383 | 0.8932 | 0.881 | 73.18 | 4 | 35 | < 2.2e-16 |
| 15 | TAR+ AP+AMT | 3 | 423.6179 | 0.8844 | 0.8747 | 91.78 | 3 | 36 | < 2.2e-16 |
| 57 | NDVI+AMT+Area | 3 | 424.2935 | 0.8824 | 0.8726 | 90.04 | 3 | 36 | < 2.2e-16 |
| 10 | NDVI+AMT | 2 | 424.5607 | 0.8755 | 0.8688 | 130.1 | 2 | 37 | < 2.2e-16 |
| 53 | NDVI + AMT+EVI | 3 | 424.6369 | 0.8814 | 0.8715 | 89.17 | 3 | 36 | < 2.2e-16 |
| 17 | Area+AMT+NDVI+EVI | 4 | 425.0015 | 0.8861 | 0.8731 | 68.1 | 4 | 35 | 5.062e-16 |
| 22 | TAR+ Area+AP+AMT | 4 | 425.3514 | 0.8851 | 0.872 | 67.43 | 4 | 35 | 5.893e-16 |
| 34 | TAR+ AP+AMT+EVI | 4 | 425.5922 | 0.8844 | 0.8712 | 66.97 | 4 | 35 | 6.543e-16 |
| 41 | TAR+ Area+AP+AMT+ EVI | 5 | 427.3465 | 0.8852 | 0.8683 | 52.41 | 5 | 34 | 5.194e-15 |
| 9 | AP+AMT | 2 | 435.553 | 0.8362 | 0.8273 | 94.43 | 2 | 37 | 2.923e-15 |
| 54 | AP + AMT +EVI | 3 | 436.9256 | 0.8387 | 0.8253 | 62.41 | 3 | 36 | 2.449e-14 |
| 20 | AMT+ Area+AP | 3 | 436.9315 | 0.8387 | 0.8253 | 62.4 | 3 | 36 | 2.456e-14 |
| 39 | AMT+ Area+AP+ EVI | 4 | 438.5013 | 0.8404 | 0.8222 | 46.08 | 4 | 35 | 1.769e-13 |
| 13 | TAR+Area+AMT | 3 | 439.0878 | 0.8298 | 0.8156 | 58.49 | 3 | 36 | 6.448e-14 |
| 32 | TAR+Area+AMT+EVI | 4 | 440.2245 | 0.8334 | 0.8144 | 43.77 | 4 | 35 | .731e-13 |
| 46 | TAR+Area +EVI | 3 | 440.5036 | 0.8236 | 0.8089 | 56.04 | 3 | 36 | 1.215e-13 |
| 1 | TAR+Area | 2 | 441.4913 | 0.81 | 0.7997 | 78.85 | 2 | 37 | 4.557e-14 |
| 30 | TAR+Area+AP+EVI | 4 | 442.3229 | 0.8244 | 0.8044 | 41.09 | 4 | 35 | 9.249e-13 |
| 11 | TAR+Area+AP | 3 | 442.4624 | 0.8148 | 0.7994 | 52.79 | 3 | 36 | 2.919e-13 |
| 7 | TAR+AMT | 2 | 445.1936 | 0.7915 | 0.7803 | 70.24 | 2 | 37 | 2.525e-13 |
| 50 | TAR+AMT+EVI | 3 | 446.5268 | 0.795 | 0.7779 | 46.53 | 3 | 36 | 1.797e-12 |
| 3 | NDVI+Area | 2 | 447.1057 | 0.7813 | 0.7695 | 66.1 | 2 | 37 | 6.115e-13 |
| 27 | NDVI+EVI | 2 | 447.5198 | 0.779 | 0.7671 | 65.23 | 2 | 37 | 7.405e-13 |
| 49 | NDVI+Area+EVI | 3 | 448.6607 | 0.7837 | 0.7657 | 43.49 | 3 | 36 | 4.663e-12 |
| 51 | TAR+AP+EVI | 3 | 457.2126 | 0.7322 | 0.7099 | : 32.81 | 3 | 36 | 2.119e-10 |
| 29 | TAR+EVI | 2 | 458.7527 | 0.7074 | 0.6916 | 44.73 | 2 | 37 | 1.336e-10 |
| 5 | TAR+AP | 2 | 459.1407 | 0.7046 | 0.6886 | 44.12 | 2 | 37 | 1.599e-10 |
| 48 | AP + Area +EVI | 3 | 460.9609 | 0.7059 | 0.6814 | 28.8 | 3 | 36 | 1.126e-09 |
| 26 | Area+EVI | 2 | 464.653 | 0.6609 | 0.6426 | 36.06 | 2 | 37 | 2.046e-09 |
| 47 | AMT+Area+EVI | 3 | 465.475 | 0.6707 | 0.6433 | : 24.45 | 3 | 36 | 8.381e-09 |
| 2 | AP+Area | 2 | 466.546 | 0.6445 | 0.6253 | 33.54 | 2 | 37 | 4.911e-09 |
| 4 | AMT+Area | 2 | 469.4866 | 0.6174 | 0.5967 | 29.85 | 2 | 37 | 1.913e-08 |
| 45 | AMT+EVI | 2 | 486.7425 | 0.4109 | 0.3791 | 12.91 | 2 | 37 | 5.596e-05 |
| 28 | AP+EVI | 2 | 488.0257 | 0.3917 | 0.3589 | 11.91 | 2 | 37 | 0.0001013 |

**Table S14** Multiple OLS regression for western slope passerine birds from all combinations. Parameters of each multiple regressions that are significant at *P* < 0.05 are hued with bright green. It is sorted ascendingly by AIC values. TAR, Mean Temperature Annual Range; AMT, Annual Mena Temperature; AP, Annual Precipitation; NDVI, Normalized Difference Vegetation Index; EVI, Enhanced Vegetation Index.

| Model | Variables | No. vars. | AIC | *R*^2^ | R_adj_^2^ | *F* | DF | | *P* |
| --- | --- | --- | --- | --- | --- | --- | --- | --- | --- |
| 14 | TAR+ AP+NDVI | 3 | 366.7672 | 0.8866 | 0.8771 | 93.78 | 3 | 36 | < 2.2e-16 |
| 33 | TAR+ AP+NDVI+EVI | 4 | 367.9155 | 0.8889 | 0.8763 | 70.04 | 4 | 35 | 3.279e-16 |
| 23 | TAR+AP+AMT+NDVI | 4 | 368.5512 | 0.8872 | 0.8743 | 68.8 | 4 | 35 | 4.322e-16 |
| 21 | TAR+ Area+AP+NDVI | 4 | 368.6924 | 0.8868 | 0.8738 | : 68.53 | 4 | 35 | 4.596e-16 |
| 40 | TAR+ Area+AP+NDVI+ EVI | 5 | 369.6915 | 0.8896 | 0.8733 | 54.78 | 5 | 34 | 2.686e-15 |
| 42 | TAR+AP+AMT+NDVI+ EVI | 5 | 369.8051 | 0.8893 | 0.873 | 54.6 | 5 | 34 | 2.817e-15 |
| 25 | TAR+ Area+AP+AMT+NDVI | 5 | 370.0136 | 0.8887 | 0.8723 | 54.28 | 5 | 34 | 3.075e-15 |
| 36 | TAR+AMT+NDVI+ EVI | 4 | 370.3665 | 0.8819 | 0.8684 | 65.36 | 4 | 35 | 9.511e-16 |
| 44 | TAR+ Area+AP+AMT+NDVI+ EVI | 6 | 371.0497 | 0.8913 | 0.8716 | 45.11 | 6 | 33 | 1.627e-14 |
| 56 | TAR+Area+AMT+NDVI | 4 | 371.242 | 0.8793 | 0.8655 | 63.75 | 4 | 35 | 1.391e-15 |
| 58 | TAR+Area+AMT+NDVI+EVI | 5 | 372.2542 | 0.8823 | 0.8649 | 50.95 | 5 | 34 | 7.889e-15 |
| 8 | AP+NDVI | 2 | 372.5902 | 0.8621 | 0.8546 | 115.6 | 2 | 37 | < 2.2e-16 |
| 55 | AP+NDVI+EVI | 3 | 374.0096 | 0.864 | 0.8527 | 76.26 | 3 | 36 | 1.149e-15 |
| 19 | Area+AP+NDVI | 3 | 374.4446 | 0.8626 | 0.8511 | 75.31 | 3 | 36 | 1.397e-15 |
| 18 | AP+AMT+NDVI | 3 | 374.4786 | 0.8624 | 0.851 | 75.23 | 3 | 36 | 1.418e-15 |
| 38 | Area+AP+NDVI+ EVI | 4 | 375.706 | 0.8651 | 0.8496 | 56.1 | 4 | 35 | 9.657e-15 |
| 24 | Area+AP+AMT+NDVI | 4 | 375.8912 | 0.8644 | 0.849 | 55.8 | 4 | 35 | 1.047e-14 |
| 6 | TAR+NDVI | 2 | 375.9593 | 0.8499 | 0.8418 | 104.8 | 2 | 37 | 5.773e-16 |
| 37 | AP+AMT+NDVI+ EVI | 4 | 375.9603 | 0.8642 | 0.8487 | 55.69 | 4 | 35 | 1.078e-14 |
| 15 | TAR+ AP+AMT | 3 | 376.5069 | 0.8553 | 0.8432 | 70.92 | 3 | 36 | 3.519e-15 |
| 43 | Area+AP+AMT+NDVI+ EVI | 5 | 377.1841 | 0.8668 | 0.8472 | 44.26 | 5 | 34 | 6.255e-14 |
| 52 | TAR+NDVI+EVI | 3 | 377.2061 | 0.8527 | 0.8405 | 69.48 | 3 | 36 | 4.813e-15 |
| 10 | NDVI+AMT | 2 | 377.3356 | 0.8447 | 0.8363 | 100.6 | 2 | 37 | 1.091e-15 |
| 22 | TAR+ Area+AP+AMT | 4 | 377.3932 | 0.8593 | 0.8432 | 53.42 | 4 | 35 | 2.008e-14 |
| 12 | TAR+Area+NDVI | 3 | 377.9038 | 0.8501 | 0.8376 | 68.07 | 3 | 36 | 6.579e-15 |
| 16 | TAR+ Area+ NDVI | 3 | 377.9038 | 0.8501 | 0.8376 | 68.07 | 3 | 36 | 6.579e-15 |
| 34 | TAR+ AP+AMT+EVI | 4 | 378.3264 | 0.8559 | 0.8395 | 51.99 | 4 | 35 | 3.009e-14 |
| 53 | NDVI + AMT+EVI | 3 | 378.3549 | 0.8484 | 0.8358 | 67.18 | 3 | 36 | 8.052e-15 |
| 57 | NDVI+AMT+Area | 3 | 378.6482 | 0.8473 | 0.8346 | 66.6 | 3 | 36 | 9.182e-15 |
| 31 | TAR+Area+NDVI+EVI | 4 | 379.2021 | 0.8527 | 0.8359 | 50.67 | 4 | 35 | 4.398e-14 |
| 35 | TAR+ Area+ NDVI+ EVI | 4 | 379.2021 | 0.8527 | 0.8359 | 50.67 | 4 | 35 | 4.398e-14 |
| 41 | TAR+ Area+AP+AMT+ EVI | 5 | 379.3332 | 0.8595 | 0.8388 | 41.59 | 5 | 34 | 1.541e-13 |
| 17 | Area+AMT+NDVI+EVI | 4 | 379.9305 | 0.85 | 0.8329 | 49.6 | 4 | 35 | 6.031e-14 |
| 9 | AP+AMT | 2 | 389.214 | 0.791 | 0.7797 | 70 | 2 | 37 | 2.653e-13 |
| 20 | AMT+ Area+AP | 3 | 389.6717 | 0.7989 | 0.7821 | 47.67 | 3 | 36 | 1.274e-12 |
| 46 | TAR+Area +EVI | 3 | 389.9052 | 0.7977 | 0.7808 | 47.32 | 3 | 36 | 1.415e-12 |
| 1 | TAR+Area | 2 | 390.1297 | 0.7861 | 0.7746 | 68 | 2 | 37 | 4.052e-13 |
| 54 | AP + AMT +EVI | 3 | 390.1637 | 0.7964 | 0.7794 | 46.94 | 3 | 36 | 1.588e-12 |
| 13 | TAR+Area+AMT | 3 | 390.6089 | 0.7941 | 0.777 | 46.28 | 3 | 36 | 1.938e-12 |
| 39 | AMT+ Area+AP+ EVI | 4 | 391.0095 | 0.8022 | 0.7796 | 35.48 | 4 | 35 | 7.276e-12 |
| 32 | TAR+Area+AMT+EVI | 4 | 391.4843 | 0.7998 | 0.7769 | 34.96 | 4 | 35 | 8.932e-12 |
| 30 | TAR+Area+AP+EVI | 4 | 391.7586 | 0.7984 | 0.7754 | 34.66 | 4 | 35 | 1.005e-11 |
| 11 | TAR+Area+AP | 3 | 392.0903 | 0.7863 | 0.7685 | 44.16 | 3 | 36 | 3.756e-12 |
| 7 | TAR+AMT | 2 | 393.6067 | 0.7667 | 0.7541 | 60.8 | 2 | 37 | 2.024e-12 |
| 50 | TAR+AMT+EVI | 3 | 394.6512 | 0.7722 | 0.7532 | 40.68 | 3 | 36 | 1.179e-11 |
| 29 | TAR+EVI | 2 | 399.2001 | 0.7317 | 0.7172 | 50.45 | 2 | 37 | 2.689e-11 |
| 51 | TAR+AP+EVI | 3 | 400.3771 | 0.7372 | 0.7153 | 33.66 | 3 | 36 | 1.518e-10 |
| 5 | TAR+AP | 2 | 402.3709 | 0.7096 | 0.6939 | 45.2 | 2 | 37 | 1.165e-10 |
| 3 | NDVI+Area | 2 | 406.1682 | 0.6806 | 0.6634 | 39.43 | 2 | 37 | 6.749e-10 |
| 27 | NDVI+EVI | 2 | 406.1938 | 0.6804 | 0.6632 | 39.39 | 2 | 37 | 6.829e-10 |
| 49 | NDVI+Area+EVI | 3 | 407.9984 | 0.682 | 0.6555 | 25.73 | 3 | 36 | 4.518e-09 |
| 48 | AP + Area +EVI | 3 | 410.9472 | 0.6577 | 0.6291 | 23.05 | 3 | 36 | 1.675e-08 |
| 2 | AP+Area | 2 | 416.8394 | 0.583 | 0.5605 | 25.86 | 2 | 37 | 9.39e-08 |
| 47 | AMT+Area+EVI | 3 | 417.239 | 0.5993 | 0.566 | 17.95 | 3 | 36 | 2.723e-07 |
| 26 | Area+EVI | 2 | 418.4163 | 0.5662 | 0.5428 | 24.15 | 2 | 37 | 1.947e-07 |
| 4 | AMT+Area | 2 | 421.7375 | 0.5287 | 0.5032 | 20.75 | 2 | 37 | 9.047e-07 |
| 28 | AP+EVI | 2 | 431.7934 | 0.394 | 0.3612 | 12.03 | 2 | 37 | 9.47e-05 |
| 45 | AMT+EVI | 2 | 434.8608 | 0.3457 | 0.3103 | 9.772 | 2 | 37 | 0.0003912 |

**Table S15** Multiple OLS regression for western slope non-passerine birds from all combinations. Parameters of each multiple regressions that are significant at *P* < 0.05 are hued with green. It is sorted ascendingly by AIC values. TAR, Mean Temperature Annual Range; AMT, Annual Mena Temperature; AP, Annual Precipitation; NDVI, Normalized Difference Vegetation Index; EVI, Enhanced Vegetation Index.

| Model | Variables | No. vars. | AIC | *R*^2^ | R_adj_^2^ | *F* | DF | | *P* |
| --- | --- | --- | --- | --- | --- | --- | --- | --- | --- |
| 36 | TAR+AMT+NDVI+ EVI | 4 | 383.6089 | 0.8599 | 0.8443 | 55.23 | 4 | 36 | 7.154e-15 |
| 14 | TAR+ AP+NDVI | 3 | 384.0576 | 0.8512 | 0.8392 | 70.58 | 3 | 37 | 2.251e-15 |
| 8 | AP+NDVI | 2 | 384.2419 | 0.8431 | 0.8348 | 102.1 | 2 | 38 | 5.208e-16 |
| 33 | TAR+ AP+NDVI+EVI | 4 | 384.942 | 0.8552 | .8392 | 53.17 | 4 | 36 | 1.278e-14 |
| 56 | TAR+Area+AMT+NDVI | 4 | 384.9997 | 0.855 | 0.8389 | 53.08 | 4 | 36 | 1.31e-14 |
| 23 | TAR+AP+AMT+NDVI | 4 | 385.0336 | 0.8549 | 0.8388 | 53.03 | 4 | 36 | 1.33e-14 |
| 55 | AP+NDVI+EVI | 3 | 385.1848 | 0.8471 | 0.8347 | 68.33 | 3 | 37 | 3.735e-15 |
| 42 | TAR+AP+AMT+NDVI+ EVI | 5 | 385.4754 | 0.8603 | 0.8404 | 43.12 | 5 | 35 | 5.409e-14 |
| 58 | TAR+Area+AMT+NDVI+EVI | 5 | 385.4994 | 0.8602 | 0.8403 | 43.09 | 5 | 35 | 5.464e-14 |
| 10 | NDVI+AMT | 2 | 385.7617 | 0.8372 | 0.8286 | 97.69 | 2 | 38 | 1.053e-15 |
| 21 | TAR+ Area+AP+NDVI | 4 | 385.9433 | 0.8517 | 0.8352 | 51.67 | 4 | 36 | 1.976e-14 |
| 18 | AP+AMT+NDVI | 3 | 385.9707 | 0.8441 | 0.8315 | 66.8 | 3 | 37 | 5.316e-15 |
| 19 | Area+AP+NDVI | 3 | 386.0975 | 0.8437 | 0.831 | 66.55 | 3 | 37 | 5.628e-15 |
| 53 | NDVI + AMT+EVI | 3 | 386.123 | 0.8436 | 0.8309 | 66.5 | 3 | 37 | 5.693e-15 |
| 40 | TAR+ Area+AP+NDVI+ EVI | 5 | 386.6161 | 0.8564 | 0.8359 | 41.74 | 5 | 35 | 8.745e-14 |
| 37 | AP+AMT+NDVI+ EVI | 4 | 386.7069 | 0.8489 | 0.8321 | 50.55 | 4 | 36 | 2.754e-14 |
| 38 | Area+AP+NDVI+ EVI | 4 | 386.815 | 0.8485 | .8316 | 50.39 | 4 | 36 | 2.887e-14 |
| 25 | TAR+ Area+AP+AMT+NDVI | 5 | 386.9366 | 0.8553 | 0.8346 | 41.36 | 5 | 35 | 1.001e-13 |
| 57 | NDVI+AMT+Area | 3 | 387.3235 | 0.8389 | 0.8258 | 64.23 | 3 | 37 | 9.76e-15 |
| 44 | TAR+ Area+AP+AMT+NDVI+ EVI | 6 | 387.4548 | 0.8604 | 0.8 | 34.92 | 6 | 34 | 3.746e-13 |
| 17 | Area+AMT+NDVI+EVI | 4 | 387.9419 | 0.8442 | 0.8269 | 48.78 | 4 | 36 | 4.712e-14 |
| 24 | Area+AP+AMT+NDVI | 4 | 387.9579 | 0.8442 | 0.8269 | 48.76 | 4 | 36 | 4.745e-14 |
| 43 | Area+AP+AMT+NDVI+ EVI | 5 | 388.6269 | 0.8492 | 0.82 | 39.41 | 5 | 35 | 2.039e-13 |
| 6 | TAR+NDVI | 2 | 403.0749 | 0.7516 | 0.7386 | 57.5 | 2 | 38 | 3.213e-12 |
| 52 | TAR+NDVI+EVI | 3 | 404.4752 | 0.7552 | 0.7354 | 38.06 | 3 | 37 | 2.134e-11 |
| 12 | TAR+Area+NDVI | 3 | 404.9035 | 0.7527 | 0.7326 | 37.53 | 3 | 37 | 2.585e-11 |
| 16 | TAR+ Area+ NDVI | 3 | 404.9035 | 0.7527 | 0.7326 | 37.53 | 3 | 37 | 2.585e-11 |
| 31 | TAR+Area+NDVI+EVI | 4 | 406.4032 | 0.7557 | 0.7285 | 27.83 | 4 | 36 | 1.407e-10 |
| 35 | TAR+ Area+ NDVI+ EVI | 4 | 406.4032 | 0.7557 | 0.7285 | 27.83 | 4 | 36 | 1.407e-10 |
| 15 | TAR+ AP+AMT | 3 | 407.7058 | 0.7352 | .7137 | 34.24 | 3 | 37 | 9.053e-11 |
| 9 | AP+AMT | 2 | 408.3647 | 0.7174 | 0.7026 | 48.24 | 2 | 38 | 3.729e-11 |
| 34 | TAR+ AP+AMT+EVI | 4 | 408.3969 | 0.7435 | 0.715 | 26.09 | 4 | 36 | 3.325e-10 |
| 54 | AP + AMT +EVI | 3 | 408.846 | 0.7277 | 0.7056 | 32.96 | 3 | 37 | 1.507e-10 |
| 22 | TAR+ Area+AP+AMT | 4 | 409.4321 | 0.7369 | 0.7077 | 25.21 | 4 | 36 | 5.194e-10 |
| 41 | TAR+ Area+AP+AMT+ EVI | 5 | 409.9849 | 0.7461 | 0.7098 | 20.56 | 5 | 35 | 1.548e-09 |
| 20 | AMT+ Area+AP | 3 | 410.3518 | 0.7175 | 0.6946 | 31.33 | 3 | 37 | 2.954e-10 |
| 39 | AMT+ Area+AP+ EVI | 4 | 410.7825 | 0.7281 | 0.6979 | 24.1 | 4 | 36 | 9.293e-10 |
| 30 | TAR+Area+AP+EVI | 4 | 412.9077 | 0.7137 | 0.6818 | 22.43 | 4 | 36 | : 2.319e-09 |
| 1 | TAR+Area | 2 | 414.6254 | 0.6708 | 0.6535 | 38.72 | 2 | 38 | 6.785e-10 |
| 11 | TAR+Area+AP | 3 | 414.6261 | 0.6865 | 0.6611 | 27 | 3 | 37 | 1.991e-09 |
| 46 | TAR+Area +EVI | 3 | 414.6342 | 0.6864 | .661 | 27 | 3 | 37 | .998e-09 |
| 32 | TAR+Area+AMT+EVI | 4 | 414.8154 | 0.7 | 0.6667 | 21 | 4 | 36 | 5.263e-09 |
| 27 | NDVI+EVI | 2 | 415.1131 | 0.6669 | 0.6493 | 38.03 | 2 | 38 | 8.506e-10 |
| 3 | NDVI+Area | 2 | 415.224 | 0.666 | 0.6484 | 37.88 | 2 | 38 | 8.955e-10 |
| 13 | TAR+Area+AMT | 3 | 416.0573 | 0.6753 | 0.649 | 25.65 | 3 | 37 | 3.769e-09 |
| 49 | NDVI+Area+EVI | 3 | 416.9234 | 0.6684 | 0.6415 | 24.86 | 3 | 37 | 5.545e-09 |
| 48 | AP + Area +EVI | 3 | 420.1389 | 0.6414 | 0.6123 | 22.06 | 3 | 37 | 2.321e-08 |
| 29 | TAR+EVI | 2 | 424.0412 | 0.5858 | 0.564 | 26.87 | 2 | 38 | 5.328e-08 |
| 2 | AP+Area | 2 | 424.8524 | 0.5775 | 0.5553 | 25.98 | 2 | 38 | 7.76e-08 |
| 47 | AMT+Area+EVI | 3 | 425.1881 | 0.5943 | 0.5615 | 18.07 | 3 | 37 | 2.187e-07 |
| 51 | TAR+AP+EVI | 3 | 425.4073 | 0.5922 | 0.5591 | 17.91 | 3 | 37 | 2.411e-07 |
| 50 | TAR+AMT+EVI | 3 | 425.6024 | 0.5902 | 0.557 | 17.76 | 3 | 37 | 2.629e-07 |
| 26 | Area+EVI | 2 | 427.3431 | 0.5511 | 0.5275 | 23.32 | 2 | 38 | 2.461e-07 |
| 7 | TAR+AMT | 2 | 428.0624 | 0.5431 | 0.5191 | 22.59 | 2 | 38 | 3.435e-07 |
| 4 | AMT+Area | 2 | 429.2664 | 0.5295 | 0.5048 | 21.38 | 2 | 38 | 6.001e-07 |
| 5 | TAR+AP | 2 | 431.5756 | 0.5023 | 0.4761 | 19.17 | 2 | 38 | 1.75e-06 |
| 28 | AP+EVI | 2 | 440.4065 | 0.3826 | 0.3501 | 11.78 | 2 | 38 | 0.0001048 |
| 45 | AMT+EVI | 2 | 444.2985 | : 0.3212 | 0.2854 | 8.989 | 2 | 38 | 0.0006361 |
